# The Human Exposure Potential from Propylene Releases to the Environment

**DOI:** 10.3390/ijerph15010066

**Published:** 2018-01-04

**Authors:** David A. Morgott

**Affiliations:** Pennsport Consulting, LLC, 1 Christian Street, Unit#21, Philadelphia, PA 19147, USA; dmorgott@verizon.net; Tel.: +1-215-468-2506

**Keywords:** propene, inhalation exposure, emission factor, indoor, occupational, community, pyrogenic, biogenic, anthropogenic

## Abstract

A detailed literature search was performed to assess the sources, magnitudes and extent of human inhalation exposure to propylene. Exposure evaluations were performed at both the community and occupational levels for those living or working in different environments. The results revealed a multitude of pyrogenic, biogenic and anthropogenic emission sources. Pyrogenic sources, including biomass burning and fossil fuel combustion, appear to be the primary contributors to atmospheric propylene. Despite a very short atmospheric lifetime, measurable levels could be detected in highly remote locations as a result of biogenic release. The indoor/outdoor ratio for propylene has been shown to range from about 2 to 3 in non-smoking homes, which indicates that residential sources may be the largest contributor to the overall exposure for those not occupationally exposed. In homes where smoking takes place, the levels may be up to thirty times higher than non-smoking residences. Atmospheric levels in most rural regions are typically below 2 ppbv, whereas the values in urban levels are much more variable ranging as high as 10 ppbv. Somewhat elevated propylene exposures may also occur in the workplace; especially for firefighters or refinery plant operators who may encounter levels up to about 10 ppmv.

## 1. Introduction

Propylene (CAS#115-07-1) is a highly flammable colorless gas that is widely distributed in the environment. As a key commodity chemical, polymer and chemical grade propylene is manufactured and used in large amounts worldwide. Global production was predicted to increase from 109 million tons in 2014 to 165 million tons in 2030 [1]. Its primary usages include the production of polypropylene (64%) and the synthesis of downstream chemicals such as propylene oxide (7%), acrylonitrile (6%) and a variety of others (23%) including acrylic acid, cumene and isopropanol [2]. Propylene is produced via the steam cracking of petroleum feedstocks such as natural gas liquids and gas oils [3]. There are also numerous pyrogenic and biogenic sources of the propylene in the environment. These arise from photosynthetic pathways or the incomplete combustion of fuel hydrocarbons [4,5].

Although propylene exhibits some water solubility, oral exposures from contaminated drinking water are expected to be insignificant [6]. California’s Office of Environmental Health Hazard Assessment has established an 8-h chronic inhalation Reference Exposure Level (REL) of 3000 μg/m^3^ (1743 ppbv) for the general public [7]. Protective Action Criteria (PAC) values have been established at 1500, 2800 and 17,000 ppmv to protect against mild transient health effects, serious irreversible effects and life-threatening effects, respectively [8]. To protect against asphyxiation, the American Conference of Governmental Industrial Hygienists has established an 8-h time-weighted average occupational threshold limit value of 500 ppmv [9].

The following review summarizes available information on ambient air levels, emission factors and emission rates of propylene from known sources. The review is aimed at providing an overall picture of human exposure sources and the potential exposure magnitudes in both an occupational and community setting. The compiled information is also intended to be a resource for those involved with the creation of new human exposure models that can be used to conduct epidemiological evaluations and health risk assessments. To that end, a supplemental file has been included that chronologically lists the findings from ambient air monitor studies conducted throughout the world. This compendium of available information reveals that ambient air levels are generally about 3-fold lower than the levels found in occupational environments. 

## 2. Airborne Concentrations

Atmospheric monitoring of propylene has taken place at a wide variety of locations throughout the world. A survey of the literature yielded over 200 papers that included airborne measurements of propylene levels (see Supplemental Tables S1–S6). The sites ranged from dense population centers with heavy traffic burdens to remote locations with little human activity. In most cases, the measurements were taken near ground level and provide an estimate of the exposure concentration that would be experienced when traveling through that micro-environment. Aircraft measurements have also been examined to better gauge the background concentration of propylene in the free troposphere. Under normal circumstances propylene has a short lifetime in air due to its rapid photo oxidation in the presence of hydroxyl radicals. Values vary but most agree that the lifetime of propylene in ambient air is less than 10 h in general and can be as low as 0.8 h in urban areas [10]. The concentration of propylene in urban areas rarely exceeds 10 ppbv at urban sites and is often in the part per trillion range at remote or rural locations. A scan of measurements recorded in different regions of the world reveal that levels in Asia are generally higher than they are in North America or Europe.

Some monitoring studies have provided information on the horizontal and vertical spatial gradients that exist around an emission source. For instance, measurements taken near a highway in Raleigh, North Carolina found that propylene levels declined from 180 pptv at the surface to 13 pptv at an altitude of 250 m [11]. Further, the levels of propylene were undetectable at an elevation of 450 m. In another instance, measurable levels of propylene were found at altitudes greater than 4 km above Europe [12]. Horizontal propylene gradients have also been observed for specific emission sources. Roadway measurements of propylene at distances ranging from 0 to 80 m from a roadway revealed that the concentration declined abruptly from 3.5 ppbv to 0.5 ppbv, which approximated the propylene background concentration of 0.7 ppbv [13]. Studies in China found distinct diurnal variations in urban propylene levels with peaks during high traffic periods and nadirs late in the afternoon and evening [14]. These results were duplicated in a Korean study where the high urban propylene levels (2.5 ppbv) in the morning were followed by lower levels in the afternoon (0.9 ppbv) [15]. In Dallas, the atmospheric propylene levels began to rise at 4:00 then peak during rush hour at 6:00 to 7:00 before subsiding and peaking again at 20:00 [16]. These fluctuations occurred in conjunction with seasonal concentration changes that tended to be the highest in the autumn and lowest in the spring. Other studies, however, have shown that seasonal concentration changes are more in line with the expected declines during the summertime when the rate of photochemical oxidation is greatest. Such was the case in a monitoring program conducted in Rome where the average urban propylene levels declined from 4.05 ppbv in the winter months to 0.95 ppbv during the summertime [17]. In general, the winter-to-summer propylene concentration ranges from about 1.2 to 1.9 ppbv; however, values as high as 4.8 ppbv have been observed in some locations [18,19,20]. An examination of average weekday/weekend differences at a remote location in Spain found slight increases in propylene levels on the weekend with the values increasing from 0.08 ppbv to 0.10 ppbv [21].

There is good evidence showing local historical declines in tropospheric propylene levels in some regions of the world. An examination of average propylene levels in a rural area of the former East Germany documented an average monthly decrease of 0.03 ppbv over a 41 months period [22]. An examination of monitoring data from sites in and around Houston, Texas in the vicinity of olefin production plants found that the average annual propylene levels had declined at four of five locations over a ten year period from 2002 to 2012 [23]. The decrease in ambient air levels coincided with a decline in the statewide industrial emissions of propylene from 2002 (3251.4 tons/year) to 2012 (2167.6 tons/year). This type of change has also been observed on a more regional scale. For instance, the propylene concentrations in London, England have been declining by 20% per year since 2008 [24]. This large change was not seen, however, at a nearby rural location where the decline has been a more modest 6% per year. In a separate study, declines of 0.08–0.10 ppb/year were observed at rural and urban locations in the United Kingdom for periods of 5–6 years beginning in 1995 [25]. The declines were even greater for roadside measurements where 0.64 ppb/year were observed. Measurements taken in a major Dutch city over a ten-year period from 1981 to 1991 showed 2.9% decrease in propylene per year. Decreases observed in California’s South Coast Air Basin have largely been attributed to decreases in tailpipe emissions from light-duty gasoline-powered vehicles [26]. These findings are consistent with those from the Los Angeles air basin where the levels declined by 7.7% per year from 1960 to 2010 [27]. Similar declines have been observed in China with the levels in Beijing decreasing 6.3% per year over a nine year period from 2004 to 2013 [28]. 

## 3. Emission Sources

Despite its high atmospheric reactivity, background levels of propylene can be found in ambient air samples from virtually every location. This is primarily due to the large number of emission sources that exist. These include pyrogenic, biogenic and anthropogenic releases. Whereas, most studies consistently estimate that the total global release of propylene ranges from about 7 to 15 teragrams per year (Tg/year), some estimate emission rates are as high as 22.0–23.0 Tg/year [29,30,31]. These differences are partly attributable to disagreements on the relative contribution of biogenic emission sources to the total atmospheric burden. In most cases, the biogenic contribution to the total is thought to be about 1.5–7.6 Tg/year but in some cases a much higher value has been assumed to exist [32]. The primary biogenic sources of propylene include releases from soil, oceans and vegetation. By comparison, the global emissions from biomass burning has been estimated to be about 2.9 Tg/year [33]. The majority of these emissions are the result of agricultural waste burning and the use of wood as a fuel source [34]. Emission estimates of the anthropogenic propylene from industrial production and fossil fuel use range from about 1.0 to 2.7 Tg/year [35,36,37].

### 3.1. Pyrogenic Sources

Pyrogenic sources of propylene include biomass burning and the combustion of fossil fuels. The release of propylene during these events may be substantial especially in the vicinity of forest fires or heavy traffic. The propylene formed during these processes is the result of incomplete combustion of fuel hydrocarbons due to the absence of sufficient oxygen. The amount of propylene released from these sources is dependent upon many factors including the type of fuel and the combustion temperature. Propylene exposures would be expected to be higher than average for those working the vicinity of pyrogenic sources.

#### 3.1.1. Biomass Burning

A rich database is available describing the propylene releases associated with biomass burning. Field studies provide one source of information on the exposure levels of propylene that can be attained in the smoke plume arising from a biomass fire. For example, the average propylene concentration was 0.20 ppbv and the maximum 1.47 ppbv in flights through forest fire plumes originating in Canada [38]. By comparison, the median background concentration of propylene in clean air samples was only 3 pptv. Ground level measurements of propylene in the smoke plume from the burning of crop residues composed mainly of rice straw yielded levels of 0.98 ppbv, whereas the value outside the plume was 0.69 ppbv [39]. A controlled burn of forest debris resulted in a propylene concentration of 0.4 ppbv at a monitoring site 66 km from the fire [40]. Aircraft flights through a South African savanna fire found propylene levels as high 12 ppbv with clean ambient air yielding levels of 0.03 ppbv [41].

Forest fires, crop residue burning and underbrush fires can emit a large quantity of propylene to the atmosphere. In fact, the emission of propylene from biomass burning has been estimated to have increased from 1.76 Tg/year in 1960s to 3.51 Tg/year in the 1990s [42]. Those working in the vicinity of these fires would be expected to inhale above average amounts of propylene but the exact exposure magnitude is difficult to predict given the large number of variables affecting propylene production. Numerous investigators have published study results for the emissions of propylene from individual types of vegetation consumed in a prescribed burn or accidental fire. Some indication of the exposure concentrations that can be attained in the smoke plume of a biomass fire are provided by closed chamber experiments. These studies revealed that the air concentrations of propylene in the exhaust stack of a combustion chamber containing weed grasses could range as high as 8.8 ppmv under smoldering conditions [43]. Similarly, exhaust air concentrations of 4.1 ppmv propylene were seen with the experimental burning of barley straw [44]. Aircraft flights over a crop residue fire in Namibia detected propylene levels of 3.27 ppbv in the plume [45]. Exhaust air concentrations of propylene in four types of coal fired furnaces used for power, heat, coke and pig iron production ranged from 12.7 to 57.6 ppbv [46].

In recent years, investigators have begun scrutinizing all of the experimental information on hydrocarbon emission factors for different types of biomass burning. The goal was to publish composite emission factors that considered the results from all available field studies and laboratory test burns. These values provide the best available information for use in atmospheric models capable of predicting exposure levels in particular microenvironments. As shown in Table 1, the recommended emission factors range from about 380 mg/kg for chaparral to 2300 mg/kg for peat. These results are consistent with those from a survey of the emission factors resulting from thirteen different types of biomass fires in the North America [47]. The overall average factor of 630 ± 140 mg/kg was determined from aircraft measurements taken over fires that included chaparral, chamise, birch, poplar, Douglas fir, pine, spruce, aspen and sage. In general, the propylene emission factors determined from ground-based measurements agree well with those taken from flights through the plume. This was evident for a forest fire where values of 329 and 314 mg/kg were obtained for ground and airborne samples, respectively [48]. Similar results were also obtained when the values from a grass fire were compared. There is a difference, however, in propylene release depending on whether the fire is in the flame or smoldering stage. This deviation was observed in ground level measurements from an Amazonian forest fire and a pasture fire where a two-fold increase in the emission factor was observed for the smoldering stage [49].

As shown in Table 2, the release from different types of vegetation can often exceed 100 mg/kg of fuel [53]. A comparison of the values shows that some the highest emission factors were obtained with samples of peat from different regions of the world. When peat samples from the U.S. were independently tested in a research facility, factors of 800 and 1200 mg/kg values were obtained for the peat taken from two wildlife refuges in North Carolina [54]. The emission factor for smoldering sample of Indonesian peat was even higher and exceeded 3000 mg/kg of fuel [55]. These values are somewhat higher than the average factor of 1070 mg/kg observed after the ground-based sampling of 36 separate peat fires in Indonesia [56].

The compilation of values in Table 2 agree well with those from other investigators who have generally found average emission factors ranging from 700–1700 mg/kg for 21 different types of U.S. wood and vegetation burned in controlled laboratory experiments [57]. Test burns of wood and vegetation samples from California, Arizona and North Carolina yielded emission factors of 141, 97 and 288 mg/kg, respectively [58]. In some regions of the world including East Asia, crop residues can provide an important energy source in local homes. Experimental determination of the emission factors can provide useful information to gauge the potential impact of these fuels on indoor air levels. For instance, the combustion of rice straw and sugarcane residues in a test chamber resulted in emission factors of 749 and 380 mg/kg, respectively [59]. These values are somewhat higher than measurements recorded in a field investigation where the smoke plume was sampled from a test vehicle traveling around a prescribed burn [60]. Under these conditions, the propylene emission factors for wheat straw and maize stover were determined to be 120 and 210 mg/kg, respectively.

#### 3.1.2. Vehicle Emissions

Propylene is present in the exhaust stream of virtually every vehicle using a combustion type engine. Evidence exists for the presence of propylene in the exhaust stream of pleasure boats, passenger ferries and personal aircraft [61,62,63]. The biggest contributor to atmospheric propylene in most urban areas is the exhaust of passenger-type vehicles. The emission of propylene from vehicle exhaust has been repeatedly determined using one of three methods: road tunnel measurements, dynamometer testing, or roadside monitoring. Roadside measurements at busy intersections and highway off ramps found propylene concentrations ranging from 2.3–2.9 ppbv [64]. These same studies showed that the concentration of propylene in the vicinity of a busy roadway where a particular type of vehicle predominated could vary substantially with the values ranging from 1.2 ppbv for roadways with a high percentage of light and heavy duty diesel-powered commercial vehicles to 21.0 ppbv in a car park containing gasoline-powered private automobiles. Somewhat higher levels of propylene were found in an enclosed basement car park (5.72 ppbv) but not in a well ventilated outdoor car park (0.45 ppbv) [65]. Similarly, the propylene concentration in the vicinity of four gas stations during heavy rush hour traffic in Taoyuan, Taiwan ranged from 2.6 to 11.3 ppbv [66]. Long-term measurements of propylene near a roadway in Dublin, Ireland with a high percentage (85%) of gasoline-fueled cars found an average daily concentration of 1.66 ppbv over a six-week period [67].

Distinct spatial gradients exist for the exhaust emissions from motor vehicles with the air concentration decreasing as the distance increases. This was demonstrated in a roadway study showing that the levels of propylene declined from 0.72 ppbv at a distance of 13 m from a busy eight lane highway to 0.38 ppbv at a distance of 95 m [68]. The levels of propylene near heavily traveled highways may also be dramatically influenced by local meteorological conditions, especially wind speed and wind direction which can cause a 2-fold fluctuation in hourly propylene levels [69]. Taken together, these studies indicate that atmospheric dilution will influence the spatial and temporal variations in propylene concentration, especially in an urban environment where high traffic densities exist.

Regardless of the test conditions, propylene is typically one of the top ten most abundant hydrocarbons in automobile exhaust [70,71]. Depending on the vehicle, fuel and engine configuration, propylene can represent anywhere from 0.3 to 14.3% of the hydrocarbon emissions in the tailpipe exhaust [72]. The typical average weight percentage of propylene in the exhaust of a modern gasoline- or diesel-powered vehicle is about 2 to 5% of the total hydrocarbons [73,74]. These emissions occur despite the fact that little or no propylene is found in gasoline or diesel fuel. Studies have shown that 0.4% or less of the hydrocarbons in gasoline is composed of propylene [67,75,76]. There is no differentiation in the propylene content for summer and winter time fuel formulations with the weight percentage in gasoline or the headspace vapor hovering around 0.01 to 0.03% [77]. In contrast, propylene can constitute 0.2 to 1.8% of the hydrocarbons in the liquefied petroleum gas (LPG) used to power some vehicles [78]. The leakage of unburned LPG from the fuel tanks of automobiles was postulated to be a cause of the high propylene levels (39.2 ppbv) found near a busy highway in Mexico City [79].

Tunnel studies have been used to determine the vehicle emission factors for a host of hydrocarbons, including propylene. Unlike combustion studies where emissions are often expressed per unit mass (mg/kg), the emission factors from vehicle exhaust are given in terms of the distance traveled (mg/km) or the amount fuel burned (mg/L). Table 3 summarizes many of the tunnel studies with propylene emissions. The values indicate a propylene emission factor that generally ranges from about 5 to 15 mg/km; however, excursions as high as 30 to 40 mg/km have been noted. In some cases, the higher values may have been related to the collection of instantaneous rather than continuous samples. The concentration of propylene within the tunnels is often appreciably higher than the background ambient air levels and is often greater than 20 ppbv.

The propylene released into the exhaust stream from fossil fuel powered vehicles has been shown to be the result of incomplete combustion of aliphatic hydrocarbons in the fuel [95]. Exhaust emission factors have been determined for different vehicle types and traffic conditions. For instance, dynamometer testing of gasoline fueled passenger vehicles found that the exhaust emissions of propylene in automobiles manufactured from 1980 to 1985 was five times higher than those produced from 1986 to 1991 (96 mg/km versus 18 mg/km) [96]. These values are appreciably higher than the propylene emission factor of 1.84 mg/km obtained for a diesel-fueled automobile with an oxidation catalyst for pollution control [97]. Road testing of 125–150 cc gasoline-powered motorcycles equipped with a catalyst for pollution control and on board equipment for the sampling of the exhaust fume yielded a propylene emission factor of 32.0 mg/km when the test vehicles were driven on three different routes through the city of Taichung, Taiwan [98]. In another study, propylene emissions were not affected by a vehicle speed change from 25 to 100 km/h with gasoline and LPG-powered vehicles yielding values of 2 to 13 mg/km and 0.02 to 0.07 mg/km, respectively [99]. The average propylene emission factor for ten vehicles using either gasoline or LPG as a fuel was 0.96 mg/km and 2.22 mg/km, respectively [100]. The emission of propylene from the tailpipe of LPG-fueled taxis and buses was shown to decline by an average of 45.7% when they were equipped with catalytic converters [101]. Further, an examination of the impact of the automobile starting temperature showed that cold starts had a large impact on propylene emission factors. Using a gasoline powered vehicle with a three way catalyst, chassis dynamometer testing revealed an emission factor increase from 25 mg/km to 150 mg/km when the starting temperature was lowered from 22 °C to −20 °C [102]. Dynamometer testing of early model diesel-powered trucks and catalyst-equipped gasoline-powered automobiles indicated that the emissions of propylene was appreciably greater when gasoline was used as a fuel [103,104]. Emission factors of 14.9 and 0.8 mg/km were observed for the gasoline and diesel-powered vehicles, respectively. Studies examining the infiltration of exhaust hydrocarbons into an automobile cabin were not located, so the exposure implications of the high tailpipe emissions of propylene were not readily apparent.

### 3.2. Biogenic Sources

A substantial portion of the propylene found in ambient air is biogenic in origin. The biological and biochemical mechanisms responsible for its genesis have been reasonably well studied. Under most circumstances pyrogenic and anthropogenic emissions will dominate the exposure profile but under some conditions human contact with propylene may be dictated by an individual’s proximity to a natural source. The three most dominant biogenic sources of propylene are vegetation, oceans and soil, which are responsible for emitting approximately 7.5, 9.0 and 1.1 Tg/year, respectfully [29]. There is, however, considerable uncertainty with published estimates of the annual emissions of propylene from biogenic sources. It is not uncommon for some researchers to use values that far exceed those specified above. For instance, values as high as 10 Tg/year have been used to describe the annual release from vegetation [105].

#### 3.2.1. Vegetation

Several outdoor studies have shown elevated propylene concentrations in the vicinity of forests or agricultural fields. Measurements taken over a forest of red oak in New England showed increased propylene emission rates that were associated with solar radiation intensity; suggesting a photosynthetic source [106]. Support for the biogenic release of propylene from vegetation can be found in studies performed in boreal wetlands, commonly known as fens. Chamber measurements from a high dry site and low wet site covered by native mosses showed distinct changes in the emission rates over a period of months [107]. Peak emission rates of about 10 to 12 μg/m^2^/h were observed in August, while the rates in October were only 0–0.4 μg/m^2^/h. Further evidence of a link with photosynthesis came from the distinct diurnal variation which peaked in early afternoon and declined to near zero at night. Multiple summertime measurements at a Finnish fen found ambient air levels of propylene at 0.047 ± 0.015 ppbv [108]. Further study showed that the propylene emission rates ranged from zero to 0.37 μg/m^2^/h in the fen versus zero to 0.5 μg/m^2^/h in a nearby forest floor covered by mosses and shrubs [109]. The ambient air levels of propylene above the fen agree well with those found in a Borneo rainforest where the average concentration from multiple measurements was 0.047 ± 0.031 ppbv [110]. Moreover, field studies with the leaves of a birch tree showed that they emitted 0.08 μg/g/h of propylene but only during the budding period [111]. These findings are supported by others showing that the rate of propylene emission from an entire tree was greatest when it was blooming in May or June [112]. The peak emission rate for willow, birch and aspen trees at this time was 0.83, 0.05 and 0.6 μg/g/h, respectively. These results conflict somewhat with those showing that propylene was only emitted by the foliage from two of the seventeen trees tested [113]. European fir and Scots pine needles emitted an unquantified amount of propylene when enclosed in a polyethylene bag for several minutes.

Although the emission rates from vegetation are relatively low, these may lead to high concentrations under the proper conditions. The barge transport of wood pellets made from pine sawdust and planar shavings resulted in an appreciable accumulation of propylene in the cargo holds [114]. Following an ocean voyage from Canada to Sweden, the average concentration of propylene was 21.6 ppmv inside the cargo holds from five ships versus 12.0 ppmv on a stairway just outside the cargo hold. Other research findings have indicated, however, that only a slight concentration gradient exists for the propylene levels above soybean or maize fields; suggesting that there is no flux for these types of vegetation [115]. On the basis of these and other findings, some have suggested using an overall propylene emission rate of 0.05 μg/g/h for all types of trees when constructing air transport models [116].

#### 3.2.2. Seawater, Sediment and Snowpack

Propylene has routinely been detected in remote marine air samples collected from regions throughout world. Levels less than 0.20 ppbv have generally been observed during most cruises (see Supplemental Table S5). Evidence suggests that diurnal variations in airborne propylene from a clean air coastal site in Ireland was associated with the photochemical degradation of organic carbon in seawater [117]. Measurable amounts of propylene have also been detected in seawater samples. The average propylene concentration from 28 seawater sample datasets collected on seven different cruise campaigns taken before 1995 was 8.6 ± 9.2 ng/L [118]. Samples collected from the Arctic Ocean were found to contain an average propylene concentration of 3.84 ng/L; however there was considerable variability in the measurements that was attributed to differences in photochemistry at the different sites [119]. Depending on the latitude, the average concentration of propylene in seawater samples taken at sites across the mid-Atlantic ranged from 1.5–6.0 ng/L and the maximum emission rate from the seawater was 74.6 pg/cm^2^/h [120]. By comparison, the average yearly emission rate from the North Sea was determined to be 11.5 pg/cm^2^/h [121]. In a separate study, the average level of propylene in surface samples from the southern Indian Ocean was 1.40 ng/L and the emission rate to air was estimated to be 624 pg/cm^2^/h [122]. Propylene has also been detected in Antarctic sea ice at a concentration of 11.3 ng/L [123].

Seasonal cycles also exist for seawater propylene with the levels ranging from a low of 0.62 ng/L in January to a high of 15.84 ng/L in August at a sampling site off the coast of England [124]. The levels were all found to decrease as the sampling sites moved further off shore. The maximum summertime emission rate of propylene was 52.8 pg/cm^2^/h at a site close to shore. Sampling at different water depths in the eutrophic zone where light could penetrate failed to show a distinct gradient at five of six sites in the Atlantic Ocean and North Sea [125]. At a single site in the North Sea, propylene levels declined from about 3.8 ng/L near the surface to about 2.4 ng/L at a depth of 38 m. The results were attributed to differences in wind-induced vertical mixing and light intensity at the different sites.

Two photochemical mechanisms appear to be responsible for propylene found in seawater. The first involves dissolved organic carbon and the second centers on the photosynthetic pathways exhibited by some marine organisms. The former route appears to predominate and has been studied more intensively, whereas the latter route is more suspect. Experiments performed in the laboratory have shown that when the dissolved organic carbon found in seawater was irradiated with ultraviolet and short-wave visible light propylene levels increased at a rate of 0.224 ng/L/h [126]. In another set of experiments, the propylene generation rate was measured in unfiltered seawater samples that were either illuminated or kept in the dark [127]. The rate of formation increased from 0.051 ng/L/h for samples incubated in the dark to 0.069 ng/L/h for those that were irradiated with natural light. The authors attributed these differences to the presence of dissolved organic material that was released from dead and decaying algae. Further studies showed that the rate of propylene production was dependent on the source of the filtered seawater with the values ranging from 0.37 ng/L/h for samples from the Florida current to 12.9 ng/L/h for samples taken at the outlet of the Banana River [128]. The differences were attributed to the nature of the dissolved organic matter contained in each sample type.

Ocean sediment samples have routinely shown the presence of propylene, which was thought to arise from microbial action [129]. Sediment samples collected at seven sites in the South Pacific that included New Zealand and Antarctica contained propylene levels ranging as high as 0.21 μg/L of wet sediment [130]. In most cases, the concentration ranged between 0.01–0.11 μg/L but there was a distinct difference in levels observed at each site. The concentration varied in relation to the core depth, which did not exceed 384 cm and was typically between 40 and 200 cm. Very similar results were found in core samples taken from three sites in the Bering Sea, two sites in the Norwegian Sea and ten sites in the Pacific Ocean off the coast of Peru [131,132,133]. The highest propylene level at these locations was 621 μg/L for a site off the coast of Norway. Measurable amounts of propylene have also been seen in the interstitial water of sediment core samples collected up to about 1000 m off the Texas coast in the Gulf of Mexico [134]. Propylene concentrations ranging from 0.02 to 0.30 μg/L were observed in twelve core samples taken at depths up to 170 cm. The average concentration of propylene in the top 10 cm of sediment samples collected from an anaerobic region of San Francisco Bay was 0.30 ± 0.06 μg/L [135].

Interestingly, a similar phenomenon has been observed in polar snow packs. The concentration of propylene in snow samples taken from the Canadian arctic was 42 pptv, which was 1.4 times greater than the ambient air level [136]. The sampling of pore air inside of a snowpack in Greenland down to depth of about 2.0 m showed a distinct concentration gradient with the highest propylene levels of 89 pptv observed for samples collected near the surface [137]. The average propylene concentration at or near the surface of the snowpack was 6.2 pptv. The construction of a snow chamber showed that propylene production was measurably higher during periods when there was sunlight. A biological mechanism was not possible for the propylene release given the very cold temperatures. Instead, a complex photochemical pathway was suggested which involved the fragmentation of transported carbonyls. Many of these observations were confirmed in a subsequent study which showed a steep propylene gradient within the snowpack with the highest concentrations near the surface [138]. Summertime measurements of propylene levels in ambient air and the snowpack yielded values of 13 and 92 pptv, respectively.

Laboratory studies have also shown that the incubation of marine diatom and dinoflagellate cultures for at least 100 days resulted in a linear increase in propylene production [139]. Later studies have disputed these findings by showing that propylene was not produced by six strains of phytoplankton incubated at 23 °C [140]. More convincing evidence has been generated in field studies that examined the propylene production rate by several varieties of algae and microalgae collected from tidal rock pools off the coast of Ireland [141]. The maximum daytime concentration of propylene in two of these rock pools at low tide was 2.8 and 7.7 ng/L; whereas the average background concentration in local seawater was 0.7 ng/L. Further laboratory studies using cultured algae specimens found propylene production rates ranging as high as 0.8 ng/g/h for nine algae species. A net decrease in propylene levels was observed, however, with only one species of algae.

#### 3.2.3. Soil and Forest Debris 

Laboratory studies suggest that agricultural soils and forest debris may release considerable amounts of propylene and that the rates can be impacted by temperature, sunlight and microbiology. The emission rate of propylene ranged from 100 to 130 ng/kg/h for dried leaves sealed in a tube and heated up to 70 °C [142]. The emission rate was highest for ground sequoia leaves and lowest for whole rice grains. The release from whole and ground sequoia increased when the temperature was raised above 40 °C. In other studies, the emission of propylene from samples of whole and ground sequoia, ground rice and maize increased linearly as the intensity of ultraviolet light (280–320 nm) was increased up to about 7 W/m^2^ [143]. Longer wavelengths of ultraviolet light (320–400 nm) were not as effective in stimulating propylene emission from the test samples. A peak emission rate of about 3500 ng/m^2^/h was seen when ground rice samples were irradiated. Field studies in experimental rice paddies found propylene to be emitted at rates as high as 2.8 μg/m^2^/h; however, the authors attributed these emissions to photochemical processes occurring within the water column rather than foliage or soil release [144].

There has been research demonstrating that propylene can be released from agricultural soil and that its formation may be associated with particular bacterial strains. The emission rates from flooded and non-flooded rice paddy soil incubated for up to 56 days were 1.73 and 1.88 ng/kg/h, respectively [145]. These values increased dramatically when rice straw was added to the soil as a fertilizer. Incubations under these conditions released propylene at a rate of 18.4 ng/kg/h for flooded soil and 76.2 ng/kg/h for non-flooded soil. Further studies found that there was a statistically significant association between propylene emissions and the presence of *Chthoniobacter flavus* and *Flavitalea populi* in the flooded soil and *Desulfitobacterium dehalogenans* and *Methylacidimicrobium tartarphylax* in the non-flooded soil [146]. Separate studies have shown that propylene levels up to 0.43 μg/L could be found in the headspace when *Desulfovibrio desulfuricans* and two species of cyanobacteria from golf course soil were incubated for 5 weeks [147]. Although it is far more common to find bacterial species capable of utilizing propylene as an energy source, several species of *Dehalococcides* have been identified with the ability to biodegrade halocarbons with the formation of propylene as an end product [148,149,150].

### 3.3. Industrial Emissions

Industrial emissions of propylene can occur as a result of either unintentional fugitive releases or controlled flaring. The production and use of propylene at a petrochemical or olefins plant generally takes place in closed and contained systems that minimize any releases to the atmosphere. Equipment failures and leakages from valves, pumps and flanges can, however, result in the fugitive release of propylene at these sites. Flaring involves the combustion of waste gases to carbon dioxide to minimize their contribution to local ozone levels. Uncontrolled flaring can also occur at mining sites to prevent the direct release of waste gases to the atmosphere. Industrial propylene emissions may lead to locally higher concentrations that are above background. Supplemental Tables S1–S4 provide information on the airborne measurements that have been taken at or near the fence line of industrial facilities releasing propylene. These studies indicate that airborne levels in the vicinity of petroleum refineries, oil fields, polypropylene manufacturing sites, or olefin plants are generally less than 10 ppbv.

The flaring of waste gasses is generally episodic and occurs on an irregular and unpredictable schedule depending on the start-up or shut-down status of a particular facility [151]. Flare combustion efficiencies are generally in the vicinity of 99% but changes in the gas flow rate or the steam-to-gas fuel ratio can lead to values as low as 84% [152,153]. The percentages of propylene in waste gases destined to be flared at a petroleum refinery or olefins plant ranged from 2.26 to 13.14% [154]. A comparison of total hydrocarbon emissions from a Taiwanese petroleum refinery found that fugitive releases far out-paced flare releases (474.7 ton/year versus 10.2 ton/year) at a cracker plant [155]. Early 2006 measurements of propylene in an area around the Houston Ship Channel where a large number of petrochemical complexes are located found the emission rate to be 2140 kg/h [156]. Some short duration releases can result in an emission rate as high as 17,606 kg/h [157]. Another determination in the same region reported propylene emission rates of 3.8 and 124.5 kg/h in two separate flares [158].

The total industrial air releases of propylene in the US has declined from 492.8 metric tons in 2005 to 255.6 metric tons in 2015 [159]. The emission rate of propylene from six industrial areas in and around Houston, Texas have shown a decline at three of the locations for a 7-year period from 1999 to 2006 [160]. These changes are partly attributable to the availability of new remote sensing equipment to detect propylene leakage from point sources within a plant site. The minimum detection level for propylene using an open-path Fourier-Transform Infrared spectrometer can be as low as 1 ppbv [161]. The total release of propylene can be substantial under some conditions. For instance, 11.7% of the hydrocarbon release was propylene at a large Chinese petrochemical site that included a petroleum refinery along with an olefin plant and a polypropylene manufacturing plant [162]. Fugitive emissions can result in somewhat higher ambient air concentrations beyond the fence line relative to background air. For instance, levels downwind of an oil refinery operating in the late 70’s did not exceed 41 ppbv [163]. These levels are not, however, reflective of the gains that have been made in reducing the emissions of propylene. A good example of this change includes the observed 5-year (2006 to 2010) declines of 56% and 64% for the propylene measurements from two monitoring stations near the Houston Ship Channel [164]. The preceding studies suggest that those individuals living in the vicinity of a petrochemical plant may at times be exposed to locally higher levels of propylene; they would not be expected to be greater than 10 ppbv under most circumstances.

### 3.4. Miscellaneous Sources

The emission factor for propylene from the combustion of organic soil in a test chamber was reported to be 1220 mg/kg whereas the value for garbage containing no food waste was 847 mg/kg [51]. The value for garbage agrees well with the average emission factor of 1258 mg/kg calculated from ground based measurements and airborne flights over two garbage fire plumes in Mexico [165]. By comparison, the emission factor for the combustion of plastic bag waste was determined to be 442 mg/kg [50,53]. The combustion of dried tomato plant material in a residential woodstove reportedly released 190 mg/kg of propylene [166]. The emission factor for the extrusion of low density polyethylene at a temperature of 600 °F was 0.38 mg/kg [167]. The pyrolysis of plastic mobile phone cases at a temperature of 850 °C gave a propylene emission factor of 3091 mg/kg [168]. The factor for the pyrolysis of printed circuit boards from mobile phones at 600 °C was 2070 mg/kg [169]. Propylene emissions were detected but not measured in samples taken from urban waste disposal bins [170]. The burning of garbage in open landfills gave an average emission factor of 1260 mg/kg [171]. The factor for the open burning of simulated military waste that included plastics, wood, metals, fabrics, paper, food waste and other materials was 1700 mg/kg of carbon burned [172]. Test burns of newsprint in a ceramic vessel that intensified the resulting smoke yielded a propylene concentration of 14.6 ppmv directly above the fire [44]. Small, but measurable, amounts of propylene were found to be released by decomposing human cadavers [173]. The emission rate of propylene from the cover soil at a French landfill handling municipal solid waste ranged from 1.62 to 9.25 μg/m^2^/day at various locations [174]. Elevated levels of propylene in the range of 6–8 ppbv have been observed in the gas discharges from volcanic fumaroles [175].

## 4. Indoor Air

Indoor-to-outdoor (I/O) concentration ratios of propylene provide insight into population level exposures and the relative contribution of propylene from emission sources within the home or office. Since people living in North America spend about 65% of their time at home, the indoor levels of propylene can play a large role in governing the overall exposure magnitude, especially if levels found indoors exceed those found in outside air [176]. There are, however, a large number of variables that can affect the exposure levels inside of homes including the presence of tobacco smoke, the type of cooking and heating appliances used and the types of food prepared.

### 4.1. Indoor to Outdoor Ratios

A key to estimating human exposure to propylene at the community level lies with a determination of the I/O ratio for residences, office buildings and schools. As Table 4 shows, the I/O ratio for propylene has often been found to exceed 2.0 and values greater than 8.0 have been observed in several instances. Since many of these studies were performed in non-smoking households, this condition was not an important contributing factor. In fact, the I/O ratio of propylene in the homes of smoking adults has been shown to be about 30 [177]. This value is higher than the I/O ratio of 9 observed in a smoky tavern but is consistent with the high levels of propylene that can be attained with cigarette smoking [178]. Experiments in environmental test chambers have detected propylene levels ranging from about 27 to 61 ppbv in side-stream cigarette smoke [178,179]. Not surprisingly, main-stream cigarette smoke contains very high levels of propylene that can range from 163 to 660 ppmv depending on the brand [180]. Although propylene can be detected in human blood and can be excreted through the skin and into the expired air, their contribution to indoor air levels is difficult to determine [181,182,183]. Studies have also shown that homes with attached garages can impact indoor propylene levels and that outdoor air infiltration may in some cases be important when there are oil and gas industries located nearby [184,185]. Some investigators have reported a propylene emission factor of 1370 μg/h in homes with both children and adults in residence [186]. Other studies found that the I/O ratio of 2.9 in homes occupied by adults was not appreciably different than the ratio of 3.2 observed in residences occupied solely by children [187]. Using detailed mathematical techniques, the geometric mean emission rate of propylene in a standard home was estimated to be 0.24 ppbv/h [188]. A recent survey of 50 non-smoking residential buildings in Montana found average propylene levels of 3.5 ppbv and a detection frequency of 96% [189]. The upper 95th percentile of measurement distribution was 20.3 ppbv. The levels of propylene inside four elementary schools located in a highly industrialized region of Taiwan ranged from 1.09 to 1.65 ppbv [190].

### 4.2. Food Preparation

The single biggest source of indoor propylene other than outdoor migration seems to revolve around food preparation. Studies have consistently shown that food cooking with any of a variety of fuels can lead to the release of substantial amounts of propylene. The propylene that is generated has been related to the incomplete combustion of both the foodstuffs and the fuel used during cooking. A comparison of propylene levels found in kitchens of two Hong Kong apartments that prepared the same deep fried, stir-fried, steamed and boiled meals found that the mean concentration directly above the stove was 15.5 ppbv in the apartment using coal gas as a fuel and 102.8 ppbv in the dwelling using LPG [198]. The large difference in propylene levels was attributed to discrepancies in the background levels of propylene measured before the cooking began, which were found to be 0.2 and 36.4 ppbv for the apartments using coal gas and LPG, respectively. Measurements of the hydrocarbon content of these two fuels failed to show the presence of detectable levels of propylene.

Although these data suggest that differences may exist in the combustion characteristics of the two fuels, they also provide good evidence that incomplete combustion of the foodstuffs used in the study may also have been responsible for the relatively high exposure concentrations. This was confirmed in two studies focusing on the emissions observed during the preparation of different Chinese cuisines. In the first study, propylene levels ranging from 17.4 to 31.6 ppbv were found in the stove exhaust air from four different Beijing restaurants [199]. The highest propylene levels were observed in restaurants specializing in Korean barbecue that was charbroiled over charcoal or natural gas. The propylene levels resulting from the preparation of American style barbecue were reported to be twice as high as Chinese barbecue and accounted for approximately 30% of the total hydrocarbon emissions. This value is far greater than the propylene emission percentage of 2.8% observed in Mexican restaurants [200]. The emission factor for meat charbroiling over a natural gas fired grill has previously been shown to be 289 mg/kg [201]. In the second study, slightly higher values were observed in three of four Chinese restaurants cooking different styles of cuisine [202]. In this instance, the levels ranged from 22.2 to 49.9 ppbv in sites cooking over natural gas or LPG. As before, much higher levels of 389 ppbv were found in the exhaust vent of a barbecue restaurant cooking over charcoal. 

These results are consistent with independent experiments showing that glowing charcoal could result in propylene levels ranging from about 20 to 1000 ppbv depending on the source of the fuel [203]. These studies demonstrate that the cooking of foodstuffs may have an impact on the indoor air levels of propylene; however, the relative contribution is a function of the fuel type. Emission factors for different fuels can vary dramatically ranging from less than 5 mg/kg for natural gas and coal briquettes to over 400 mg/kg for coal powder [204]. In contrast, the use of wood, kerosene and LPG in cook stoves resulted in emission factors of 22.3, 34.7 and 5.4 mg/kg, respectively. The release of propylene has also been observed when more basic cooking fuels such as brushwood or straw were used as cooking fuels in a rural setting [205]. The emission factor when using dung as a cooking fuel was determined to be 1890 mg/kg [50]. These findings, taken together, indicate that the activities surrounding meal preparation may be a major source of the higher than expected I/O ratio for propylene.

### 4.3. Appliance Use

Other investigations have showed how the type of appliance could also impact the emission of propylene. The exhaust stack concentration of propylene from a high efficiency residential wood boiler using birch wood as a fuel was 29 ppbv during the initial flaming stage [206]. Chamber testing of French made kerosene space heaters using a wick or injector for flame production yielded air concentrations of 19.2 and 6.6 ppbv, respectively [207]. The combustion of birch, spruce or alder in five different types of Finish heaters found the highest emission factor of 1370 mg/kg in a sauna stove and the lowest factor of 130 mg/kg in a small room stove [208]. Lower emission factors ranging from 42–125 mg/kg were observed when birch and pine logs were burned normally in a Swedish wood stove [209]. Small outdoor cook stoves have also been seen to release measurable quantities of propylene, depending on the type of fuel. Using red oak, okote, or Douglas fir wood as a fuel, the emission factors for three different camp stoves ranged as high as 12 mg/kg compared to 58 mg/kg for an open camp fire [53]. Modern indoor stoves burning pine and spruce sawdust pellets exhibited similarly low emission factors of 4 to 21 mg/kg [210]. The combustion conditions for these pellets can, however, impact the generation of propylene [211]. When smoldering, the pellets resulted in exhaust air concentration of 2.4 ppmv, whereas when aflame the level was only 0.2 ppmv. The values can also vary as a function of their composition with smoldering pellets made from oats, wheat straw, peat and sawdust yielding exhaust air levels of 2.1, 3.4, 4.5 and 0.8 ppmv, respectively [212,213].

The use of home heating fuels is not a likely source for the elevated indoor air levels, since many studies have failed to show an appreciable difference in I/O ratios for summer and winter [5]. The exception would be homes using wood burning fireplaces or stoves for heating, or residences that employee coal or some other basic biogenic fuel for heating. In both of these cases, measurable amounts of propylene have been shown to be generated. For instance pine wood burned in a residential fireplace was a major contributor to the total hydrocarbon emissions yielding a propylene emission factor of 429 mg/kg of wood [214]. In a separate study, emission factors 1070 and 2529 mg/kg were found when oak was burned in an American-made fireplace or woodstove, respectively [215]. Further, propylene was shown to account for about 7.5% of the hydrocarbon emissions when coal was burned in a residential stove from China [216]. In this instance, emission factors of 392 and 43 mg/kg were determined when the coal smoldered or burned with a flame, respectively. A comparison of the average emission factors for alternative fuels showed that briquettes made from waste straw and peanut shells was 1696 mg/kg versus 10 mg/kg for bituminous and anthracite coals [217].

### 4.4. Miscellaneous Indoor Sources

Some investigators have stated that propylene can be used as a propellant in consumer products or emitted from commercially marketed air fresheners but these uses are neither widespread nor important from an exposure perspective given the limited use of propylene in consumer and commercial products [5,6]. Likewise, tests showing that propylene was a good alternative refrigerant for use in residential air conditioners have not resulted in widespread commercial use [218]. Some compelling evidence has been published showing that indoor propylene levels may be influenced by the presence of ornamental house plants or other types of vegetation. In most instances, however, the release from indoor vegetation is not expected to a major source for the excess propylene that is found. Some documents have stated that propylene can be emitted from stored fruits and vegetables; however this information is difficult to substantiate since the original research cannot be located or verified [5,6]. In fact, a thorough search of the literature failed to identify any studies showing that propylene was emitted from any farm products. One exception was garlic cloves, where a single study purportedly detected the presence of propylene after the cloves were macerated and placed in boiling water for 2 h [219]. It is highly likely that the propylene found to be present was a contaminant that originated during sample preparation.

## 5. Human Exposure

There have been few measurements of personal exposure to propylene either in a community or occupational setting. Despite the absence of an appreciable number of measurements, some reasonable approximations can be made of the exposure magnitude for many populations. This exercise often involves the creation of an exposure model that utilizes available information on potential emission sources, rates and volumes to supplement what is known about the local environmental concentration. The goal of an exposure model is the estimation of the breathing zone concentration over a particular time period. To be of practical value, the model needs to consider the temporal and spatial concentration-related changes that occur over the time period of interest. Exposure modeling is particularly valuable in environmental epidemiology where the best possible estimates are needed when establishing whether a relationship exists with a particular acute or chronic health effect. The older approach of using the measurements from centrally located monitoring stations to estimate the exposures for individuals living in the surrounding areas has fallen out of favor because of the inherent bias it generates [220]. Newer methods that take into consideration time-activity patterns, microenvironmental concentrations and indoor/outdoor ratios have provided a more robust approach to exposure modeling. Examples of this approach include STEMS (Space-Time Exposure Modeling System) which uses meteorology, emissions data and time activity patterns to generate an exposure profile for an individual whose movement is tracked via geographic information systems [221]. Other advanced exposure modeling techniques involve the creation of land use regression models, hybrid models and dispersion models [222]. Although these methods have not been specifically used to estimate propylene exposures in the general population, it is likely that they will be in the future.

Estimates of human exposure to propylene have thus far been somewhat rudimentary since these have extrapolated the measurements from a few stationary monitoring sites to all of the residents within the population of interest. This was the approach used in an epidemiology study of cancer incidence for those living near a petrochemical plant in Sweden [223]. The estimated exposures to propylene in the low and high exposure groups were 0.23 and 0.64 ppbv, respectively. In a separate examination of the relationship between propylene exposure and cardiovascular mortality, the exposure concentration for a group of 66,534 individuals was estimated to be 1.7 ± 0.75 ppbv based on the measurements from a single centrally located monitoring site in Taichung, Taiwan [224]. The airborne levels in a residential area just beyond the fence line of a now closed petroleum refinery in Taiwan ranged from a low of 3.9 ppbv in the afternoon to a high of 61.4 ppbv in the morning [225]. Based on measurements from a mobile monitoring vehicle traveling in areas with high and low propylene emissions within Stockholm, the average exposure was determined to be 1.3 ppbv after considering the time-activity patterns within specific microenvironments [226].

The importance of indoor air levels on personal exposure to propylene was demonstrated in a group of 48 non-smoking participants living in Windsor, Canada [187]. The overall average daily exposure during the summer and winter months was 0.86 and 0.78 ppbv, respectively. These values agree well with indoor residential measurements which were 0.89 and 0.76 ppbv for the summer and winter months, respectively. The personal exposures were also 2 to 3-fold higher than the concentrations found outdoors. The 1-h personal propylene exposures of 38 non-smoking bicyclists traveling through the streets of Ottawa, Canada have also been determined [227]. The levels for low and high traffic 10 km bicycle routes were 0.17 and 0.52 ppbv, respectively. The concentration range for the high traffic route was 0.12–1.74 ppbv. An early Swedish study used area sampling to determine propylene levels inside commuter trains and diesel buses [228]. The average level in local and commuter diesel-powered buses was 3.9 ppbv, whereas the value in commuter trains was 2.0 ppbv. These studies indicate that non-occupational human exposure to propylene would be expected to be less than 10 ppbv for a 24-h period. Because propylene is a ubiquitous hydrocarbon that can be detected in even the most pristine environments, exogenous exposures are both continuous and unavoidable.

The highest occupational exposures to propylene would be expected for those individuals working in the vicinity of high traffic areas, power plants, furnaces, incinerators, internal combustion engines, open fires, oil wells and olefin crackers. Available data indicates that occupational exposures in excess of the 8-h time-weighted average occupational exposure limit of 500 ppmv are very unlikely [9]. This is based on available data from sites where propylene emissions are known to occur. For instance, the maximum levels of propylene observed atop a coke oven battery at a steel mill were found to be 458 ppbv [229]. Measurements taken at four processing locations inside a Taiwanese petrochemical plant yielded values ranging from 25 to 764 ppbv [230]. The highest levels were found in an area around the naphtha cracker. In an extended follow-up study at the same petroleum refinery, air levels up to 1504 ppbv were found near a naphtha cracker and levels up to 8025 ppbv around the tank farm [231]. The maximum daily average propylene concentration was 1.4 ppbv in area measurements taken in close proximity to four natural gas wells [232]. The average concentration of propylene in air samples collected upwind and downwind of a major industrial complex in Alberta, Canada containing over 40 oil or gas-related processing facilities was 0.06 and 20.39 ppbv, respectively [233]. Samples collected 100 m downwind of a catalytic cracker at a petroleum refinery in Sweden found local ambient air levels of 12.0 ppbv [234]. This value is in agreement with measurements taken in the vicinity of catalytic cracking and catalytic reforming units at a Chinese petroleum refinery where the levels were approximately 6.1 and 3.0 ppbv, respectively [235]. The average propylene level found in area samples collected in the vicinity of a Taiwanese naphtha cracker was 200 ppbv, whereas the average level around the oil storage tanks was 61.3 ppbv [225]. In another study the average concentration of propylene at the top of a 15 m building inside a large petrochemical complex in Taiwan was about 6 ppbv [236]. Recent measurements of propylene in the vicinity of a Chinese polypropylene manufacturing site yielded a value of 18.1 ppbv [162]. The levels of propylene near the fence line of the Eagle Ford shale oil mining site near San Antonio were found to range up to about 1 ppbv [237].

Measurements indicate that professional firefighters are exposed to high levels of propylene but good estimates of the exposure magnitude are unavailable. Samples collected during the knockdown phase of a residential house fire reported found 2 ppmv of propylene [238]. The maximum propylene levels observed in areas around nine municipal structural fires was 21.6 ppmv and the average level was 5.0 ppmv [239]. Test fires in intentionally burned automobiles showed that the levels of propylene were highest during the initial startup phase of a cabin fire (10.5 ppmv) and final overhaul phase of an engine fire (6.4 ppmv) [240]. Measurements during a training firefighting exercise showed that the propylene levels decrease from about 4.5 ppmv during the active burning phase to about 20 ppbv during the overhaul phase when the remaining embers were extinguished [241]. A small, but detectable, increase in propylene was also observed off-gassing from the gear worn during the fire. A portion of propylene generated in these fires may have resulted from the pyrolysis of polypropylene since studies have shown that it was liberated in substantial amounts when the pyrolysis temperatures exceeded 400 °C [242]. Chamber measurements during test burns with polyurethane foams showed the presence of propylene at levels up to 9.6 ppmv when the test sample contained fire resistant cotton in combination with a polyurethane foam [243]. Test burns of other household goods in the concrete basement of an abandoned home found maximum 15 min propylene levels of about 1.9 and 1.2 ppmv from a bed mattress and cardboard boxes, respectively [244].

In addition to firefighters and refinery operators, employees in other occupations would be expected to receive somewhat elevated intermittent exposures to propylene. There are several studies supporting above average exposures for individuals working in a broad range of occupations. The sampling of air from gas vents at a Florida landfill yielded an average value of 2.0 ppmv [245]. At another site, propylene levels ranging from 2.8 to 4.4 ppbv were found in the air from gas collection stacks placed deep within two French landfills [174]. The maximum concentration of propylene seen at 10 locations around a fossil fuel-burning power plant in Kuwait ranged from 3.2 to 15.9 ppbv [246]. Measurements made in two municipal sewer systems for a city in China found average propylene levels of 3.7 and 6.9 ppbv [247]. The level in a smoke plume generated by the uncontrolled burning of shredded tires in a landfill was 5.54 ppbv at 300 m from the fire; whereas, the level in nearby background air was less than 0.16 ppbv [248]. The propylene levels inside a commercial poultry production building ranged as high as 39.2 ppbv, whereas the values in a nearby empty building did not exceed 1.3 ppbv [249].

## 6. Conclusions

The preceding review provides a detailed look at the factors and circumstances affecting human inhalation exposures to propylene. It includes a thorough examination of ambient monitoring studies that have taken place both inside and outside the home. Other microenvironmental exposures have also been evaluated including those associated with employment, commuting and school attendance. The analysis shows that there are a large number of potential emission sources for propylene. These can be placed into three primary categories: pyrogenic, biogenic and anthropogenic. Pyrogenic sources, including biomass burning and fossil fuel combustion, are perhaps the biggest contributors to propylene exposure at the global scale. Second hand smoke can lead to propylene exposures that are 30 times higher than the background levels found within the home. Those traversing smoke plumes from forest fires or the burning of agricultural waste will receive propylene exposures that are several hundred-fold higher than those from background air. People living in the vicinity of a petrochemical plant may also experience exposures greater than background but in this case, the exposure level will be impacted by the magnitude of any fugitive releases and flaring that occurs.

Human inhalation exposures to propylene are commonplace and occur to some small degree in even the most remote environments. In most situations, the exposures are in the part-per-billion level or lower in urban locations where numerous emissions sources may exist. There is considerable variability in these numbers depending on proximity to local point and area emission sources such as automobile exhaust, burning biomass and industrial emissions. Community level exposures may be strongly affected by the amount of time spent indoors, since the indoor/outdoor ratio for propylene ranges from 2 to 3. It is difficult to differentiate the exposure magnitude for those living in an urban versus a rural environment because of local meteorological and traffic-related influences; but a 2 to 3-fold difference generally seems to exist with much higher disparities possible depending on the circumstances. Rural levels in most regions of the world are typically less than 2 ppbv and are in many cases in the pptv range. Urban levels can vary dramatically but are generally less than 10 ppbv.

Elevated propylene exposures may also occur in the workplace; however, the evidence suggests that these rarely if ever approach the 8-h threshold limit value of 500 ppmv. In fact, it appears that short-term occupational exposures to propylene are 10 ppmv or less under most circumstances. Those individuals working as firefighters and production workers would be expected to receive the highest exposures, but the levels are both intermittent and variable. Other occupations associated with higher than background levels of propylene include operators at municipal incinerators, smelters, power and pyrolysis plants, landfills, toll plazas and commercial farming sites. Although propylene exposures are both continuous and omnipresent for all individuals of every age, the exact levels are difficult to precisely predict given the variable contribution from a wide range of emission sources. 

## Figures and Tables

**Table 1:** Biomass fire emission factors compiled from studies conducted both in the field in the laboratory [50,51,52].

Type of Fire	Emission Factor (mg/kg)	Standard Deviation
tropical forest	640	430
savanna	790	560
crop residue	680	370
pasture maintenance	850	660
boreal forest	1130	600
temperate forest	950	540
peat land	2300	740
chaparral	380	130
semiarid shrub land	532	216
pine forest understory	405	277
coniferous canopy	497	228
deforestation	500	NR *
pasture maintenance	840	NR

* NR—not reported.

**Table 2:** Laboratory measurement of propylene emission factors for different types of biomass [53].

Fuel Type	No. of Samples	Emission Factor (mg/kg)	Standard Deviation (mg/kg)	Fuel Type	No. of Samples	Emission Factor (mg/kg)	Standard Deviation (mg/kg)
African grass	7	86.0	25.8	Indonesian peat	4	1352.7	829.2
Organic alfalfa	5	1238.1	358.9	U.S. peat	3	1421.1	502.6
Black spruce	5	545.3	218.9	Ponderosa pine	9	1332.6	381.6
Chamise	7	583.5	306.8	Rice straw	9	457.2	305.4
Giant cutgrass	7	176.0	103.2	Saw grass	12	140.9	108.6
Organic hay	7	498.5	141.6	Sugar cane	4	681.9	163.5
Juniper	3	268.6	171.1	Conventional wheat straw	5	177.5	57.5
Manzanita	1	494.8	NR *	Organic wheat straw	5	225.4	83.2
Canadian peat	1	484.7	NR	Wiregrass	5	69.8	38.0

* NR—not reported.

**Table 3:** Propylene concentrations and exhaust emission factors found in tunnel studies.

Location	Inlet Conc. (ppbv)	Outlet Conc. (ppbv)	Emission Factor (mg/km)	Salient Conditions	Reference
Taipei, Taiwan	NR	NR	11.61 ± 2.04	0.8 km two bore tunnel with two lanes per bore; two blowers enhance the natural ventilation; light and heavy-duty gasoline and diesel-powered vehicles	[80]
Gubrist, Switzerland	NR NR	NR NR	13.93 ± 3.81 (gas) 22.43 ± 19.38 (diesel)	3.4 km single bore tunnel with two lanes per bore; light and heavy-duty gasoline and diesel-powered vehicles	[81]
Hong Kong, China	4.5 ±1.1	6.9 ± 1.9	5.3 ±1.5	1.6 km two bore tunnel with exhaust fans but no mechanical ventilation; 47% diesel, 43% gasoline and 10% LPG powered vehicles	[82]
Monterrey, Mexico	NR NR	NR NR	13.66 ± 2.09 ≈ (bore 1) 6.63 ± 2.56 ≈ (bore 2)	0.53 km two bore tunnel with 4 lanes per bore; moderate traffic density; 97% gasoline and 3% diesel fueled vehicles, trucks, buses and motorcycles	[83]
Paris, France	NR	40.3 ± 9.2 *	38.03 ± 14.29	0.6 km two bore tunnel with 2 lanes per bore; mechanical ventilation not used; 94% light-duty and 6% heavy duty vehicles	[84]
Baltimore, Maryland	NR NR	NR NR	8.33 ± 0.67 (LD) 15.04 ± 3.51 (HD)	2.7 km 4 bore tunnel with 2 lanes per bore; LD vehicles included cars, pickup trucks, motorcycles and utility vehicles; HD vehicles included buses and tractor trailers	[85]
Tuscarora, Pennsylvania	NR NR	NR NR	5.76 ± 0.65 (LD) 9.95 ± 2.13 (HD)	1.8 km 2 bore tunnel with 2 lanes per bore; LD vehicles included cars, pickup trucks, motorcycles and utility vehicles; HD vehicles included buses and tractor trailers	[85]
Milwaukee, Wisconsin	NR NR	NR NR	7.03 ± 2.28 # (bore 1) 9.38 ± 1.65 # (bore 2)	2 separate tunnels of unknown length; the first has a single bore single lane with forced mechanical ventilation; the second tunnel has two bores with three lanes per bore and no mechanical ventilation; over 90% of the vehicles were automobiles or LD vans, pickup trucks and SUVs	[77]
Oakland, California	NR NR	NR NR	11.5 ^ (gas) 9.2 ^ (diesel)	1.1 km four bore tunnel with 2 lanes per bore; weekday traffic density of about 200 light duty and 30–140 medium and heavy duty das and diesel-powered vehicles per hour	[86]
Southern Taiwan	NR NR	5.8 ± 5.1 ‡ (bore 1) 10.9 ± 7.7 ‡ (bore 2)	10.36	1.8 km two bore tunnel with 3 lanes per bore; natural ventilation caused by traffic flow; LD vehicles 85%. HD vehicles 15%	[87]
Sydney, Australia	NR	83.2 ± 18.4 †	ND	2.3 km single bore with 4 lanes; ventilated with 14 air supply fans; 73.7% passenger vehicles, 9.9% tractor trailers, 11% utility vehicles and vans, 5.4% motorcycles and buses.	[88]
Seoul, Korea	ND ND ND	25.0 ± 3.3 † (Sp) 18.8 ± 15.4 † (Su) 15.3 ± 6.6 † (Wi)	ND	566 m two bore tunnel with 2 lanes per bore; natural ventilation; gasoline fueled 50–70%, diesel fueled (20–30%) and butane fueled (10–20%) vehicles	[89]
Yilan, Taiwan	5.5 ± 2.5 (bore 1) 7.7 ± 4.3 (bore 2)	44.2 ± 11.6 (bore 1) 41.4 ± 12.8 (bore 2)	ND	12.9 km 2 bore with 2 lanes per bore; forced air ventilation at three locations; 95% LD vehicles, 5% HD vehicles	[90]
Kaohsiung, Taiwan	NR NR NR	68.7 ± 19.6 † (tunnel 1) 147.4 ± 48.2 † (tunnel 2) 15.8 ± 7.2 † (tunnel 3)	ND	3 separate single bore tunnels with 2 lanes per bore; tunnel 1–0.4 km with 85% LD gasoline vehicles, tunnel 2–0.5 km with 52% motorcycles, tunnel 3–1.0 km with 35% LD gasoline and 39% motorcycles	[91]
Berkley, California	NR	23.8 (17.5–31.8)	ND	1.1 km 3 bore tunnel with 2 lanes per bore; exhaust ventilation; mostly LD gasoline powered vehicles	[92]
Seoul, Korea	15.3 ± 9.1	27.3 ± 13.3 ‡	ND	0.56 km two bore tunnel with 2 lanes per bore; natural ventilation via traffic flow;	[93]
Goteborg, Sweden	NR	46.7 ± 30.9 †	ND	0.45 km two bore tunnel with 3 lanes per bore; natural ventilation from traffic flow; 50% passenger cars with catalytic converters and 10% HD vehicles	[94]

* difference in inlet and outlet concentrations; † average concentration near the exit; ‡ average concentration at the center of tunnel; ≈ moderate traffic density; # summertime measurements; ^ emission factor units in mg/L; NR—not reported; ND—not determined; HD—heavy duty vehicles; LD—light duty vehicles; Sp—spring; Su—summer; Wi—winter.

**Table 4:** Comparison of the indoor/outdoor ratios of ETH from various studies *.

Location	Year	Sampling Sites	Indoor Conc. (ppbv)	Outdoor Conc. (ppbv)	I/O Ratio	Reference
Brownsville, Texas	1993	living room/outside residence	NR	NR	2.75	[191]
Athens, Greece	1994	research labs/building perimeter	13.0	6.6	1.97	[192]
Chicago, Illinois ^‡^	1994–1995	kitchens/local background sites	4.12	0.50	8.33	[186]
Windsor, Ontario ^†^	2005	living room/backyard	0.90	0.31	2.93	[187]
Regina, Saskatchewan ^†^	2007	living room/backyard	0.43	0.15	2.80	[177]
Halifax, Nova Scotia ^†^	2009	living room/backyard	1.72	0.21	8.13	[193]
Edmonton, Alberta ^†^	2010	living room/backyard	0.63	0.28	2.28	[194]
Al-Jahara, Kuwait	2010–2011	office area/near air supply unit	12.4	5.5	2.30	[195]
Beijing, China	2011–2012	living room/roof of the building	3.49	2.22	1.57	[196]
Atlanta, Georgia	NR	NR	0.50	0.17	2.94	[197]

* unless otherwise indicated samples were collected in the breathing zone of a non-smoking building and the results expressed as arithmetic means; ^†^ 24-h samples collected in the homes of non-smokers during the summer months; ^‡^ median values listed; NR—measured but not reported.

## Supplementary Materials

The following are available online at www.mdpi.com/1660-4601/15/1/66/s1, Table S1: Ambient air concentrations of propylene in Asia, Table S2: Ambient air concentrations of propylene in North America, Table S3: Ambient air concentrations of propylene in South America, Table S4: Ambient air concentrations of propylene in Europe, Table S5: Ambient air concentrations of propylene in marine areas, Table S6: Ambient air concentrations of propylene in other locations.

## References

[1] Wood Mackenzie (2014). Propylene Capacity Expected to Increase to 165 MT by 2030.

[2] CEH (2015). Chemical Economics Handbook. Propylene.

[3] ACC Propylene. Product Stewardship Guidance Manual. http://www2.spotchemi.eu/techspec/03426.pdf.

[4] Sanderson M.G. Emission of Isoprene, Monoterpenes, Ethene and Propene by Vegetation. ftp://149.222-62-69.ftth.swbr.surewest.net/TreePDF/HCTN_40.pdf.

[5] Health Canada (2014). Screening Assessment for 1-Propene.

[6] HSDB (2006). Propylene CASRN 115-07-1. 175.

[7] OEHHA (2015). Air Toxics Hot Spots Program. Guidance Manual for Preparation of Health Risk Assessments.

[8] SCAPA Protective Action Criteria (PAC) Rev. 29 Based on Applicable 60-Minute AEGLs, ERPGs, or TEELs (Table 4), 2016. https://sp.eota.energy.gov/pac/docs/Revision_29_Table4.pdf.

[9] ACGIH (2016). Threshold Limit Values for Chemical Substances and Physical Agents.

[10] Trentmann J., Andreae M.O., Graf H.F. (2003). Chemical Processes in a Young Biomass-Burning Plume. J. Geophys. Res. Atmos..

[11] Lawrimore J.H., Das M., Aneja V.P. (1995). Vertical Sampling and Analysis of Nonmethane Hydrocarbons for Ozone Control in Urban North Carolina. J. Geophys. Res. Atmos..

[12] Tille K.J.W., Savelsberg M., Bachmann K. (1985). Airborne Measurements of Nonmethane Hydrocarbons over Western Europe—Vertical Distributions, Seasonal Cycles of Mixing Ratios and Source Strengths. Atmos. Environ..

[13] Matsunaga S.N., Chatani S., Morikawa T., Nakatsuka S., Suthawaree J., Tajima Y., Kato S., Kajii Y., Minoura H. (2010). Evaluation of Non-Methane Hydrocarbon (NMHC) Emissions Based on an Ambient Air Measurement in Tokyo Area, Japan. Atmos. Environ..

[14] Li L.F., Wang X.M. (2012). Seasonal and Diurnal Variations of Atmospheric Non-Methane Hydrocarbons in Guangzhou, China. Int. J. Environ. Res. Public Health.

[15] Na K., Kim Y.P., Moon K.C. (2003). Diurnal Characteristics of Volatile Organic Compounds in the Seoul Atmosphere. Atmos. Environ..

[16] Qin Y., Walk T., Gary R., Yao X., Elles S. (2007). C_2_–C_10_ Nonmethane Hydrocarbons Measured in Dallas, USA—Seasonal Trends and Diurnal Characteristics. Atmos. Environ..

[17] Fanizza C., Incoronato F., Baiguera S., Schiro R., Brocco D. (2014). Volatile Organic Compound Levels at One Site in Rome Urban Air. Atmos. Pollut. Res..

[18] Roemer M., Builtjes P., Esser P., Guicherit R., Thijsse T. (1999). C_2_–C_5_ Hydrocarbon Measurements in The Netherlands 1981–1991. Atmos. Environ..

[19] Sahu L.K., Lal S. (2006). Characterization of C_2_–C_4_ NMHCs Distributions at a High Altitude Tropical Site in India. J. Atmos. Chem..

[20] Penkett S.A., Burgess R.A., Coe H., Coll I., Hov O., Lindskog A., Schmidbauer N., Solberg S., Roemer M., Thijsse T. (2007). Evidence for Large Average Concentrations of the Nitrate Radical (NO_3_) in Western Europe from the HANSA Hydrocarbon Database. Atmos. Environ..

[21] Navazo M., Durana N., Alonso L., Gomez M.C., Garcia J.A., Ilardia J.L., Gangoiti G., Iza J. (2008). High Temporal Resolution Measurements of Ozone Precursors in a Rural Background Station. A Two-Year Study. Environ. Monit. Assess..

[22] Gnauk T., Rolle W. (1998). A Three-Year Study of Nonmethane Hydrocarbons in Surface Air over Saxony (Germany). J. Atmos. Chem..

[23] Myers J.L., Phillips T., Grant R.L. (2015). Emissions and Ambient Air Monitoring Trends of Lower Olefins across Texas from 2002 to 2012. Chem. Biol. Interact..

[24] Von Schneidemesser E., Monks P.S., Plass-Duelmer C. (2010). Global Comparison of VOC and CO Observations in Urban Areas. Atmos. Environ..

[25] Dollard G., Dumitrean P., Telling S., Dixon J., Derwent R. (2007). Observed Trends in Ambient Concentrations of C_2_–C_8_ Hydrocarbons in the United Kingdom over the Period from 1993 to 2004. Atmos. Environ..

[26] Pang Y.B., Fuentes M., Rieger P. (2015). Trends in Selected Ambient Volatile Organic Compound (VOC) Concentrations and a Comparison to Mobile Source Emission Trends in California’s South Coast Air Basin. Atmos. Environ..

[27] Warneke C., de Gouw J.A., Holloway J.S., Peischl J., Ryerson T.B., Atlas E., Blake D., Trainer M., Parrish D.D. (2012). Multiyear Trends in Volatile Organic Compounds in Los Angeles, California: Five Decades of Decreasing Emissions. J. Geophys. Res. Atmos..

[28] Wang M., Shao M., Chen W., Lu S., Liu Y., Yuan B., Zhang Q., Zhang Q., Chang C.C., Wang B. (2015). Trends of Non-Methane Hydrocarbons (NMHC) Emissions in Beijing During 2002–2013. Atmos. Chem. Phys..

[29] Poisson N., Kanakidou M., Crutzen P.J. (2000). Impact of Non-Methane Hydrocarbons on Tropospheric Chemistry and the Oxidizing Power of the Global Troposphere: 3-Dimensional Modelling Results. J. Atmos. Chem..

[30] Ito A., Sillman S., Penner J.E. (2009). Global Chemical Transport Model Study of Ozone Response to Changes in Chemical Kinetics and Biogenic Volatile Organic Compounds Emissions Due to Increasing Temperatures: Sensitivities to Isoprene Nitrate Chemistry and Grid Resolution. J. Geophys. Res. Atmos..

[31] Pozzer A., Jockel P., Tost H., Sander R., Ganzeveld L., Kerkweg A., Lelieveld J. (2007). Simulating Organic Species with the Global Atmospheric Chemistry General Circulation Model ECHAM5/MESSy1: A Comparison of Model Results with Observations. Atmos. Chem. Phys..

[32] Williams J.E., van Velthoven P.F.J., Brenninkmeijer C.A.M. (2013). Quantifying the Uncertainty in Simulating Global Tropospheric Composition Due to the Variability in Global Emission Estimates of Biogenic Volatile Organic Compounds. Atmos. Chem. Phys..

[33] Kaiser J.W., Heil A., Andreae M.O., Benedetti A., Chubarova N., Jones L., Morcrette J.J., Razinger M., Schultz M.G., Suttie M. (2012). Biomass Burning Emissions Estimated with a Global Fire Assimilation System Based on Observed Fire Radiative Power. Biogeosciences.

[34] Olivier J., Peters J., Granier C., OPetron G., Muller J.F., Wallens S. Present and Future Surface Emissions of Atmospheric Compounds, 2003. http://tropo.aeronomie.be/pdf/POET_emissions_report.pdf.

[35] Von Kuhlmann R., Lawrence M.G., Crutzen P.J., Rasch P.J. (2003). A Model for Studies of Tropospheric Ozone and Nonmethane Hydrocarbons: Model Description and Ozone Results. J. Geophys. Res. Atmos..

[36] Horowitz L.W., Walters S., Mauzerall D.L., Emmons L.K., Rasch P.J., Granier C., Tie X.X., Lamarque J.F., Schultz M.G., Tyndall G.S. (2003). A Global Simulation of Tropospheric Ozone and Related Tracers: Description and Evaluation of MOZART, Version 2. J. Geophys. Res. Atmos..

[37] Folberth G.A., Hauglustaine D.A., Lathiere J., Brocheton F. (2006). Interactive Chemistry in the Laboratoire de Meteorologie Dynamique General Circulation Model: Model Description and Impact Analysis of Biogenic Hydrocarbons on Tropospheric Chemistry. Atmos. Chem. Phys..

[38] Lewis A.C., Evans M.J., Hopkins J.R., Punjabi S., Read K.A., Purvis R.M., Andrews S.J., Moller S.J., Carpenter L.J., Lee J.D. (2013). The Influence of Biomass Burning on the Global Distribution of Selected Non-Methane Organic Compounds. Atmos. Chem. Phys..

[39] Wang B.L., Liu Y., Shao M., Lu S.H., Wang M., Yuan B., Gong Z.H., He L.Y., Zeng L.M., Hu M. (2016). The Contributions of Biomass Burning to Primary and Secondary Organics: A Case Study in Pearl River Delta (PRD), China. Sci. Total Environ..

[40] Brunke E.G., Labuschagne C., Scheel H.E. (2001). Trace Gas Variations at Cape Point, South Africa, during May 1997 Following a Regional Biomass Burning Episode. Atmos. Environ..

[41] Hobbs P.V., Sinha P., Yokelson R.J., Christian T.J., Blake D.R., Gao S., Kirchstetter T.W., Novakov T., Pilewskie P. (2003). Evolution of Gases and Particles from a Savanna Fire in South Africa. J. Geophys. Res. Atmos..

[42] Schultz M.G., Heil A., Hoelzemann J.J., Spessa A., Thonicke K., Goldammer J.G., Held A.C., Pereira J.M.C., van het Bolscher M. (2008). Global Wildland Fire Emissions from 1960 to 2000. Glob. Biogeochem. Cycles.

[43] Yonemura S., Sudo S., Tsuruta H., Kawashima S. (2002). Relations between Light Hydrocarbon, Carbon Monoxide, and Carbon Dioxide Concentrations in the Plume from the Combustion of Plant Material in a Furnace. J. Atmos. Chem..

[44] Barrefors G., Petersson G. (1995). Assessment by Gas-Chromatography and Gas-Chromatography Mass-Spectrometry of Volatile Hydrocarbons from Biomass Burning. J. Chromatogr. A.

[45] Jost C., Trentmann J., Sprung D., Andreae M.O., McQuaid J.B., Barjat H. (2003). Trace Gas Chemistry in a Young Biomass Burning Plume Over Namibia: Observations and Model Simulations. J. Geophys. Res. Atmos..

[46] Shi J.W., Deng H., Bai Z.P., Kong S.F., Wang X.Y., Hao J.M., Han X.Y., Ning P. (2015). Emission and Profile Characteristic of Volatile Organic Compounds Emitted from Coke Production, Iron Smelt, Heating Station and Power Plant in Liaoning Province, China. Sci. Total Environ..

[47] Laursen K.K., Hobbs P.V., Radke L.F., Rasmussen R.A. (1992). Some Trace Gas Emissions from North American Biomass Fires with an Assessment of Regional and Global Fluxes from Biomass Burning. J. Geophys. Res. Atmos..

[48] Aurell J., Gullett B.K., Tabor D. (2015). Emissions from Southeastern US Grasslands and Pine Savannas: Comparison of Aerial and Ground Field Measurements with Laboratory Burns. Atmos. Environ..

[49] Guild L.S., Kauffman J.B., Cohen W.B., Hlavka C.A., Ward D.E. (2004). Modeling Biomass Burning Emissions for Amazon Forest and Pastures in Rondonia, Brazil. Ecol. Appl..

[50] Akagi S.K., Yokelson R.J., Wiedinmyer C., Alvarado M.J., Reid J.S., Karl T., Crounse J.D., Wennberg P.O. (2011). Emission Factors for Open and Domestic Biomass Burning for Use in Atmospheric Models. Atmos. Chem. Phys..

[51] Yokelson R.J., Burling I.R., Gilman J.B., Warneke C., Stockwell C.E., de Gouw J., Akagi S.K., Urbanski S.P., Veres P., Roberts J.M. (2013). Coupling Field and Laboratory Measurements to Estimate the Emission Factors of Identified and Unidentified Trace Gases for Prescribed Fires. Atmos. Chem. Phys..

[52] Yokelson R.J., Christian T.J., Karl T.G., Guenther A. (2008). The Tropical Forest and Fire Emissions Experiment: Laboratory Fire Measurements and Synthesis of Campaign Data. Atmos. Chem. Phys..

[53] Stockwell C.E., Yokelson R.J., Kreidenweis S.M., Robinson A.L., Demott P.J., Sullivan R.C., Reardon J., Ryan K.C., Griffith D.W.T., Stevens L. (2014). Trace Gas Emissions from Combustion of Peat, Crop Residue, Domestic Biofuels, Grasses, and Other Fuels: Configuration and Fourier Transform Infrared (FTIR) Component of the Fourth Fire Lab at Missoula Experiment (FLAME-4). Atmos. Chem. Phys..

[54] George I.J., Black R.R., Geron C.D., Aurell J., Hays M.D., Preston W.T., Gullett B.K. (2016). Volatile and Semivolatile Organic Compounds in Laboratory Peat Fire Emissions. Atmos. Environ..

[55] Christian T.J., Kleiss B., Yokelson R.J., Holzinger R., Crutzen P.J., Hao W.M., Saharjo B.H., Ward D.E. (2003). Comprehensive Laboratory Measurements of Biomass-Burning Emissions: 1. Emissions from Indonesian, African, and Other Fuels. J. Geophys. Res. Atmos..

[56] Stockwell C.E., Jayarathne T., Cochrane M.A., Ryan K.C., Putra E.I., Saharjo B.H., Nurhayati A.D., Albar I., Blake D.R., Simpson I.J. (2016). Field Measurements of Trace Gases and Aerosols Emitted by Peat Fires in Central Kalimantan, Indonesia, during the 2015 El Nino. Atmos. Chem. Phys..

[57] McMeeking G.R., Kreidenweis S.M., Baker S., Carrico C.M., Chow J.C., Collett J.L., Hao W.M., Holden A.S., Kirchstetter T.W., Malm W.C. (2009). Emissions of Trace Gases and Aerosols during the Open Combustion of Biomass in the Laboratory. J. Geophys. Res. Atmos..

[58] Burling I.R., Yokelson R.J., Griffith D.W.T., Johnson T.J., Veres P., Roberts J.M., Warneke C., Urbanski S.P., Reardon J., Weise D.R. (2010). Laboratory Measurements of Trace Gas Emissions from Biomass Burning of Fuel Types from the Southeastern and Southwestern United States. Atmos. Chem. Phys..

[59] Zhang Y.S., Shao M., Lin Y., Luan S.J., Mao N., Chen W.T., Wang M. (2013). Emission Inventory of Carbonaceous Pollutants from Biomass Burning in the Pearl River Delta Region, China. Atmos. Environ..

[60] Li X.H., Wang S.X., Duan L., Hao J.M. (2009). Characterization of Non-Methane Hydrocarbons Emitted from Open Burning of Wheat Straw and Corn Stover in China. Environ. Res. Lett..

[61] Cooper D.A., Peterson K., Simpson D. (1996). Hydrocarbon, PAH and PCB Emissions from Ferries: A Case Study in the Skagerak-Kattegatt-Oresund Region. Atmos. Environ..

[62] Henry R.F. (2013). Weekday/Weekend Differences in Gasoline Related Hydrocarbons at Coastal PAMS Sites Due to Recreational Boating. Atmos. Environ..

[63] Beyersdorf A.J., Thornhill K.L., Winstead E.L., Ziemba L.D., Blake D.R., Timko M.T., Anderson B.E. (2012). Power-Dependent Speciation of Volatile Organic Compounds in Aircraft Exhaust. Atmos. Environ..

[64] Ho K.F., Ho S.S.H., Lee S.C., Louie P.K.K., Cao J.J., Deng W.J. (2013). Volatile Organic Compounds in Roadside Environment of Hong Kong. Aerosol Air Qual. Res..

[65] Wong Y.C., Sin D.W.M., Yeung L.L. (2002). Assessment of the Air Quality in Indoor Car Parks. Indoor Built Environ..

[66] Wu B.-Z., Chang C.C., Sree U., Chiu K.-H., Lo J.-G. (2006). Measurement of Non-Methane Hydrocarbons in Taipei City and Their Impact on Ozone Formation in Relation to Air Quality. Anal. Chim. Acta.

[67] Broderick B.M., Marnane I.S. (2002). A Comparison of the C_2_–C_9_ Hydrocarbon Compositions of Vehicle Fuels and Urban Air in Dublin, Ireland. Atmos. Environ..

[68] Olson D.A., Hammond D.M., Seila R.L., Burke J.M., Norris G.A. (2009). Spatial Gradients and Source Apportionment of Volatile Organic Compounds Near Roadways. Atmos. Environ..

[69] O’Donoghue R.T., Broderick B.M. (2009). Local and Regional Sources of C_2_–C_8_ Hydrocarbon Concentrations at a Sub-Urban Motorway Site (M4) in Ireland. Environ. Monit. Assess..

[70] Rubin J.I., Kean A.J., Harley R.A., Millet D.B., Goldstein A.H. (2006). Temperature Dependence of Volatile Organic Compound Evaporative Emissions from Motor Vehicles. J. Geophys. Res. Atmos..

[71] Nelson P.F., Quigley S.M. (1984). The Hydrocarbon Composition of Exhaust Emitted from Gasoline Fueled Vehicles. Atmos. Environ..

[72] Montero L., Duane M., Manfredi U., Astorga C., Martini G., Carriero M., Krasenbrink A., Larsen B.R. (2010). Hydrocarbon Emission Fingerprints from Contemporary Vehicle/Engine Technologies with Conventional and New Fuels. Atmos. Environ..

[73] Wang J., Jin L.M., Gao J.H., Shi J.W., Zhao Y.L., Liu S.X., Jin T.S., Bai Z.P., Wu C.Y. (2013). Investigation of Speciated VOC in Gasoline Vehicular Exhaust Under ECE and EUDC Test Cycles. Sci. Total Environ..

[74] Yao Z.L., Shen X.B., Ye Y., Cao X.Y., Jiang X., Zhang Y.Z., He K.B. (2015). On-Road Emission Characteristics of VOCs from Diesel Trucks in Beijing, China. Atmos. Environ..

[75] Pang Y.B., Fuentes M., Rieger P. (2014). Trends in the Emissions of Volatile Organic Compounds (VOCs) from Light-Duty Gasoline Vehicles Tested on Chassis Dynamometers in Southern California. Atmos. Environ..

[76] Stump F.D., Knapp K.T., Ray W.D. (1990). Seasonal Impact of Blending Oxygenated Organics with Gasoline on Motor-Vehicle Tailpipe and Evaporative Emissions. J. Air Waste Manag. Assoc..

[77] Lough G.C., Schauer J.J., Lonneman W.A., Allen M.K. (2005). Summer and Winter Nonmethane Hydrocarbon Emissions from on-Road Motor Vehicles in the Midwestern United States. J. Air Waste Manag. Assoc..

[78] Chen T.Y., Simpson I.J., Blake D.R., Rowland F.S. (2001). Impact of the Leakage of Liquefied Petroleum Gas (LPG) on Santiago Air Quality. Geophys. Res. Lett..

[79] Blake D.R., Rowland F.S. (1995). Urban Leakage of Liquefied Petroleum Gas and Its Impact on Mexico City Air Quality. Science.

[80] Hwa M.Y., Hsieh C.C., Wu T.C., Chang L.F.W. (2002). Real-World Vehicle Emissions and VOCs Profile in the Taipei Tunnel Located at Taiwan Taipei Area. Atmos. Environ..

[81] Staehelin J., Keller C., Stahel W., Schlapfer K., Wunderli S. (1998). Emission Factors from Road Traffic from a Tunnel Study (Gubrist Tunnel, Switzerland). Part III: Results of Organic Compounds, SO_2_ and Speciation of Organic Exhaust Emission. Atmos. Environ..

[82] Ho K.F., Lee S.C., Ho W.K., Blake D.R., Cheng Y., Li Y.S., Ho S.S.H., Fung K., Louie P.K.K., Park D. (2009). Vehicular Emission of Volatile Organic Compounds (VOCs) from a Tunnel Study in Hong Kong. Atmos. Chem. Phys..

[83] Araizaga A.E., Mancilla Y., Mendoza A. (2013). Volatile Organic Compound Emissions from Light-Duty Vehicles in Monterrey, Mexico: A Tunnel Study. Int. J. Environ. Res..

[84] Touaty M., Bonsang B. (2000). Hydrocarbon Emissions in a Highway Tunnel in the Paris Area. Atmos. Environ..

[85] Sagebiel J.C., Zielinska B., Pierson W.R., Gertler A.W. (1996). Real-World Emissions and Calculated Reactivities of Organic Species from Motor Vehicles. Atmos. Environ..

[86] Gentner D.R., Worton D.R., Isaacman G., Davis L.C., Dallmann T.R., Wood E.C., Herndon S.C., Goldstein A.H., Harley R.A. (2013). Chemical Composition of Gas-Phase Organic Carbon Emissions from Motor Vehicles and Implications for Ozone Production. Environ. Sci. Technol..

[87] Chiang H.L., Hwu C.S., Chen S.Y., Wu M.C., Ma S.Y., Huang Y.S. (2007). Emission Factors and Characteristics of Criteria Pollutants and Volatile Organic Compounds (VOCs) in a Freeway Tunnel Study. Sci. Total Environ..

[88] Duffy B.L., Nelson P.F. (1996). Non-Methane Exhaust Composition in the Sydney Harbour Tunnel: A Focus on Benzene and 1,3-Butadiene. Atmos. Environ..

[89] Na K., Kim Y.P., Moon K.C. (2002). Seasonal Variation of the C_2_–C_9_ Hydrocarbons Concentrations and Compositions Emitted from Motor Vehicles in a Seoul Tunnel. Atmos. Environ..

[90] Lai C.H., Peng Y.P. (2012). Volatile Hydrocarbon Emissions from Vehicles and Vertical Ventilations in the Hsuehshan Traffic Tunnel, Taiwan. Environ. Monit. Assess..

[91] Chen K.S., Lai C.H., Ho Y.T. (2003). Source Profiles and Ozone Formation Potentials of Volatile Organic Compounds in Three Traffic Tunnels in Kaohsiung, Taiwan. J. Air Waste Manag. Assoc..

[92] Zielinska B., Fung K.K. (1994). The Composition and Concentration of Hydrocarbons in the Range of C_2_ to C_18_ Emitted from Motor-Vehicles. Sci. Total Environ..

[93] Na K.S. (2006). Determination of VOC Source Signature of Vehicle Exhaust in a Traffic Tunnel. J. Environ. Manag..

[94] Barrefors G., Petersson G. (1992). Volatile Hazardous Hydrocarbons in a Scandinavian Urban Road Tunnel. Chemosphere.

[95] Siegl W.O., Mccabe R.W., Chun W., Kaiser E.W., Perry J., Henig Y.I., Trinker F.H., Anderson R.W. (1992). Speciated Hydrocarbon Emissions from the Combustion of Single Component Fuels. 1. Effect of Fuel Structure. J. Air Waste Manag. Assoc..

[96] Duffy B.L., Nelson P.F., Ye Y., Weeks I.A. (1999). Speciated Hydrocarbon Profiles and Calculated Reactivities of Exhaust and Evaporative Emissions from 82 in-Use Light-Duty Australian Vehicles. Atmos. Environ..

[97] Siegl W.O., Hammerle R.H., Herrmann H.M., Wenclawiak B.W., Luers-Jongen B. (1999). Organic Emissions Profile for a Light-Duty Diesel Vehicle. Atmos. Environ..

[98] Tsai J.H., Huang P.H., Chiang H.L. (2014). Characteristics of Volatile Organic Compounds from Motorcycle Exhaust Emission during Real-World Driving. Atmos. Environ..

[99] Guo H., Zou S.C., Tsai W.Y., Chan L.Y., Blake D.R. (2011). Emission Characteristics of Nonmethane Hydrocarbons from Private Cars and Taxis at Different Driving Speeds in Hong Kong. Atmos. Environ..

[100] Adam T.W., Astorga C., Clairotte M., Duane M., Elsasser M., Krasenbrink A., Larsen B.R., Manfredi U., Martini G., Montero L. (2011). Chemical Analysis and Ozone Formation Potential of Exhaust from Dual-Fuel (Liquefied Petroleum Gas/Gasoline) Light Duty Vehicles. Atmos. Environ..

[101] Lyu X.P., Guo H., Simpson I.J., Meinardi S., Louie P.K.K., Ling Z.H., Wang Y., Liu M., Luk C.W.Y., Wang N. (2016). Effectiveness of Replacing Catalytic Converters in LPG-Fueled Vehicles in Hong Kong. Atmos. Chem. Phys..

[102] Ludykar D., Westerholm R., Almen J. (1999). Cold Start Emissions at +22, −7 and −20 °C Ambient Temperatures from a Three-Way Catalyst (TWC) Car: Regulated and Unregulated Exhaust Components. Sci. Total Environ..

[103] Schauer J.J., Kleeman M.J., Cass G.R., Simoneit B.R.T. (1999). Measurement of Emissions from Air Pollution Sources. 2. C_1_ through C_30_ Organic Compounds from Medium Duty Diesel Trucks. Environ. Sci. Technol..

[104] Schauer J.J., Kleeman M.J., Cass G.R., Simoneit B.R.T. (2002). Measurement of Emissions from Air Pollution Sources. 5. C_1_–C_32_ Organic Compounds from Gasoline-Powered Motor Vehicles. Environ. Sci. Technol..

[105] Derwent R.G., Stevenson D.S., Collins W.J., Johnson C.E. (2004). Intercontinental Transport and the Origins of the Ozone Observed at Surface Sites in Europe. Atmos. Environ..

[106] Goldstein A.H., Fan S.M., Goulden M.L., Munger J.W., Wofsy S.C. (1996). Emissions of Ethene, Propene, and 1-Butene by a Midlatitude Forest. J. Geophys. Res. Atmos..

[107] Janson R., De Serves C. (1998). Isoprene Emissions from Boreal Wetlands in Scandinavia. J. Geophys. Res. Atmos..

[108] Haapanala S., Rinne J., Pystynen K.H., Hellen H., Hakola H., Riutta T. (2006). Measurements of Hydrocarbon Emissions from a Boreal Fen Using the REA Technique. Biogeosciences.

[109] Hellen H., Hakola H., Pystynen K.H., Rinne J., Haapanala S. (2006). C_2_–C_10_ Hydrocarbon Emissions from a Boreal Wetland and Forest Floor. Biogeosciences.

[110] Jones C.E., Hopkins J.R., Lewis A.C. (2011). In Situ Measurements of Isoprene and Monoterpenes within a South-East Asian Tropical Rainforest. Atmos. Chem. Phys..

[111] Hakola H., Laurila T., Lindfors V., Hellen H., Gaman A., Rinne J. (2001). Variation of the VOC Emission Rates of Birch Species during the Growing Season. Boreal Environ. Res..

[112] Hakola H., Rinne J., Laurila T. (1998). The Hydrocarbon Emission Rates of Tea-Leafed Willow (*Salix phylicifolia*), Silver Birch (*Betula pendula*) and European Aspen (*Populus tremula*). Atmos. Environ..

[113] Isidorov V.A., Zenkevich I.G., Ioffe B.V. (1985). Volatile Organic-Compounds in the Atmosphere of Forests. Atmos. Environ..

[114] Svedberg U., Samuelsson J., Melin S. (2008). Hazardous Off-Gassing of Carbon Monoxide and Oxygen Depletion during Ocean Transportation of Wood Pellets. Ann. Occup. Hyg..

[115] Kang D., Aneja V.P., Das M., Seila R. (2004). Measurements of Air-Surface Exchange Rates of Volatile Organic Compounds. Int. J. Environ. Pollut..

[116] Smiatek G., Steinbrecher R. (2006). Temporal and Spatial Variation of Forest VOC Emissions in Germany in the Decade 1994–2003. Atmos. Environ..

[117] Lewis A.C., McQuaid J.B., Carslaw N., Pilling M.J. (1999). Diurnal Cycles of Short-Lived Tropospheric Alkenes at a North Atlantic Coastal Site. Atmos. Environ..

[118] Plass-Dulmer C., Koppmann R., Ratte M., Rudolph J. (1995). Light Nonmethane Hydrocarbons in Seawater. Glob. Biogeochem. Cycles.

[119] Tran S., Bonsang B., Gros V., Peeken I., Sarda-Esteve R., Bernhardt A., Belviso S. (2013). A Survey of Carbon Monoxide and Non-Methane Hydrocarbons in the Arctic Ocean during Summer 2010. Biogeosciences.

[120] Plass C., Koppmann R., Rudolph J. (1992). Light Hydrocarbons in the Surface Water of the Mid-Atlantic. J. Atmos. Chem..

[121] Broadgate W.J., Liss P.S., Penkett S.A. (1997). Seasonal Emissions of Isoprene and Other Reactive Hydrocarbon Gases from the Ocean. Geophys. Res. Lett..

[122] Bonsang B., Al Aarbaoui A., Sciare J. (2008). Diurnal Variation of Non-Methane Hydrocarbons in the Subantarctic Atmosphere. Environ. Chem..

[123] Lamontagne R.A., Swinnerton J.W., Linnenbom V.J. (1974). C_1_–C_4_ Hydrocarbons in North and South Pacific. Tellus.

[124] Gist N., Lewis A.C. (2006). Seasonal Variations of Dissolved Alkenes in Coastal Waters. Mar. Chem..

[125] Ratte M., Plassdulmer C., Koppmann R., Rudolph J. (1995). Horizontal and Vertical Profiles of Light-Hydrocarbons in Sea-Water Related to Biological, Chemical and Physical Parameters. Tellus B Chem. Phys. Meteorol..

[126] Ratte M., Bujok O., Spitzy A., Rudolph J. (1998). Photochemical Alkene Formation in Seawater from Dissolved Organic Carbon: Results from Laboratory Experiments. J. Geophys. Res. Atmos..

[127] Ratte M., Plassdulmer C., Koppmann R., Rudolph J., Denga J. (1993). Production Mechanism of C_2_–C_4_ Hydrocarbons in Seawater—Field Measurements and Experiments. Glob. Biogeochem. Cycles.

[128] Riemer D.D., Milne P.J., Zika R.G., Pos W.H. (2000). Photoproduction of Nonmethane Hydrocarbons (NMHCs) in Seawater. Mar. Chem..

[129] Claypool G.E., Kvenvolden K.A. (1983). Methane and Other Hydrocarbon Gases in Marine Sediment. Annu. Rev. Earth Planet. Sci..

[130] Kvenvolden K.A. (1988). Hydrocarbon Gas in Sediment of the Southern Pacific Ocean. Geo-Mar. Lett..

[131] Kvenvolden K.A., Redden G.D. (1980). Hydrocarbon-Gas in Sediment from the Shelf, Slope, and Basin of the Bering Sea. Geochim. Cosmochim. Acta.

[132] Kvenvolden K.A., Frank T.J., Golan-Bac M. (1990). Hydrocarbon gases in tertiary and quartenary sediments offshore Peru—Results and comparisons. Proc. Ocean Drill. Program Sci. Results.

[133] Kvenvolden K.A., Golan-Bac M., McDonald T.J., Pflaum R.C., Brooks J.M. (1989). Hydrocarbon Gases in Sediment of the Voring Plateau, Norwegian Sea. Proc. Ocean Drill. Program Sci. Results.

[134] Bernard B.B., Brooks J.M., Sackett W.M. (1978). Light Hydrocarbons in Recent Texas Continental Shelf and Slope Sediments. J. Geophys. Res. Oceans.

[135] Vogel T.M., Oremland R.S., Kvenvolden K.A. (1982). Low-Temperature Formation of Hydrocarbon Gases in San Francisco Bay Sediment (California, USA). Chem. Geol..

[136] Bottenheim J.W., Boudries H., Brickell P.C., Atlas E. (2002). Alkenes in the Arctic Boundary Layer at Alert, Nunavut, Canada. Atmos. Environ..

[137] Swanson A.L., Blake N.J., Dibb J.E., Albert M.R., Blake D.R., Rowland F.S. (2002). Photochemically Induced Production of CH_3_Br, CH_3_I, C_2_H_5_I, Ethene, and Propene within Surface Snow at Summit, Greenland. Atmos. Environ..

[138] Helmig D., Stephens C.R., Caramore J., Hueber J. (2014). Seasonal Behavior of Non-Methane Hydrocarbons in the Firn Air at Summit, Greenland. Atmos. Environ..

[139] McKay W.A., Turner M.F., Jones B.M.R., Halliwell C.M. (1996). Emissions of Hydrocarbons from Marine Phytoplankton—Some Results from Controlled Laboratory Experiments. Atmos. Environ..

[140] Shaw S.L. (2001). The Production of Non-Methane Hydrocarbons by Marine Plankton. Ph.D. Thesis.

[141] Broadgate W.J., Malin G., Kupper F.C., Thompson A., Liss P.S. (2004). Isoprene and Other Non-Methane Hydrocarbons from Seaweeds: A Source of Reactive Hydrocarbons to the Atmosphere. Mar. Chem..

[142] Derendorp L., Holzinger R., Wishkerman A., Keppler F., Rockmann T. (2011). Methyl Chloride and C_2_–C_5_ Hydrocarbon Emissions from Dry Leaf Litter and Their Dependence on Temperature. Atmos. Environ..

[143] Derendorp L., Holzinger R., Rockmann T. (2011). UV-Induced Emissions of C_2_–C_5_ Hydrocarbons from Leaf Litter. Environ. Chem..

[144] Redeker K.R., Meinardi S., Blake D., Sass R. (2003). Gaseous Emissions from Flooded Rice Paddy Agriculture. J. Geophys. Res. Atmos..

[145] Wang R., Wu T., Dai W.H., Liu H., Zhao J., Wang X.M., Huang F.Y., Wang Z., Shi C.F. (2015). Effects of Straw Return on C_2_–C_5_ Non-Methane Hydrocarbon (NMHC) Emissions from Agricultural Soils. Atmos. Environ..

[146] Zhao J., Wang Z., Wu T., Wang X.M., Dai W.H., Zhang Y.J., Wang R., Zhang Y.G., Shi C.F. (2016). Volatile Organic Compound Emissions from Straw-Amended Agricultural Soils and Their Relations to Bacterial Communities: A Laboratory Study. J. Environ. Sci..

[147] Hodges C.F., Campbell D.A. (1998). Gaseous Hydrocarbons Associated with Black Layer Induced by the Interaction of Cyanobacteria and Desulfovibrio Desulfuricans. Plant Soil.

[148] Van Ginkel C.G., Welten H.G.J., Debont J.A.M. (1987). Oxidation of Gaseous and Volatile Hydrocarbons by Selected Alkene-Utilizing Bacteria. Appl. Environ. Microbiol..

[149] Fletcher K.E., Loffler F.E., Richnow H.H., Nijenhuis I. (2009). Stable Carbon Isotope Fractionation of 1,2-Dichloropropane During Dichloroelimination by Dehalococcoides Populations. Environ. Sci. Technol..

[150] Maness A.D., Bowman K.S., Yan J., Rainey F.A., Moe W.M. (2012). *Dehalogenimonas* spp. Can Reductively Dehalogenate High Concentrations of 1,2-Dichloroethane, 1,2-Dichloropropane, and 1,1,2-Trichloroethane. AMB Express.

[151] McCoy B.J., Fischbeck P.S., Gerard D. (2010). How Big Is Big? How Often Is Often? Characterizing Texas Petroleum Refining Upset Air Emissions. Atmos. Environ..

[152] Allen D.T., Torres V.M. (2011). TCEQ 2010 Flare Study.

[153] Wood E.C., Herndon S.C., Fortner E.C., Onasch T.B., Wormhoudt J., Kolb C.E., Knighton W.B., Lee B.H., Zavala M., Molina L. (2012). Combustion and Destruction/Removal Efficiencies of In-Use Chemical Flares in the Greater Houston Area. Ind. Eng. Chem. Res..

[154] Al-Fadhli F.M., Kimura Y., McDonald-Buller E.C., Allen D.T. (2012). Impact of Flare Destruction Efficiency and Products of Incomplete Combustion on Ozone Formation in Houston, Texas. Ind. Eng. Chem. Res..

[155] Yen C.H., Horng J.J. (2009). Volatile Organic Compounds (VOCs) Emission Characteristics and Control Strategies for a Petrochemical Industrial Area in Middle Taiwan. J. Environ. Sci. Health A.

[156] Mellqvist J., Samuelsson J., Johansson J., Rivera C., Lefer B., Alvarez S., Jolly J. (2010). Measurements of Industrial Emissions of Alkenes in Texas Using the Solar Occultation Flux Method. J. Geophys. Res. Atmos..

[157] Murphy C.F., Allen D.T. (2005). Hydrocarbon Emissions from Industrial Release Events in the Houston-Galveston Area and Their Impact on Ozone Formation. Atmos. Environ..

[158] Olaguer E.P. (2012). Near-Source Air Quality Impacts of Large Olefin Flares. J. Air Waste Manag. Assoc..

[159] USEPA (2017). Toxic Release Inventory Pollution Prevention Search. Propylene 115-07-1.

[160] Washenfelder R.A., Trainer M., Frost G.J., Ryerson T.B., Atlas E.L., de Gouw J.A., Flocke F.M., Fried A., Holloway J.S., Parrish D.D. (2010). Characterization of NO_x_, SO_2_, Ethene, and Propene from Industrial Emission Sources in Houston, Texas. J. Geophys. Res. Atmos..

[161] Modrak M., Jozewicz W., Swett G. (2009). Application of Optical Remote Sensing in the Petroleum and Petrochemical Industries.

[162] Mo Z.W., Shao M., Lu S.H., Qu H., Zhou M.Y., Sun J., Gou B. (2015). Process-Specific Emission Characteristics of Volatile Organic Compounds (VOCs) from Petrochemical Facilities in the Yangtze River Delta, China. Sci. Total Environ..

[163] Harrison R.M., Holman C.D. (1980). Some Measurements of Low Molecular Weight Hydrocarbons in an Area with Petrochemical Industrialization. Environ. Technol. Lett..

[164] Sather M.E., Cavender K. (2012). Update of Long-Term Trends Analysis of Ambient 8-Hour Ozone and Precursor Monitoring Data in the South Central U.S.; Encouraging News. J. Environ. Monit..

[165] Yokelson R.J., Burling I.R., Urbanski S.P., Atlas E.L., Adachi K., Buseck P.R., Wiedinmyer C., Akagi S.K., Toohey D.W., Wold C.E. (2011). Trace Gas and Particle Emissions from Open Biomass Burning in Mexico. Atmos. Chem. Phys..

[166] Molto J., Font R., Galvez A., Rey M.D., Pequenin A. (2010). Analysis of Dioxin-Like Compounds Formed in the Combustion of Tomato Plant. Chemosphere.

[167] Barlow A., Contos D.A., Holdren M.W., Garrison P.J., Harris L.R., Janke B. (1996). Development of Emission Factors for Polyethylene Processing. J. Air Waste Manag. Assoc..

[168] Molto J., Egea S., Conesa J.A., Font R. (2011). Thermal Decomposition of Electronic Wastes: Mobile Phone Case and Other Parts. Waste Manag..

[169] Ortuno N., Conesa J.A., Molto J., Font R. (2014). Pollutant Emissions during Pyrolysis and Combustion of Waste Printed Circuit Boards, before and after Metal Removal. Sci. Total Environ..

[170] Statheropoulos M., Agapiou A., Pallis G. (2005). A Study of Volatile Organic Compounds Evolved in Urban Waste Disposal Bins. Atmos. Environ..

[171] Christian T.J., Yokelson R.J., Cardenas B., Molina L.T., Engling G., Hsu S.C. (2010). Trace Gas and Particle Emissions from Domestic and Industrial Biofuel Use and Garbage Burning in Central Mexico. Atmos. Chem. Phys..

[172] Woodall B.D., Yamamoto D.P., Gullett B.K., Touati A. (2012). Emissions from Small-Scale Burns of Simulated Deployed U.S.. Mil. Waste Environ. Sci. Technol..

[173] Statheropoulos M., Spiliopouiou C., Agapiou A. (2005). A Study of Volatile Organic Compounds Evolved from the Decaying Human Body. Forensic Sci. Int..

[174] Scheutz C., Bogner J., Chanton J.P., Blake D., Morcet M., Aran C., Kjeldsen P. (2008). Atmospheric Emissions and Attenuation of Non-Methane Organic Compounds in Cover Soils at a French Landfill. Waste Manag..

[175] Tassi F., Capecchiacci F., Cabassi J., Calabrese S., Vaselli O., Rouwet D., Pecoraino G., Chiodini G. (2012). Geogenic and Atmospheric Sources for Volatile Organic Compounds in Fumarolic Emissions from Mt. Etna and Vulcano Island (Sicily, Italy). J. Geophys. Res. Atmos..

[176] Leech J.A., Nelson W.C., Burnett R.T., Aaron S., Raizenne M.E. (2002). It’s About Time: A Comparison of Canadian and American Time-Activity Patterns. J. Expo. Anal. Environ. Epidemiol..

[177] Health Canada (2007). Regina Indoor Air Quality Study (2007): Data Summary for Volatile Organic Compond Sampling.

[178] Lofroth G., Burton R.M., Forehand L., Hammond S.K., Seila R.L., Zweidinger R.B., Lewtas J. (1989). Characterization of Environmental Tobacco Smoke. Environ. Sci. Technol..

[179] Persson K.A., Berg S., Tornqvist M., Scaliatomba G.P., Ehrenberg L. (1988). Note on Ethene and Other Low-Molecular Weight Hydrocarbons in Environmental Tobacco Smoke. Acta Chem. Scand. B..

[180] Shaikh G.N., Pandit G.G., Rao A.M.M., Mishra U.C. (1988). Determination of Ethylene and Other Reactive Hydrocarbons in Tobacco Smoke and Evaluation of Risk from Their Inhalation. Sci. Total Environ..

[181] Filipiak W., Ruzsanyi V., Mochalski P., Filipiak A., Bajtarevic A., Ager C., Denz H., Hilbe W., Jamnig H., Hackl M. (2012). Dependence of exhaled breath composition on exogenous factors, smoking habits and exposure to air pollutants. J. Breath Res..

[182] Mochalski P., King J., Unterkofler K., Hinterhuber H., Amann A. (2014). Emission Rates of Selected Volatile Organic Compounds from Skin of Healthy Volunteers. J. Chromatogr. B.

[183] Mochalski P., King J., Klieber M., Unterkofler K., Hinterhuber H., Baumann M., Amann A. (2013). Blood and Breath Levels of Selected Volatile Organic Compounds in Healthy Volunteers. Analyst.

[184] Bari M.A., Kindzierski W.B., Wheeler A.J., Heroux M.E., Wallace L.A. (2015). Source Apportionment of Indoor and Outdoor Volatile Organic Compounds at Homes in Edmonton, Canada. Build. Environ..

[185] Graham L.A., Noseworthy L., Fugler D., O’Leary K., Karman D., Grande C. (2004). Contribution of Vehicle Emissions from an Attached Garage to Residential Indoor Air Pollution Levels. J. Air Waste Manag. Assoc..

[186] Van Winkle M.R., Scheff P.A. (2001). Volatile Organic Compounds, Polycyclic Aromatic Hydrocarbons and Elements in the Air of Ten Urban Homes. Indoor Air.

[187] Health Canada (2010). Windsor Exposure Assessment Study (2005–2006): Data Summary for Volatile Organic Compond Sampling.

[188] Waring M.S. (2014). Secondary Organic Aerosol in Residences: Predicting Its Fraction of Fine Particle Mass and Determinants of Formation Strength. Indoor Air.

[189] MDEQ (2012). Typical Indoor Air Concentrations of Volatile Organic Compounds in Non-Smoking Montana Residences Not Impacted by Vapor Intrusion.

[190] Wang Y.C., Lin C., Lin Y.K., Wang Y.F., Weng W.H., Kuo Y.M. (2016). Characteristics and Determinants of Ambient Volatile Organic Compounds in Primary Schools. Environ. Sci. Process Impacts.

[191] Mukerjee S., Ellenson W.D., Lewis R.G., Stevens R.K., Somerville M.C., Shadwick D.S., Willis R.D. (1997). An Environmental Scoping Study in the Lower Rio Grande Valley of Texas. 3. Residential Microenvironmental Monitoring for Air, House Dust, and Soil. Environ. Int..

[192] Drakou G., Zerefos C., Ziomas I., Voyatzaki M. (1998). Measurements and Numerical Simulations of Indoor O_3_ and NO_x_ in Two Different Cases. Atmos. Environ..

[193] Health Canada (2009). Halifax Indoor Air Quality Study (2009): Volatile Organic Componds (VOC) Data Summary.

[194] Health Canada (2010). Edmonton Indoor Air Quality Study (2010): Volatile Organic Componds (VOC) Data Summary.

[195] Al-Khulaifi N.M., Al-Mudhaf H.F., Alenezi R., Abu-Shady A.-S.I., Selim M.I. (2014). Seasonal and Tempoal Variations in Volatile Organic Compounds in Indoor and Outdoor Air in Al-Jahra City, Kuwait. J. Environ. Prot..

[196] Duan H., Liu X., Yan M., Wu Y., Liu Z. (2014). Characteristics of Carbonyls and Volatile Organic Compounds (VOCs) in Residences in Beijing, China. Front. Environ. Sci. Eng..

[197] Sarwar G., Corsi R., Kimura Y., Allen D., Weschler C.J. (2002). Hydroxyl Radicals in Indoor Environments. Atmos. Environ..

[198] Huang Y., Ho S.S.H., Ho K.F., Lee S.C., Yu J.Z., Louie P.K.K. (2011). Characteristics and Health Impacts of VOCs and Carbonyls Associated with Residential Cooking Activities in Hong Kong. J. Hazard. Mater..

[199] Lin Y., Shao M., Lu S.H. (2010). The Emission Characteristics of Hydrocarbon from Chinese Cooking under Smoke Control. Int. J. Environ. Anal. Chem..

[200] Mugica V., Vega E., Chow J., Reyes E., Sanchez G., Arriaga J., Egami R., Watson J. (2001). Speciated Non-Methane Organic Compounds Emissions from Food Cooking in Mexico. Atmos. Environ..

[201] Schauer J.J., Kleeman M.J., Cass G.R., Simoneit B.R.T. (1999). Measurement of Emissions from Air Pollution Sources. 1. C_1_ through C_29_ Organic Compounds from Meat Charbroiling. Environ. Sci. Technol..

[202] Cheng S.Y., Wang G., Lang J.L., Wen W., Wang X.Q., Yao S. (2016). Characterization of Volatile Organic Compounds from Different Cooking Emissions. Atmos. Environ..

[203] Olsson M., Petersson G. (2003). Benzene Emitted from Glowing Charcoal. Sci. Total Environ..

[204] Tsai S.M., Zhang J.F., Smith K.R., Ma Y.Q., Rasmussen R.A., Khalil M.A.K. (2003). Characterization of Non-Methane Hydrocarbons Emitted from Various Cookstoves Used in China. Environ. Sci. Technol..

[205] Wang S.X., Wei W., Du L., Li G.H., Hao J.M. (2009). Characteristics of Gaseous Pollutants from Biofuel-Stoves in Rural China. Atmos. Environ..

[206] Olsson M., Kjallstrand J. (2006). Low Emissions from Wood Burning in an Ecolabelled Residential Boiler. Atmos. Environ..

[207] Carteret M., Pauwels J.F., Hanoune B. (2012). Emission Factors of Gaseous Pollutants from Recent Kerosene Space Heaters and Fuels Available in France in 2010. Indoor Air.

[208] Tissari J., Hytonen K., Lyyranen J., Jokiniemi J. (2007). A Novel Field Measurement Method for Determining Fine Particle and Gas Emissions from Residential Wood Combustion. Atmos. Environ..

[209] Pettersson E., Boman C., Westerholm R., Bostrom D., Nordin A. (2011). Stove Performance and Emission Characteristics in Residential Wood Log and Pellet Combustion, Part 2: Wood Stove. Energy Fuels.

[210] Boman C., Pettersson E., Westerholm R., Bostrom D., Nordin A. (2011). Stove Performance and Emission Characteristics in Residential Wood Log and Pellet Combustion, Part 1: Pellet Stoves. Energy Fuels.

[211] Olsson M., Ramnas O., Petersson G. (2004). Specific Volatile Hydrocarbons in Smoke from Oxidative Pyrolysis of Softwood Pellets. J. Anal. Appl. Pyrolysis.

[212] Olsson M. (2006). Wheat Straw and Peat for Fuel Pellets—Organic Compounds from Combustion. Biomass Bioenergy.

[213] Perzon M. (2010). Emissions of Organic Compounds from the Combustion of Oats—A Comparison with Softwood Pellets. Biomass Bioenergy.

[214] Schauer J.J., Kleeman M.J., Cass G.R., Simoneit B.R.T. (2001). Measurement of Emissions from Air Pollution Sources. 3. C_1_–C_29_ Organic Compounds from Fireplace Combustion of Wood. Environ. Sci. Technol..

[215] McDonald J.D., Zielinska B., Fujita E.M., Sagebiel J.C., Chow J.C., Watson J.G. (2000). Fine Particle and Gaseous Emission Rates from Residential Wood Combustion. Environ. Sci. Technol..

[216] Liu C., Zhang C., Mu Y., Liu J., Zhang Y. (2016). Emission of Volatile Organic Compounds from Domestic Coal Stove with the Actual Alternation of Flaming and Smoldering Combustion Processes. Environ. Pollut..

[217] Wang Q., Geng C.M., Lu S.H., Chen W.T., Shao M. (2013). Emission Factors of Gaseous Carbonaceous Species from Residential Combustion of Coal and Crop Residue Briquettes. Front. Environ. Sci. Eng..

[218] Park K.J., Seo T., Jung D. (2007). Performance of Alternative Refrigerants for Residential Air-Conditioning Applications. Appl. Energy.

[219] Yu T.H., Wu C.M., Liou Y.C. (1989). Volatile Compounds from Garlic. J. Agric. Food Chem..

[220] Zeger S.L., Thomas D., Dominici F., Samet J.M., Schwartz J., Dockery D., Cohen A. (2000). Exposure Measurement Error in Time-Series Studies of Air Pollution: Concepts and Consequences. Environ. Health Perspect..

[221] Gulliver J., Briggs D.J. (2005). Time-Space Modeling of Journey-Time Exposure to Traffic-Related Air Pollution Using GIS. Environ. Res..

[222] Zou B., Wilson J.G., Zhan F.B., Zeng Y.N. (2009). Air Pollution Exposure Assessment Methods Utilized in Epidemiological Studies. J. Environ. Monit..

[223] Axelsson G., Barregard L., Holmberg E., Sallsten G. (2010). Cancer Incidence in a Petrochemical Industry Area in Sweden. Sci. Total Environ..

[224] Tsai D.H., Wang J.L., Chuang K.J., Chan C.C. (2010). Traffic-Related Air Pollution and Cardiovascular Mortality in Central Taiwan. Sci. Total Environ..

[225] Lin T.Y., Sree U., Tseng S.H., Chiu K.H., Wu C.H., Lo J.G. (2004). Volatile Organic Compound Concentrations in Ambient Air of Kaohsiung Petroleum Refinery in Taiwan. Atmos. Environ..

[226] Bostrom C.E., Almen J., Steen B., Westerholm R. (1994). Human Exposure to Urban Air Pollution. Environ. Health Perspect..

[227] Weichenthal S., Kulka R., Belisle P., Joseph L., Dubeau A., Martin C., Wang D., Dales R. (2012). Personal Exposure to Specific Volatile Organic Compounds and Acute Changes in Lung Function and Heart Rate Variability Among Urban Cyclists. Environ. Res..

[228] Barrefors G., Petersson G. (1996). Exposure to Volatile Hydrocarbons in Commuter Trains and Diesel Buses. Environ. Technol..

[229] Lin C.S., Liou N.W., Chang P.E., Yang J.C., Sun E. (2007). Fugitive Coke Oven Gas Emission Profile by Continuous Line Averaged Open-Path Fourier Transform Infrared Monitoring. J. Air Waste Manag. Assoc..

[230] Chen C.L., Fang H.Y., Shu C.M. (2006). Mapping and Profile of Emission Sources for Airborne Volatile Organic Compounds from Process Regions at a Petrochemical Plant in Kaohsiung, Taiwan. J. Air Waste Manag. Assoc..

[231] Chen C.L., Fang H.Y., Shu C.M. (2005). Source Location and Characterization of Volatile Organic Compound Emissions at a Petrochemical Plant in Kaohsiung, Taiwan. J. Air Waste Manag. Assoc..

[232] McKenzie L.M., Witter R.Z., Newman L.S., Adgate J.L. (2012). Human Health Risk Assessment of Air Emissions from Development of Unconventional Natural Gas Resources. Sci. Total Environ..

[233] Simpson I.J., Marrero J.E., Batterman S., Meinardi S., Barletta B., Blake D.R. (2013). Air Quality in the Industrial Heartland of Alberta, Canada and Potential Impacts on Human Health. Atmos. Environ..

[234] Ostermark U. (1995). Characterization of Volatile Hydrocarbons Emitted to Air from a Cat-Cracking Refinery. Chemosphere.

[235] Wei W., Cheng S.Y., Li G.H., Wang G., Wang H.Y. (2014). Characteristics of Volatile Organic Compounds (VOCs) Emitted from a Petroleum Refinery in Beijing, China. Atmos. Environ..

[236] Su Y.C., Chen S.P., Tong Y.H., Fan C.L., Chen W.H., Wang J.L., Chang J.S. (2016). Assessment of Regional Influence from a Petrochemical Complex by Modeling and Fingerprint Analysis of Volatile Organic Compounds (VOCs). Atmos. Environ..

[237] Schade G.W., Roest G. (2016). Analysis of Non-Methane Hydrocarbon Data from a Monitoring Station Affected by Oil and Gas Development in the Eagle Ford Shale, Texas. Elementa.

[238] Jankovic J., Jones W., Burkhart J., Noonan G. (1991). Environmental Study of Firefighters. Ann. Occup. Hyg..

[239] Austin C.C., Wang D., Ecobichon D.J., Dussault G. (2001). Characterization of Volatile Organic Compounds in Smoke at Municipal Structural Fires. J. Toxicol. Environ. Health A.

[240] Fent K.W., Evans D.E. (2011). Assessing the Risk to Firefighters from Chemical Vapors and Gases during Vehicle Fire Suppression. J. Environ. Monit..

[241] Fent K.W., Evans D.E., Booher D., Pleil J.D., Stiegel M.A., Horn G.P., Dalton J. (2015). Volatile Organic Compounds Off-Gassing from Firefighters’ Personal Protective Equipment Ensembles after Use. J. Occup. Environ. Hyg..

[242] Purohit V., Orzel R.A. (1988). Polypropylene—A Literature Review of the Thermal Decomposition Products and Toxicity. J. Am. Coll. Toxicol..

[243] Blais M., Carpenter K. (2015). Flexible Polyurethane Foams: A Comparative Measurement of Toxic Vapors and Other Toxic Emissions in Controlled Combustion Environments of Foams with and without Fire Retardants. Fire Technol..

[244] Austin C.C., Wang D., Ecobichon D.J., Dussault G. (2001). Characterization of Volatile Organic Compounds in Smoke at Experimental Fires. J. Toxicol. Environ. Health A.

[245] Bogner J.E., Chanton J.P., Blake D., Abichou T., Powelson D. (2010). Effectiveness of a Florida Landfill Biocover for Reduction of CH_4_ and NMHC Emissions. Environ. Sci. Technol..

[246] Ramadan A. (2017). Assessment of Spatial Variation of Ambient VOCs Levels at a Power Station in Kuwait. J. Air Waste Manag. Assoc..

[247] Huang C.H., Chen K.S., Wang H.K. (2012). Measurements and PCA/APCS Analyses of Volatile Organic Compounds in Kaohsiung Municipal Sewer Systems, Southern Taiwan. Aerosol Air Qual. Res..

[248] Singh A., Spak S.N., Stone E.A., Downard J., Bullard R.L., Pooley M., Kostle P.A., Mainprize M.W., Wichman M.D., Peters T.M. (2015). Uncontrolled Combustion of Shredded Tires in a Landfill—Part 2: Population Exposure, Public Health Response, and an Air Quality Index for Urban Fires. Atmos. Environ..

[249] Trabue S., Scoggin K., Li H., Burns R., Xin H.W., Hatfield J. (2010). Speciation of Volatile Organic Compounds from Poultry Production. Atmos. Environ..

