# Supplementary material for: The Human Exposure Potential from Propylene Releases to the Environment
Source: Int J Environ Res Public Health. 2018 Jan 4;15(1):66. doi: 10.3390/ijerph15010066 (PMC5800165; doi:10.3390/ijerph15010066)
Supplement: Supplementary file 1 [file ijerph-15-00066-s001.pdf]

Supplemental Information

The following tables (S1-S6) summarize the results from ambient air monitoring campaigns conducted throughout the world. The measurements are categorized by continent and listed in chronological order. Values are also provided for marine environments and other locations that were difficult to classify. Ground level and airborne values have been included to provide some sense of the vertical distribution of propylene in the troposphere. Likewise, urban, rural, and remote measurements offer some indication of the spatial differences that exist. The review is not comprehensive and does not capture the levels that were found earlier than about 40 years ago.

Table S1: Ambient air concentrations of propylene in Asia

Location	Sampling Date(s)	Monitoring station	Avg. Conc. (ppbv)	Variability[#] (ppbv)	Sampling Details	Reference
Japan	1980-1981	airborne 1<br>airborne 2	0.7<br>0.3	0.9<br>0.7	day and night aircraft flights over Tokyo for the years 1980 (#1) and 1981 (#2) at altitudes of 350-600m	[250]
China	1985-1987	rural	0.24	0.0002-0.59†	samples collected around an agricultural area with rice fields and biogas generators nearby	[251]
Japan	1987-1990	urban 1<br>urban 2<br>urban 3	0.49<br>0.50<br>0.80	NR<br>NR<br>NR	summertime samples taken in the city of Osaka at three urban locations; Konohana (#1), Yodogawa (#2), and Setsuyo (#3)	[252]

Japan	1987-1990	roadside 1<br>roadside 2	4.70<br>2.40	2.70-8.64†<br>0.77-4.97†	summer and winter averages for samples collected in Osaka near the busy highways of Umedashinmichi (#1) and Dekijima (#2)	[253]
Japan	1992-1993	urban	0.6	3.6	urban industrial location with nearby highways on a rooftop in central Osaka	[254]
India	1993-1994	urban	0.2+	NR	average for samples collected at 5 industrial location in Bombay at sites situated away from major traffic arteries	[255]
Korea	1996-1997	urban 1<br>urban 2	2.1<br>11.7	1.1<br>9.5	samples collected downtown in the city Ulsan near a busy roadway (#1) or near a petrochemical complex (#2)	[256]
Taiwan	1997	urban	4.6	0.5-14.4†	samples taken at 50 background locations away from traffic throughout the Taipei metropolitan area	[257]
Japan	1998	remote	0.106	0.027-0.258†	sampling on an island shoreline with no nearby population centers	[258]

Korea	1998-1999	urban	1.8	0.6	monitoring atop a 4 story building in a residential and commercial area of Seoul	[259]
Pakistan	1998-1999	urban	5.5	0.06-19.9†	various roadside, industrial, commercial, and residential sampling sites in the city of Karachi	[260]
Taiwan	1998-1999	rural 1<br>rural 2	9.6<br>2.1	NR<br>NR	average concentration from a windward (#1) and leeward (#2) site for an air mass traveling northeast during the monsoon season	[261]
Japan	1998-1999	remote	0.101	0.078	summer measurements at an altitude of 1840 m on a mountain with no nearby emission sources	[262]
Nepal	1998	urban	12.75	0.63-34.48	sampling at a busy roadside in Katmandu	[263]
China	1999-2001	rural	0.34	0.30	sampling site in a rural area surrounded by small villages, agricultural fields and forests	[264]
China	2000-2001	urban	2.80	NR	monitoring near a roadside in Hong Kong with mixed commercial and residential areas	[265]

China	2000	urban 1<br>urban 2	3.9<br>0.3	NR<br>NR	average from two samples collected inside (#1) and outside (#2) a car park in Hong Kong	[266]
China	2000	urban 1<br>urban 2<br>suburban	1.1<br>0.6<br>0.4	0.8<br>0.3<br>0.2	sampling from 78 sites located in five cities where the monitor was located in an area with industrial sources (#1), mixed urban and industrial sources (#2), or a suburban location with mixed industrial and local sources	[267]
Japan	2000	remote 1<br>remote 2<br>remote 3	0.056<br>0.033<br>0.035	0.078<br>0.017<br>0.034	single sampling site with values sorted according to the air mass source; transport from China (#1), Korea & Shandong (#2), the north or east (#3)	[268]
Taiwan	2000	urban 1<br>urban 2	5.4<br>6.9	NR<br>NR	winter (January) averages for sampling sites located in non-industrial (#1) areas and industrial (#2) areas in the city of Kaohsiung	[269]
China	2001-2002	rural	0.223	0.011-2.178†	monitoring at a coastal site receiving air from major anthropogenic emission sources	[270]

China	2001-2002	rural 1<br>rural 2	0.056<br>0.235	0.036<br>0.301	monitoring at a coastal area that is heavily forested, rural 1 include days with clean maritime air mass inflow and rural 2 include measurements with a continental air mass outflow	[271]
China	2001	urban	---	0.2-8.2	measurements from 43 Chinese cities	[272]
China	2001	rural	0.061	0.018-0.214†	rural site located 10 km upwind of Hong Kong at southeastern tip of the island	[273]
China	2002-2003	urban<br>suburban 1<br>suburban 2<br>rural	0.315<br>0.246<br>0.606<br>0.141	0.036≈<br>0.041≈<br>0.075≈<br>0.021≈	sampling in and around Hong Kong including an urban residential area; suburban sites near the airport (#1) and an industrial area (#2); and a rural location outside the city	[274]
India	2002	urban	3.73	3.70	levels taken on the outskirts of the large city Ahmedabad	[275]
India	2002	remote	0.16	0.06	sampling on a mountain peak at 1680 m with no emission sources nearby	[276]

Taiwan	2002	urban<br>suburban<br>rural	2.2<br>1.6<br>0.7	1.8<br>1.0<br>0.7	average values from metropolitan and downwind suburban sites in an around Kaohsiung compared with a rural site outside the city	[277]
Taiwan	2003-2006	urban	1.7	0.29-6.71†	background sampling from university building rooftop (15 m) in downtown Taichung	[278]
Japan	2003-2004	urban	0.705	0.717	monitoring at a campus building located in the city of Nagoya	[279]
Taiwan	2003	urban 1<br>urban 2	2.60<br>3.04	2.37<br>2.86	samples taken in northern (#1) or southern (#2) sites within the heavily industrialized city of Kaohsiung	[280]
Korea	2004-2008	urban	0.43	0.33	measurements recorded on a rooftop building in Seoul with no strong emission sources nearby	[281]
China	2004	remote	0.16	0.46	autumn averages from a shoreline mountain situated in a rain forest on a island in the south China sea	[282]

China	2004	urban<br>rural<br>remote 1<br>remote 2	0.86<br>0.33<br>0.17<br>0.11	0.22-1.89†<br>0.09-2.11†<br>0.09-0.50†<br>0.05-0.77†	urban samples collected in the city of Tengyue; rural samples from a rooftop with nearby villages; the remote samples came from atop a mountain on the mainland (#1) or on an island with a surrounding rain forest (#2)	[283]
India	2004	rural	0.206	NR	average value from eight rural sites located away from urban centers, industries, and major roadways	[284]
India	2004	urban 1<br>urban 2	2.34<br>2.20	1.94<br>1.47	sampling at a university campus near the city of Hisar with no nearby heavy industry or traffic (#1) and a campus in the city Kanpur (#2) with heavy industry nearby	[285]
Japan	2004	urban 1<br>suburban 2	1.16‡<br>0.32‡	0.29-23.4†<br>0.04-3.36†	summer daytime averages for a university site in the central core of Tokyo (#1) and a suburban campus in the city of Kisai (#2)	[286]

Korea	2004	urban	2.12	0.01-18.7†	urban sampling atop a building near the center of Seoul with nearby roadways and small manufacturing facilities	[287]
Taiwan	2004	urban 1<br>urban 2	8.0<br>15.8	0.5<br>6.2	average from four samples collected in the industrial sectors of Tzouying (#1) and Daliao (#2) in Kaohsiung City	[288]
China	2005	urban 1<br>urban 2	0.81<br>0.53	0.12-2.21†<br>0.11-1.31†	multiple measurements from the urban core 2 cites	[289]
China	2005	urban	2.26	2.10	sampling on the roof of a university building in an area surrounded by petrochemical plants and expressways	[290]
China	2005	urban 1<br>suburban 2<br>commercial 3<br>rural 4<br>remote 5<br>roadside 6<br>roadside 7	2.36<br>1.10<br>8.95<br>0.43<br>0.13<br>2.91<br>21.9	1.34<br>0.76<br>11.80<br>0.25<br>0.07<br>1.28<br>0.41	sampling at various locations including an urban core (#1), a suburban neighborhood (#2), a printing factory rooftop (#3), a tropical forest (#4), a rainforest (#5), and two busy roadways (#6 and #7)	[291]

China	2005	urban	1.16	0.73	sampling on a university building rooftop in an area of Beijing surrounded by busy roadways and industries	[292]
China	2005	urban	2.26	NR	sampling from the roof (12 m) of university building located in the city of Guangzhou with nearby commercial, residential, industrial, and traffic-related emission sources	[290]
China	2006-2007	urban 1<br>urban 2<br>urban 3<br>urban 4<br>rural	1.7<br>0.9<br>1.5<br>0.8<br>1.0	0.5-5.1†<br>0.6-1.3†<br>0.4-3.9†<br>0.5-1.7†<br>0.8-1.1†	sampling in the industrialized urban core of Shanghai (#1), the residential area of Taizhou (#2), the residential area of Liyang,, and the remote island of Chongming with farmland and lush vegetation	[293]
China	2006-2007	urban	1.70	NR	sampling from a rooftop (20 m) near a major roadway in the city of Shanghai	[294]
India	2006-2007	urban	3.4	1.3	average from three locations in the city of Raipur with local heavy traffic and various industries	[295]

China	2006-2007	urban 1	0.93	1.07	daily average for a high traffic sampling site in central Shanghai	[296]
China	2006-2008	urban 1<br>urban 2	1.51<br>1.60	0.38-7.43<br>0.23-8.31	samples collected in the summer (#1) and winter (#2) in the center of Shanghai with nearby residential and commercial buildings and roadway traffic	[297]
China	2006	urban	2.96	NR	sampling at a Beijing campus location with no nearby emission sources or heavy road traffic	[298]
China	2006	urban<br>rural	0.40<br>0.90	0.23<br>0.45	samples collected in an around Beijing in the urban core or a rural site outside the city with no strong emission sources	[299]
China	2006	remote	0.110	0.031-0.201†	sampling atop a mountain (1534 m) where the impact of local anthropogenic emissions are minimal	[300]
China	2006	urban	3.32	0.98	sampling atop a building (50 m) on a university campus in Beijing with no major emission sources nearby	[301]

Japan	2007-2008	industrial 1<br>industrial 2<br>industrial 3<br>suburban 4<br>suburban 5<br>suburban 6	0.8<br>2.2<br>2.0<br>2.4<br>1.0<br>1.2	0.6<br>3.1<br>0.7<br>5.1<br>1.0<br>1.4	samples collected in Yokohama city near the industrial locations of Negishi (#1), Honmoku (#2), and Shiohama (#3) or at the suburban residential sites of Minezawa (#4), Sakuragicho (#5), and Tsurumi (#6) where traffic and industrial influences existed	[302]
Taiwan	2007-2011	urban 1<br>urban 2<br>industrial 1<br>industrial 2	1.10<br>1.10<br>1.28<br>2.51	0.13<br>0.09<br>0.38<br>0.15	urban sites 1 and 2 are located in an urban core; industrial site 1 is in a rural location and industrial site 2 has an urban location	[303]
China	2007-2010	urban	0.84	0.06-3.14†	sampling in the urban core of Shanghai with no nearby industries	[304]
China	2007	rural	0.162	0.037-0.461†	samples collected at an observatory in a national park located 40 km downwind from Beijing	[305]
China	2007	rural	0.37	0.15-1.98†	rural site surrounded by farmland with little traffic, industry, or population	[306]

					density	
China	2007	suburban	1.051	0.469-1.506*	rooftop sampling at a university site in a residential area in the city of Changsha	[307]
China	2007	surface<br>airborne 1<br>airborne 2	1.125<br>0.248<br>0.141	1.585<br>0.269<br>0.112	surface samples were collected at ground level at various urban and rural locations in the province of Jilin; airborne samples were collected aboard an aircraft traveling below 2 km in the planetary boundary layer (#1) or in the free troposphere (#2)	[308]
China	2008	urban 1<br>urban 2	5.03<br>9.92	3.54-6.97†<br>2.26-20.20†	samples collected on the roof of a building in the center Foshan during with normal (#1) and high PM_{10} (#2) levels	[309]
China	2008	urban	6.84	4.07	sampling site locate in the center of the Foshan atop a building with nearby traffic	[310]
China	2008	urban<br>rural	7.51<br>1.55	NR<br>NR	sampling sites located in the city of Beijing or in a rural agricultural area with small villages nearby	[311]

China	2008	urban 1<br>urban 2	0.88<br>0.55	0.31<br>0.72	average levels in June before the Olympic games (#1) and August during the games (#2) atop a university building 5 km from the Olympic Park	[312]
China	2008	urban 1<br>urban 2	1.6<br>1.2	0.8<br>0.6	morning (#1) and afternoon (#2) sampling on a roof near a residential area of Beijing with no industrial sources nearby	[313]
Taiwan	2008	urban 1<br>urban 2<br>urban 3<br>urban 4<br>urban 5	5.31<br>4.37<br>6.12<br>7.36<br>6.30	NR<br>NR<br>NR<br>NR<br>NR	early morning (7 to 9 AM) sampling near busy intersection in Kaohsiung at elevations of 2m (#1), 13 m (#2), 32 m (#3), 58 m (#4), and 111 m (#5)	[314]
Thailand	2008	urban<br>suburban	3.458<br>0.500	NR<br>NR	urban site located in a residential area with nearby roadways inside the city of Bangkok; suburban site located outside the city on a university campus with nearby residential areas	[315]
India	2009-2011	remote	0.6	0.7	high altitude (1958 m) sampling on a peak in the Himalayas	[316]

China	2010-2012	urban	1.88	2.46	sampling atop a building (15 m) in a commercial and residential zone of Jinan with high traffic in the area	[317]
Taiwan	2011-2012	urban 1<br>urban 2	0.56<br>0.53	NR<br>NR	daytime sampling from a Taipei roof top in summer (#1) and autumn (#2) with busy streets, shops, and residential apartments nearby	[318]
China	2011-2012	urban	1.569	0.883-2.121†	site located in a residential and commercial area with heavy traffic nearby	[319]
Bangladesh	2011	suburban	0.63	0.32	samples collected on a university campus in a residential area near the city of Dhaka	[320]
China	2011	rural	0.30	0.28	sampling on a 30 m high hill located on an island off the mainland	[321]
Nepal	2012-2013	suburban	3.98	1.21	sampling from a rooftop (20 m) in mountain valley prone to pollution from nearby cities	[322]
Saudi Arabia	2012-2013	urban 1<br>urban 2<br>urban 3	4.0<br>3.9<br>3.3	0.14-9.3†<br>0.16-14.6†<br>0.13-14.0†	sampling in residential/commercial areas of Jeddah (#1), Mecca (#2), and Madina (#3)	[323]

China	2012-2013	suburban	1.7	1.4	measurements at a suburban location with little traffic or industry and small burning events	[324]
India	2012-2013	urban	1.8	1.7	background sampling on a university campus in the middle of Kolkata	[325]
Pakistan	2012	urban	18.3	1.5-79.5†	monitoring in the city of Lahore at various residential, commercial, and tourist sites	[323]
Singapore	2012	urban	0.78	0.05-11.3†	sampling site locate atop a university building (65 m) located in the city	[323]
Taiwan	2012	urban	0.82	NR	sampling in an urban area of Taipei affected by both industrial and traffic-related emission sources	[326]
China	2013	rural 1<br>rural 2	0.666<br>0.214	0.525<br>0.132	averages from a rural agricultural region during periods of biomass burning (#1) and non-burning (#2)	[327]
China	2013	urban 1<br>urban 2	1.80<br>0.23	0.42<br>0.07	sampling near roadways (#1) or in general background (#2) locations in Hong Kong	[328]

China	2013	suburban	1.70	0.98	sampling near a busy industrial park with roads and forests nearby	[329]
China	2013	urban	2.43	NR	samples collected downtown in the city of Lanzhou with many petrochemical industries nearby	[330]
China	2013	rural	0.84	1.66	sampling at the Gucheng agricultural station where urban air masses may influence measurements	[331]
India	2013	urban	2.02	NR	urban sampling in the city of Hyderabad with many industries and traffic-related emission sources	[332]
China	2014	suburban	1.3	1.2	samples collected atop an office building (20 m) on a university campus outside Beijing with moderate traffic nearby	[333]

[a] values reported as ppbC or μg/m^3 were converted to ppbv
unless otherwise stated variability is expressed in term of the standard deviation
^ 5th-95th percentile range for the distribution of values
* 25th-75th percentile range for the distribution of values
∞ 90% confidence interval
≈ 95% confidence interval
† minimum and maximum
Δ maximum value
+ geometric mean
‡ median value
NR not reported

Table S2: Ambient air concentrations of propylene in North America

Location	Sampling Date(s)	Monitoring station	Avg. Conc. (ppbv)	Variability[#] (ppbv)	Sampling Details	Reference
USA	1975-1981	urban<br>rural	2.81<br>1.61	1.48<br>2.08	urban average for 7 metropolitan centers; rural average for 3 locations across the USA	[334]
USA	1978	remote 1<br>remote 2	1.9<br>1.1	NR<br>NR	mid (10:00-10:20 AM) morning (#1) and mid (2:15-2:45) afternoon (#2) samples collected at a national park in Tennessee with mixed coniferous and deciduous trees	[335]
USA	1981	urban	0.50	NR	sampling at a university campus located in mid-town Atlanta	[336]
USA	1984-1986	urban	2.6‡	0.1-151.7†	average level for samples collected near the urban core of 39 cities	[337]
USA	1986-1987	urban<br>suburban<br>industrial	1.35<br>1.13<br>1.24	0.32-1.73†<br>0.38-2.00†<br>0.49-1.73†	urban site near the center of Chicago; suburban site 80 km outside the city; industrial site just outside the city	[338]
USA	1989	remote	0.231	0.064	springtime averages at an observatory in Barrow Alaska	[339]

USA	1989	urban	0.58	0.49	samples collected at six downtown locations in the city of Columbus	[340]
USA	1991-1992	remote 1<br>remote 2	0.002‡<br>0.004‡	0.001-0.002*<br>0.002-0.006*	winter averages from an observatory at an elevation of 3.4 km in Hawaii; remote 1 samples when the site sees free tropospheric air; remote 2 samples from air movement up the slope	[341]
Canada	1991-1993	urban	1.44	NR	downtown urban core in the city of Alberta	[342]
Canada	1991	rural 1<br>rural 2<br>rural 3<br>rural 4	0.07<br>0.06<br>0.09<br>0.11	0.03<br>0.02<br>0.04<br>0.09	measurements at four forested sites located in Nova Scotia (#1), Quebec (#2), Ontario (#3), and British Columbia (#4)	[343]
USA	1992-1993	rural 1<br>rural 2<br>rural 3<br>rural 4	0.24<br>0.20<br>0.54<br>0.58	0.26<br>0.10<br>0.70<br>0.49	summertime averages at 4 rural locations in Alabama (#1), Mississippi (#2), Georgia (#3), and North Carolina (#4)	[344]
USA	1993	urban 1<br>urban 2	0.52<br>0.41	0.12-1.51†<br>0.29-0.64†	sampling a single urban site in Brownsville, Texas for the spring (#1) and summer (#2) seasons	[345]

USA	1994-1995	remote 1<br>remote 2	NR<br>NR	0.03-0.08†<br>0.02-0.06†	summertime averages from a lodge at 600 m (#1) and on the summit at 1400 m (#2) of forested Whiteface mountain in New York state	[346]
USA	1994	remote 1<br>remote 2	0.18<br>0.17	NR<br>NR	daytime (#1) and nighttime (#2) sampling on a 1.5 km mountain summit in New York State with nearby mixed coniferous and deciduous trees	[347]
USA	1995-1997	remote 1<br>remote 2<br>remote 3	1.56<br>1.81<br>1.09	NR<br>NR<br>NR	remote monitoring three National parks in Kentucky (#1), Tennessee (#2), and Virginia (#3)	[348]
USA	1995	rural 1<br>rural 2	1.11<br>0.35	NR<br>NR	samples collected 10 m above agricultural fields in North Carolina growing maize (#1) or soybeans (#2)	[349]
USA	1996-2004	urban	0.61	0.00-1.76^	urban background location in Dallas with no adjacent commercial, residential, or traffic emission sources	[350]

USA	1996	varied	NR	0.23-3.02	13 monitoring sites throughout the USA including a mix of urban, suburban, rural, and industrial locations.	[351]
USA	1997	remote	0.080	0.053	sampling site on ocean shore Olympic Peninsula in Washington state	[352]
USA	1999-2005	urban	0.190	0.128	urban background average for 28 cities in the United States at sites with no nearby roadways	[353]
USA	1999-2009	urban 1<br>urban 2	1.0‡<br>0.4‡	0.0-9.9†<br>0.0-7.6†	average values for 1999 (#1) and 2009 (#2) from 12 monitoring stations located throughout the California South Coast air basin	[354]
Canada	2000-2009	urban 1<br>urban 2	0.68<br>0.98	NR<br>NR	monitoring sites located in Montreal near an industrial park (#1) or downtown area (#3)	[355]
Canada	2000	remote	0.029	0.010	sampling at an ice camp above the arctic circle in an area near Nunavut	[356]

USA	2000	suburban	0.45‡	111Δ	samples collected near an airport in the city of La Porte Texas at a site that was not heavily influenced by local traffic	[357]
USA	2001-2002	urban 1<br>urban 2<br>urban 3<br>urban 4	4.1<br>2.2<br>3.0<br>1.6	0.4-21.1†<br>0.8-5.1†<br>0.2-13.3†<br>0.1-6.0†	samples collected in close proximity to the ruins of the World Trade Center on the north (#1), east (#2), and west (#3) sides of the collapse, and at a more distant elevated (50 m) site (#4)	[358]
USA	2002-2012	urban 1<br>urban 2<br>urban 3<br>urban 4<br>urban 5	7.90<br>5.78<br>3.33<br>3.17<br>3.01	NR<br>7.17<br>NR<br>6.53<br>3.04	monitoring sites located near refineries in and around Houston at HRM-7 (#1), Baytown (#2), HRM-8 (#3), HRM-3 (#4) and Groves (#5) monitoring stations	[359]
USA	2002	remote	0.022	0.013-0.043*	coastal site in California unaffected by local emission sources	[360]
USA	2002	urban	0.214‡	0.147-0.306*	wintertime average for samples taken in a park outside the city of Pittsburgh	[361]

USA	2003	urban 1<br>urban 2<br>urban 3	4.7<br>3.0<br>9.6	12.7<br>9.0<br>26.1	samples were collected in Houston at the Wallisville (#1), HRM-3 (#2), and Lynchburg monitoring stations located near petrochemical facilities	[362]
USA	2004-2008	remote	0.059	0.009-0.427†	monthly sampling atop a 15 m tower at a New Hampshire observatory surrounded by forest and agricultural fields	[363]
Canada	2004-2006	urban<br>industrial	0.34<br>0.23	0.43<br>0.23	sampling in the town of Fort Saskatchewan and downwind of an oil sands mining site	[364]
Canada	2004	rural	0.023	0.009	average for all measurements from a pasture near a coastline in Nova Scotia	[365]
Canada	2005	urban 1<br>urban 2<br>urban 3<br>rural	0.69<br>0.54<br>0.48<br>0.04	NR<br>NR<br>NR<br>NR	urban air samples collected in Vancouver (#1), Edmonton (#2), and Toronto (#3); the rural sample was taken in Egbert	[366]

USA	2006	urban	1.11	0.03-28.43†	sampling 60 m atop a university building located 5 km from downtown Houston in an area affected by air masses affected by various emission sources	[367]
USA	2007-2008	urban<br>industrial<br>park 1<br>park2	2.44<br>1.92<br>0.63<br>0.47	1.33<br>1.27<br>0.58<br>0.14	average over 8 days; urban site was within downtown Angeles; industrial site was located with an urban area in or around LA; park1 site was in open area; park 2 site included 8 city parks	[368]
USA	2007-2008	semi-remote 1<br>semi-remote 2	0.156<br>0.006	0.050-0.282†<br>0.003-0.014†	hourly samples collected at an observatory outside Los Angeles during the daytime (#1) and nighttime (#2) hours of February	[369]
Canada	2008	airborne	0.028	0.007-0.128†	flights over the oil sands mining and processing site in Alberta	[370]

USA	2008	urban	0.98	NR	air monitoring station located in the city of Burbank, California in an area impacted by emissions from Los Angeles	[371]
USA	2011	rural	0.16	0.012-1.5†	samples taken at an observatory in Colorado with 22 natural gas wells located nearby	[372]
USA	2011	remote	0.104	0.005-1.680†	sampling atop a 300 m tower located at an observatory Colorado with nearby agricultural region	[373]
USA	2013	rural	0.32	0.04-0.73†	measurements taken in a rural town Colorado with numerous gas wells nearby	[374]

[a] values reported as ppbC or μg/m^3 were converted to ppbv

[#] unless otherwise stated variability is expressed in term of the standard deviation

^ 5th-95th percentile range for the distribution of values

* 25th-75th percentile range for the distribution of values

∞ 90% confidence interval

≈ 95% confidence interval

† minimum and maximum

Δ maximum value

+ geometric mean

‡ median value

NR not reported

Table S3: Ambient air concentrations of propylene in South America

Location	Sampling Date(s)	Monitoring station	Avg. Conc. (ppbv)	Variability# (ppbv)	Sampling Details	Reference
Venezuela	1993-1994	remote	0.38	0.16	sampling at the remote mountaintop (2100 m) of Auyantepuy located far from pollution sources but with lush vegetation nearby	[375]
Venezuela	1993-1994	remote	0.41	0.15	remote mountaintop (2100 m) measurements on Auyantepuy located far from pollution sources but with lush vegetation nearby	[376]
Mexico	1993	urban 1<br>urban 2<br>urban 3<br>rural	5.56<br>4.44<br>39.20<br>0.06	NR<br>NR<br>NR<br>NR	samples collected in the morning (#1) or at noon (#2) from in the urban core of Mexico City, near a busy highway (#3) or at a rural site outside the city	[377]
Columbia	2008-2009	urban 1<br>urban 2<br>urban 3	0.07<br>0.83<br>0.50	0.27<br>1.27<br>0.83	urban air samples from background (#1), industrial (#2), and commercial (#3) locations in Bogotá	[378]

Brazil	1996	urban	16.4	NR	site located in the center of Porto Alegre near a busy highway and bus terminal	[379]
Mexico	2002-2003	urban<br>rural<br>industrial	5.93<br>1.33<br>10.96	3.17<br>1.46<br>5.36	average for 4 urban locations with varied background sources; 3 rural locations and a single industrial site	[380]
Brazil	2004	urban 1<br>urban 2	2.2<br>2.9	NR<br>NR	early (#1) and late (#2) morning samples collected in a commercial area of Rio de Janeiro with a high traffic density	[381]
Brazil	2006-2008	urban 1<br>urban 2	2.00<br>2.77	0.01<br>0.02	samples collected in a high traffic area of Sao Paulo for the year 2006 (#1) and 2008 (#2)	[382]
Mexico	2006	suburban 1<br>suburban 2	4.005<br>1.092	3.580<br>1.152	daily averages from sampling sites located downwind of Mexico City 11 km and (#1) and 32 km (#2) northeast of the city	[383]
Mexico	2010	suburban	2.79	2.52	sampling sites located in a park near Tijuana in the vicinity (8 km) of major roadways and industrial sources	[384]

Mexico	2011-2012	urban 1<br>urban 2	20.2<br>14.6	6.3∞<br>6.8∞	winter (Nov-Dec) sampling in downtown Mexico City on a roof located in Pedregal (#1) and Merced (#2)	[385]
Mexico	2011-2012	urban 1<br>urban 2<br>urban 3	0.7<br>0.8<br>2.3	0.3<br>0.6<br>1.4	sampling in downtown Monterrey during the spring of 2011 (#1), spring of 2012 (#2), and fall of 2012 (#3)	[386]
Mexico	2012	urban<br>suburban	11.96<br>6.47	3.38<br>1.95	centrally located urban site (La Merced) in Mexico City that has many emission sources including light industry and heavy traffic; suburban site (Pedregal) has light traffic and no major sources	[387]
Brazil	2013	urban	1.26	0.09-11.77	background sampling at a university campus outside Sao Paulo in an area with heavy traffic	[388]
Chili	2005	urban	3.80	38.8$^{\Delta}$	samples taken on a university campus in the center of Santiago near a busy roadway	[389]

^{a} values reported as ppbC or μg/m^3 were converted to ppbv
unless otherwise stated variability is expressed in term of the standard deviation
^ 5th-95th percentile range for the distribution of values
* 25th-75th percentile range for the distribution of values

∞ 90% confidence interval
≈ 95% confidence interval
† minimum and maximum
Δ maximum value
+ geometric mean
‡ median value
NR not reported

Table S4: Ambient air concentrations of propylene in Europe

Location	Sampling Date(s)	Monitoring station	Avg. Conc. (ppbv)	Variability[#] (ppbv)	Sampling Details	Reference
Portugal	NR	rural 1<br>rural 2	0.458<br>1.340	NR<br>NR	winter day (#1) and night (#2) averages for a rural location in large agricultural area with a small village nearby	[390]
Ireland	NR	urban 1<br>urban 2<br>urban 3	0.74<br>0.26<br>0.32	0.45<br>0.01<br>0.06	monitoring took place near a road (#1), in a park (#2), and on the roof (#3) of a university campus in Dublin	[391]
The Netherlands	1981-1991	urban 1<br>urban 2	0.40<br>0.80	0.03<br>0.10	winter levels in an industrial section of Moerdijk; levels categorized by whether the wind direction and air mass was marine (#1) or continental (#2) in origin	[392]
United Kingdom	1982-1986	airborne	0.046	0.025	background samples collected via aircraft over the ocean water surrounding the UK at altitudes of 1500 to 3000 m	[393]

United Kingdom	1983	urban<br>rural 1<br>rural 2	3.30<br>1.37<br>1.40	2.37<br>1.03<br>1.23	urban samples collected in the city of Lancaster; rural 1 samples taken on days where ozone formation unfavorable; rural 2 samples taken at same site but ozone formation was favorable	[394]
Austria	1986-1987	rural 1<br>rural 2<br>rural 3	0.6<br>0.2<br>0.3	0.4<br>0.1<br>0.1	samples taken atop a tower (80 m) in a beach forest area (#1); from a rooftop (60 m) in an agricultural region (#2), or from a spruce forest (#3)	[395]
Germany	1986-1987	urban<br>suburban<br>industrial	1.9<br>1.9<br>3.4	NR<br>NR<br>NR	urban location in Hamburg with dense traffic and nearby harbor; suburban location outside city center; industrial location with nearby refineries	[396]
France	1986	ground<br>airborne 1<br>airborne 2<br>airborne 3<br>airborne 4<br>airborne 5	0.535<br>0.299<br>0.210<br>0.259<br>0.281<br>0.242	NR<br>NR<br>NR<br>NR<br>NR<br>NR	ground level and hot air balloon measurements over a rural agricultural region at altitudes of 270 m (#1), 460 m (#2), 750 m (#3), 990 m (#4), and 1300 m (#5)	[397]

Hungary	1987-1989	urban 1<br>urban 2	3.7<br>2.8	4.5<br>2.8	sampling on roof tops (20 m) in the urban core (#1) and downtown perimeter (#2) of Budapest	[398]
Norway	1988-1994	remote<br>rural	0.073<br>0.138	0.025<br>0.035	remote sampling on an island observatory above the aortic circle (Zeppelin) and the small rural village of Birkenes with nearby forests	[399]
Germany	1989-1994	forest	0.09	0.04-0.10*	station located on a ridge in forest 10 km from an urban center	[400]
Sweden	1989-1990	rural	0.095	0.095	sampling site in the village of Rorvik on the west coast	[401]
Sweden	1989	urban	15.1	NR	busy intersection in the city of Goteborg	[402]
Latvia	1992-1994	rural	0.191	0.117	sampling at a sparsely populated site in Rucava with nearby forests and agricultural lands	[399]
France	1992-1993	remote	0.21	0.12	monitoring station located at the Atlantic shoreline	[403]
United Kingdom	1991	urban	76.5	13.4-137.5†	sampling close to roadway in central London during a very high pollution period	[404]

Germany	1992-1995	rural	0.66	0.47	monthly averages from a sampling site located in a meadow with surrounding towns	[405]
Switzerland	1992-1994	rural	0.376	0.214	sampling at Tanikan on the Swiss plateau with some local traffic and villages nearby	[399]
Germany	1992-1994	rural	0.250	0.157	sampling at rural forested site in Waldhof impacted by local emissions alone	[399]
Czech Republic	1992-1994	rural	0.235	0.109	sampling a rural location in Kosetice with little traffic and open terrain	[399]
Switzerland	1993-1994<br>2005-2006	urban 1<br>urban 2	0.42<br>0.90	0.32<br>0.76	sampling for two time periods at a background site in the center of Zurich	[406]
Finland	1993-1994	remote 1<br>remote 2	0.050<br>0.021	0.022<br>0.009	monitoring stations located above the Arctic Circle (#1) or on a small island in the Baltic Sea	[407]
Italy	1993	urban	9.93	NR	station located in the center of Rome with high traffic density	[408]

Denmark	1994-1995	urban 1<br>urban 2	2.6<br>0.8	NR<br>NR	street level samples (#1) collected near a 4 lane street in Copenhagen; urban background samples taken on a roof of a university building in the city	[409]
Greece	1994	urban	3.9	0.7-12.6†	urban location in Athens away from roadways with moderate traffic	[410]
Italy	1994	rural	1.38	NR	sampling along the Mediterranean coastline at a site outside Rome during the day and night	[411]
France	1995-1996	urban	0.78	0.04-25†	sampling in urban center of Douai with heavy traffic nearby	[412]
Norway	1995	remote	0.011	0.008	samples collected on a remote island near the Arctic Ocean	[413]
Greece	1996-1997	remote	0.05	0.00-0.14†	sampling on a forested mountain top at an elevation of 1070 m	[414]
United Kingdom	1996	varied	1.99	1.46	samples collected at 12 urban, residential, and rural background site	[415]

France	1997-2006	rural 1<br>rural 2<br>rural 3	0.137<br>0.100<br>0.147	0.116<br>0.069<br>0.129	sampling in 3 small villages of Donan (#1), Peyrusse-Vieille (#2), and Tardiere (#3)	[416]
France	1997-2000	urban 1<br>urban 2	2.01<br>0.67	0.03-513†<br>0.01-11†	station 1 located in urban center of Lille; station 2 in a residential neighborhood with nearby industry	[417]
France	1997-2000	urban 1<br>urban 2	1.1‡<br>0.45‡	NR<br>NR	sample collection near a busy roadway (#1) and a residential with local industry (#2) in the city of Lille	[418]
France	1997-2000	urban 1<br>urban 2	2.19<br>0.73	NR<br>NR	sample collection near a busy roadway (#1) and a residential with local industry (#2) in the city of Lille	[419]
United Kingdom	1999-2000	urban 1<br>urban 2	1.2<br>0.7	1.2<br>0.8	winter daytime averages for background sites near a university campus car park (#1) and local street (#2)	[420]
Finland	1999	remote 1<br>remote 2	0.072<br>0.030	NR<br>NR	winter (#1) and summer (#2) averages from two remote sites off the coast of Finland in the Baltic Sea or in the subarctic region	[421]

Germany	1999	remote	0.121	NR	mid-day (1:00 PM) average at a mountain observatory (977 m) in Hohenpeissen-berg	[422]
United Kingdom	2000	rural<br>urban 1<br>urban 2<br>roadside	0.23<br>1.10<br>1.28<br>4.18	NR<br>NR<br>NR<br>NR	rural sample collected at a single site outside London; urban 1 samples from 7 cities throughout the UK, urban 2 samples from 3 urban locations with industrial facilities nearby, roadside measurements at the curbside in 2 cities	[423]
Germany	2000	remote	0.076	0.016	afternoon (3:42-3:57 PM) average at a mountain observatory (977 m) in Hohenpeissen-berg	[424]
Spain	2001-2002	urban	2.48	2.86	sampling in a residential area in the city of Vitoria-Gasteiz with heavy traffic nearby	[425]
France	2002-2014	urban 1<br>urban 2<br>suburban	0.43<br>0.71<br>0.48	NR<br>NR<br>NR	multiyear sampling in the urban centers of Paris (#1) and Lyon (#2), and suburban area of Strasbourg	[426]

France	2002-2003	urban	1.07	0.01-104	urban sampling in the city of Dunkerque with heavy industries located nearby	[427]
Ireland	2002	remote<br>airborne	0.006<br>0.012	0.006<br>0.005	sampling at the ground level and at elevations of 390 m (aircraft) at a remote coastal observatory	[428]
Ireland	2003-2004	urban	0.21	0.12	samples collected from a university campus in Belfast near modest traffic density	[429]
Spain	2003-2004	remote	0.09	0.11	site located in the center of a national park	[430]
Greece	2004-2006	remote	0.235	0.068	sampling site on the north coast of Crete	[431]
Norway	2004	remote	0.044	0.017	remote sampling at the foot of a mountain located on an island in the Arctic region	[432]
United Kingdom	2005	airborne	0.019	0.012	samples collected via aircraft over eastern England at 1500 ft elevation	[433]
Switzerland	2005	urban<br>rural	0,66<br>1.05	0,37<br>0.64	winter sampling in background location in the city of Zurich and in the rural village of Roveredo where wood is used for heating	[434]

Portugal	2006	remote 1<br>remote 2	0.05<br>0.02	0.01-8.31†<br>0.01-0.08†	higher altitude sites near a small village in valley (#1) or higher on the mountainside (#2)	[435]
Finland	2006	rural	0.21	NR	winter sampling in the village of Kurkimaki where people heat their homes with wood	[436]
England	2008	urban<br>rural	0.72<br>0.14	NR<br>NR	urban site is near a roadside in central London; rural site is a residential area outside the city	[437]
Germany	2008	remote	0.03	NR	sampling at a remote high altitude location in Germany	[437]
Italy	2011	urban 1<br>urban 2<br>urban 3<br>urban 4	4.05<br>1.65<br>0.95<br>1.43	2.76<br>0.95<br>0.65<br>0.86	samples collected in the winter (#1), spring (#2), summer (#3), and fall (#4) at a site with heavy traffic in the center of Rome	[438]

[a] values reported as ppbC or μg/m^3 were converted to ppbv
unless otherwise stated variability is expressed in term of the standard deviation
^ 5th-95th percentile range for the distribution of values
* 25th-75th percentile range for the distribution of values
∞ 90% confidence interval
≈ 95% confidence interval
† minimum and maximum
Δ maximum value
+ geometric mean
‡ median value
NR not reported

Table S5: Ambient air concentrations of propylene in marine areas

Location	Sampling Date(s)	Monitoring station	Avg. Conc. (ppbv)	Variability[#] (ppbv)	Sampling Details	Reference
Atlantic Ocean	1979	marine 1<br>marine 2	0.12<br>0.11	0.03<br>0.06	samples taken from a research vessel cruising the equatorial Atlantic (#1) or the north Atlantic (#2)	[439]
Atlantic Ocean	1985	airborne 1<br>airborne 2<br>airborne 3	0.038<br>0.033<br>0.023	0.023<br>0.020<br>0.009	samples collected in the lower most boundary layer (#1), the higher free troposphere (#2), and the highest pseudo free troposphere (#3) off the cost of Bermuda	[440]
Pacific and Indian Oceans	1996-1997	marine	0.60	0.03	samples collected aboard the deck of a ship traveling the western North Pacific and eastern Indian Oceans	[441]
Atlantic Ocean	1999	marine 1<br>marine 2<br>marine 3	0.093<br>0.006<br>0.006	0.139<br>0.002<br>0.001	samples categorized according to type of air mass encountered during ship cruise; polluted air mass (#1), oceanic air mass (#2), arctic air mass (#3)	[442]

Indian Ocean	2000	marine	0.035	0.013	sampling aboard a research vessel during a cruise in the northwest regions near the African coast	[443]
Indian Ocean	2002	marine 1<br>marine 2	0.153<br>0.129	0.066<br>0.053	levels recorded during the summer monsoon aboard a ship tracking across the Bay of Bengal (#1) and near coastal areas (#2)	[444]
Indian Ocean	2002	marine	0.046	0.021	sampling during an oceanic cruise campaign in the waters between Africa and Antarctica	[445]
Indian Ocean	2003	marine 1<br>marine 2	0.32<br>0.22	0.08<br>0.04	samples collected during the day (#1) and night (#2) while cruising through the Bay of Bengal	[446]
Gulf of Mexico	2006	marine 1<br>marine 2	0.050<br>1.536	0.160<br>55.58	samples taken from a ship traveling routes through the central Gulf (#1) or through Houston and Galveston Bay (#2)	[447]
Indian Ocean	2006	marine 1<br>marine 2	0.132<br>0.112	0.088<br>0.092	samples taken aboard a research vessel traveling in the Arabian Sea (#1) and the Bay of Bengal (#2)	[448]

Indian Ocean	2010	marine 1<br>marine 2<br>marine 3<br>marine 4	0.059<br>0.062<br>0.071<br>0.067	0.036<br>0.035<br>0.032<br>0.016	cruise through the Bay of Bengal with air masses of marine origin (#1), continental from China (#2), continental from India and Bangladesh (#3), and SE Asia (#4)	[449]

[a] values reported as ppbC or μg/m^3 were converted to ppbv
unless otherwise stated variability is expressed in term of the standard deviation
^ 5th-95th percentile range for the distribution of values
* 25th-75th percentile range for the distribution of values
∞ 90% confidence interval
≈ 95% confidence interval
† minimum and maximum
Δ maximum value
+ geometric mean
‡ median value
NR not reported

Table S6: Ambient air concentrations of propylene in other locations

Location	Sampling Date(s)	Monitoring station	Avg. Conc. (ppbv)	Variability# (ppbv)	Sampling Details	Reference
Australia	1979-1980	urban	7.4	NR	averages from three monitoring sites in the city of Sydney downwind from a refinery and the business district	[450]
Canary Islands	1990-1995	remote 1<br>remote 2	0.0036<br>0.0050	0.0018<br>0.0045	winter (#1) and summer (#2) measurements on a remote island location on the rim of crater at an altitude of 2370 m	[451]
South Africa	1997	remote	0.072	0.024	sampling site located on the coast where it receives clean maritime air	[452]
Tasmania	1999	remote 1<br>remote 2	0.005<br>0.002	NR<br>NR	coastal measurements during the day (#1) and night (#2) atop a cliff (100 m) receiving southern oceanic air	[453]
Antarctica	2004-2005	remote 1<br>remote 2	0.0101<br>0.0093	0.0323<br>0.0110	summer (#1) and winter (#2) samples from a research station on the Brunt ice shelf	[454]

[a] values reported as ppbC or μg/m^3 were converted to ppbv

unless otherwise stated variability is expressed in term of the standard deviation

^ 5th-95th percentile range for the distribution of values

* 25th-75th percentile range for the distribution of values

∞ 90% confidence interval
≈ 95% confidence interval
† minimum and maximum
Δ maximum value
+ geometric mean
‡ median value
NR not reported

References

250. Uno, I.; Wakamatsu, S.; Wadden, R.A.; Konno, S.; Koshio, H. Evaluation of Hydrocarbon Reactivity in Urban Air. *Atmos. Environ.* **1985**, *19*, 1283-1293, doi:org/10.1016/0004-6981(85)90259-8.

251. Khalil, M.A.K.; Rasmussen, R.A.; Wang, M.X.; Ren, L. Emissions of Trace Gases From Chinese Rice Fields and Biogas Generators - CH4, N2O, CO, CO2, Chlorocarbons, and Hydrocarbons. *Chemosphere* **1990**, *20*, 207-226, doi:org/10.1016/0045-6535(90)90097-D.

252. Tanaka, M.; Kamiura, T.; Warashina, M.; Miyazaki, T.; Uno, I.; Wakamatsu, S. Comparison of 16 Hydrocarbon Concentrations Calculated by Receptor Model and Measured Values in Osaka City. *Environ. Sci.* **1994**, *7*, 21-34, doi:org/10.11353/sesj1988.7.

253. Tanaka, M.; Warashina, M.; Itano, Y.; Tsujimoto, Y.; Wakamatsu, S. Effects of Super-Light-Duty Gasoline and LPG-Fueled Cars on 16 Ambient Hrdrocarbons at Roadsides in Japan. *Chemosphere* **2001**, *3*, 199-207, doi:org/10.1016/S1465-9972(00)00051-9.

254. Morikawa, T.; Wakamatsu, S.; Tanaka, M.; Uno, I.; Kamiura, T.; Maeda, T. C2-C5 Hydrocarbon Concentrations in Central Osaka. *Atmos. Environ.* **1998**, *32*, 2007-2016, doi:org/10.1016/S1352-2310(97)00509-8.

255. Rao, A.M.M.; Pandit, G.G.; Sain, P.; Sharma, S.; Krishnamoorthy, T.M.; Nambi, K.S.V. Non-Methane Hydrocarbons in Industrial Locations of Bombay. *Atmos. Environ.* **1997**, *31*, 1077-1085, doi:org/10.1016/S1352-2310(96)00266-X.

256. Na, K.; Kim, Y.P.; Moon, K.C.; Moon, I.; Fung, K. Concentrations of Volatile Organic Compounds in an Industrial Area of Korea. *Atmos. Environ.* **2001**, *35*, 2747-2756, doi:org/10.1016/S1352-2310(00)00313-7.

257. Ding, W.H.; Wang, J.L. Spatial Concentration Profiles of C2-C6 Hydrocarbons in the Atmosphere of Taipei Metropolitan Area. *Chemosphere* **1998**, *37*, 1187-1195, doi:org/10.1016/S0045-6535(98)00113-1.

258. Sharma, U.K.; Kajii, Y.; Akimoto, H. Measurement of NMHCs at Oki Island, Japan,: An Evidence of Long Range Transport. *Geophys. Res. Lett.* **2000**, *27*, 2505-2508, doi:10.1029/2000GL011500.

259. Na, K.; Kim, Y.P. Seasonal Characteristics of Ambient Volatile Organic Compounds in Seoul, Korea. *Atmos. Environ.* **2001**, *35*, 2603-2614, doi:org/10.1016/S1352-2310(00)00464-7.

260. Barletta, B.; Meinardi, S.; Simpson, I.J.; Khwaja, H.A.; Blake, D.R.; Rowland, F.S. Mixing Ratios of Volatile Organic Compounds (VOCs) in the Atmosphere of Karachi, Pakistan. *Atmos. Environ.* **2002**, *36*, 3429-3443, doi:org/10.1016/S1352-2310(02)00302-3.

261. Hsieh, C.C.; Tsai, J.H. VOC Concentration Characteristics in Southern Taiwan. *Chemosphere* **2003**, *50*, 545-556, doi:org/10.1016/S0045-6535(02)00275-8.

262. Sharma, U.K.; Kajii, Y.; Akimoto, H. Seasonal Variation of C2-C6 NMHCs at Happo, a Remote Site in Japan. *Atmos. Environ.* **2000**, *34*, 4447-4458, doi:org/10.1016/S1352-2310(00)00162-X.

263. Sharma, U.K.; Kajii, Y.; Akimoto, H. Characterization of NMHCs in Downtown Urban Center Kathmandu and Rural Site Nagarkot in Nepal. *Atmos. Environ.* **2000**, *34*, 3297-3307, doi:org/10.1016/S1352-2310(99)00485-9.

264. Guo, H.; Wang, T.; Simpson, I.J.; Blake, D.R.; Yu, X.M.; Kwok, Y.H.; Li, Y.S. Source Contributions to Ambient VOCs and CO at a Rural Site in Eastern China. *Atmos. Environ.* **2004**, *38*, 4551-4560, doi:org/10.1016/j.atmosenv.2004.05.004.

265. So, K.L.; Wang, T. C3-C12 Non-Methane Hydrocarbons in Subtropical Hong Kong: Spatial-Temporal Variations, Source-Receptor Relationships and Photochemical Reactivity. *Sci. Total Environ.* **2004**, *328*, 161-174, doi:org/10.1016/j.scitotenv.2004.01.029.

266. Sin, D.W.M.; Wong, Y.C.; Sham, W.C.; Wang, D. Development of an Analytical Technique and Stability Evaluation of 143 C3-C12 Volatile Organic Compounds in Summa(R) Canisters by Gas Chromatography-Mass Spectrometry. *Analyst* **2001**, *126*, 310-321, doi:10.1039/B008746G.

267. Chan, L.Y.; Chu, K.W.; Zou, S.C.; Chan, C.Y.; Wang, X.M.; Barletta, B.; Blake, D.R.; Guo, H.; Tsai, W.Y. Characteristics of Nonmethane Hydrocarbons (NMHCs) in Industrial, Industrial-Urban, and Industrial-Suburban Atmospheres of the Pearl River Delta (PRD) Region of South China. *J. Geochem. Res. Atmos.* **2006**, *111*, doi:10.1029/2005JD006481.

268. Kato, S.; Kajii, Y.; Itokazu, R.; Hirokawa, J.; Koda, S.; Kinjo, Y. Transport of Atmospheric Carbon Monoxide, Ozone, and Hydrocarbons From Chinese Coast to Okinawa Island in the Western Pacific During Winter. *Atmos. Environ.* **2004**, *38*, 2975-2981.

269. Chang, C.C.; Sree, U.; Lin, Y.S.; Lo, J.G. An Examination of 7:00-9:00 PM Ambient Air Volatile Organics in Different Seasons of Kaohsiung City, Southern Taiwan. *Atmos. Environ.* **2005**, *39*, 867-884, doi:org/10.1016/j.atmosenv.2004.10.037.

270. Guo, H.; Wang, T.; Blake, D.R.; Simpson, I.J.; Kwok, Y.H.; Li, Y.S. Regional and Local Contributions to Ambient Non-Methane Volatile Organic Compounds at a Polluted Rural/Coastal Site in Pearl River Delta, China. *Atmos. Environ.* **2006**, *40*, 2345-2359, doi:org/10.1016/j.atmosenv.2005.12.011.

271. Wang, T.; Guo, H.; Blake, D.R.; Kwok, Y.H.; Simpson, I.J.; Li, Y.S. Measurements of Trace Gases in the Inflow of South China Sea Background Air and Outflow of Regional Pollution at Tai O, Southern China. *J. Atmos. Chem.* **2005**, *52*, 295-317, doi:10.1007/s10874-005-2219-x.

272. Barletta, B.; Meinardi, S.; Rowland, F.S.; Chan, C.Y.; Wang, X.M.; Zou, S.C.; Chan, L.Y.; Blake, D.R. Volatile Organic Compounds in 43 Chinese Cities. *Atmos. Environ.* **2005**, *39*, 5979-5990, doi:org/10.1016/j.atmosenv.2005.06.029.

273. Wang, T.; Ding, A.J.; Blake, D.R.; Zahorowski, W.; Poon, C.N.; Li, Y.S. Chemical Characterization of the Boundary Layer Outflow of Air Pollution to Hong Kong During February-April 2001. *J. Geochem. Res. Atmos.* **2003**, *108*, doi:10.1029/2002JD003272.

274. Guo, H.; So, K.L.; Simpson, I.J.; Barletta, B.; Meinardi, S.; Blake, D.R. C1-C8 Volatile Organic Compounds in the Atmosphere of Hong Kong: Overview of Atmospheric Processing and Source Apportionment. *Atmos. Environ.* **2007**, *41*, 1456-1472, doi:org/10.1016/j.atmosenv.2006.10.011.

275. Sahu, L.K.; Lal, S. Distributions of C2-C5 NMHCs and Related Trace Gases at a Tropical Urban Site in India. *Atmos. Environ.* **2006**, *40*, 880-891, doi:org/10.1016/j.atmosenv.2005.10.021.

276. Sahu, L.K.; Lal, S. Characterization of C2-C4 NMHCs Distributions at a High Altitude Tropical Site in India. *J. Atmos. Chem.* **2006**, *54*, 161-175, doi:10.1007/s10874-006-9023-0.

277. Chang, C.C.; Chen, T.Y.; Lin, C.Y.; Yuan, C.S.; Liu, S.C. Effects of Reactive Hydrocarbons on Ozone Formation in Southern Taiwan. *Atmos. Environ.* **2005**, *39*, 2867-2878, doi:org/10.1016/j.atmosenv.2004.12.042.

278. Tsai, D.H.; Wang, J.L.; Chuang, K.J.; Chan, C.C. Traffic-Related Air Pollution and Cardiovascular Mortality in Central Taiwan. *Sci. Total Environ.* **2010**, *408*, 1818-1823, doi:org/10.1016/j.scitotenv.2010.01.044.

279. Saito, S.; Nagao, I.; Kanzawa, H. Characteristics of Ambient C2-C11 Non-Methane Hydrocarbons in Metropolitan Nagoya, Japan. *Atmos. Environ.* **2009**, *43,* 4384-4395, doi:org/10.1016/j.atmosenv.2009.04.031.

280. Lai, C.H.; Chen, K.S.; Ho, Y.T.; Chou, M.S. Characteristics of C2-C15 Hydrocarbons in the Air of Urban Kaohsiung, Taiwan. *Atmos. Environ.* **2004**, *38,* 1997-2011, doi:org/10.1016/j.atmosenv.2003.11.041.

281. Shin, H.J.; Roh, S.A.; Kim, J.C.; Lee, S.J.; Kim, Y.P. Temporal Variation of Volatile Organic Compounds and Their Major Emission Sources in Seoul, Korea. *Environ. Sci. Pollut. Res.* **2013**, *20,* 8717-8728, doi:10.1007/s11356-013-1843-2.

282. Tang, J.H.; Chan, L.Y.; Chan, C.Y.; Li, Y.S.; Chang, C.C.; Liu, S.C.; Li, Y.D. Nonmethane Hydrocarbons in the Transported and Local Air Masses at a Clean Remote Site on Hainan Island, South China. *J. Geochem. Res. Atmos.* **2007**, *112,* doi:10.1029/2006JD007796.

283. Tang, J.H.; Chan, L.Y.; Chang, C.C.; Liu, S.; Li, Y.S. Characteristics and Sources of Non-Methane Hydrocarbons in Background Atmospheres of Eastern, Southwestern, and Southern China. *J. Geochem. Res. Atmos.* **2009**, *114,* doi:10.1029/2008JD010333.

284. Lal, S.; Sahu, L.K.; Venkataramani, S.; Rajesh, T.A.; Modh, K.S. Distributions of O3, CO and NMHCs Over the Rural Sites in Central India. *J. Atmos. Chem.* **2008**, *61,* 73-84, doi:10.1007/s10874-009-9126-5.

285. Lal, S.; Sahu, L.K.; Venkataramani, S.; Mallik, C. Light Non-Methane Hydrocarbons at Two Sites in the Indo-Gangetic Plain. *J. Environ. Monit.* **2012**, *14,* 1159-1166, doi:10.1039/C2EM10682E.

286. Shirai, T.; Yokouchi, Y.; Blake, D.R.; Kita, K.; Izumi, K.; Koike, M.; Komazaki, Y.; Miyazaki, Y.; Fukuda, M.; Kondo, Y. Seasonal Variations of Atmospheric C2-C7 Nonmethane Hydrocarbons in Tokyo. *J. Geochem. Res. Atmos.* **2007**, *112*.

287. Nguyen, H.T.; Kim, K.H.; Kim, M.Y. Volatile Organic Compounds at an Urban Monitoring Station in Korea. *J. Hazard. Mater.* **2009**, *161,* 163-174, doi:org/10.1016/j.jhazmat.2008.03.066.

288. Chiang, H.L.; Tsai, J.H.; Chen, S.Y.; Lin, K.H.; Ma, S.Y. VOC Concentration Profiles in an Ozone Non-Attainment Area: A Case Study in an Urban and Industrial Complex Metroplex in Southern Taiwan. *Atmos. Environ.* **2007**, *41,* 1848-1860, doi:org/10.1016/j.atmosenv.2006.10.055.

289. Barletta, B.; Meinardi, S.; Simpson, I.J.; Zou, S.C.; Rowland, F.S.; Blake, D.R. Ambient Mixing Ratios of Nonmethane Hydrocarbons (NMHCs) in Two Major Urban Centers of the Pearl River Delta (PRD) Region: Guangzhou and Dongguan. *Atmos. Environ.* **2008**, *42,* 4393-4408, doi:org/10.1016/j.atmosenv.2008.01.028.

290. Li, L.F.; Wang, X.M. Seasonal and Diurnal Variations of Atmospheric Non-Methane Hydrocarbons in Guangzhou, China. *Int. J. Environ. Res. Public Health* **2012**, *9,* 1859-1873, doi:10.3390/ijerph9051859.

291. Tang, J.H.; Chan, L.Y.; Chan, C.Y.; Li, Y.S.; Chang, C.C.; Liu, S.C.; Wu, D.; Li, Y.D. Characteristics and Diurnal Variations of NMHCs at Urban, Suburban, and Rural Sites in the Pearl River Delta and a Remote Site in South China. *Atmos. Environ.* **2007**, *41,* 8620-8632, doi:org/10.1016/j.atmosenv.2007.07.029.

292. Song, Y.; Shao, M.; Liu, Y.; Lu, S.H.; Kuster, W.; Goldan, P.; Xie, S.D. Source Apportionment of Ambient Volatile Organic Compounds in Beijing. *Environ. Sci. Technol.* **2007**, *41,* 4348-4353, doi:10.1021/es0625982.

293. Song, P.; Chan, C.Y.; Geng, F.H.; Yu, Q.; Guo, Y.F.; Yu, L.W. Elevated Mixing Ratios and Sources of Methyl Chloride: Results From a Survey in the Yangtze River Delta Region of China. *Atmos. Res.* **2012**, *104,* 172-181, doi:org/10.1016/j.atmosres.2011.10.003.

294. Song, P.; Geng, F.H.; Sang, X.F.; Chan, C.Y.; Chan, L.Y.; Yu, Q. Characteristics and Sources of Non-Methane Hydrocarbons and Halocarbons in Wintertime Urban Atmosphere of Shanghai, China. *Environ. Monit. Assess.* **2012**, *184*, 5957-5970, doi:10.1007/s10661-011-2393-z.

295. Sharma, S.; Giri, B.; Patel, K.S. Ambient Volatile Organic Compounds in the Atmosphere of Industrial Central India. *J. Atmos. Chem.* **2016**, *73*, 381-395, doi:10.1007/s10874-016-9329-5.

296. Ran, L.; Zhao, C.S.; Geng, F.H.; Tie, X.X.; Tang, X.; Peng, L.; Zhou, G.Q.; Yu, Q.; Xu, J.M.; Guenther, A. Ozone Photochemical Production in Urban Shanghai, China: Analysis Based on Ground Level Observations. *J. Geochem. Res. Atmos.* **2009**, *114*, doi:10.1029/2008JD010752.

297. Geng, F.H.; Cai, C.J.; Tie, X.X.; Yu, Q.; An, J.L.; Peng, L.; Zhou, G.Q.; Xu, J.M. Analysis of VOC Emissions Using PCA/APCS Receptor Model at City of Shanghai, China. *J. Atmos. Chem.* **2009**, *62*, 229-247, doi:10.1007/s10874-010-9150-5.

298. Duan, J.C.; Tan, J.H.; Yang, L.; Wu, S.; Hao, J.M. Concentration, Sources and Ozone Formation Potential of Volatile Organic Compounds (VOCs) During Ozone Episode in Beijing. *Atmos. Res.* **2008**, *88*, 25-35, doi:org/10.1016/j.atmosres.2007.09.004.

299. Xie, X.; Shao, M.; Liu, Y.; Lu, S.H.; Chang, C.C.; Chen, Z.M. Estimate of Initial Isoprene Contribution to Ozone Formation Potential in Beijing, China. *Atmos. Environ.* **2008**, *42*, 6000-6010.

300. Suthawaree, J.; Kato, S.; Okuzawa, K.; Kanaya, Y.; Pochanart, P.; Akimoto, H.; Wang, Z.; Kajii, Y. Measurements of Volatile Organic Compounds in the Middle of Central East China During Mount Tai Experiment 2006 (MTX2006): Observation of Regional Background and Impact of Biomass Burning. *Atmos. Chem. Phys.* **2010**, *10*, 1269-1285, doi:org/10.5194/acp-10-1269-2010.

301. Guo, S.J.; Tan, J.H.; Duan, J.C.; Ma, Y.L.; Yang, F.M.; He, K.B.; Hao, J.M. Characteristics of Atmospheric Non-Methane Hydrocarbons During Haze Episode in Beijing, China. *Environ. Monit. Assess.* **2012**, *184*, 7235-7246.

302. Tiwari, V.; Hanai, Y.; Masunaga, S. Ambient Levels of Volatile Organic Compounds in the Vicinity of Petrochemical Industrial Area of Yokohama, Japan. *Air Qual. Atmos. Health* **2010**, *3*, 65-75, doi:10.1007/s11869-009-0052-0.

303. Chen, S.P.; Liao, W.C.; Chang, C.C.; Su, Y.C.; Tong, Y.H.; Chang, J.S.; Wang, J.L. Network Monitoring of Speciated Vs. Total Non-Methane Hydrocarbon Measurements. *Atmos. Environ.* **2014**, *90*, 33-42, doi:org/10.1016/j.atmosenv.2014.03.020.

304. Cai, C.J.; Geng, F.H.; Tie, X.X.; Yu, Q.O.; An, J.L. Characteristics and Source Apportionment of VOCs Measured in Shanghai, China. *Atmos. Environ.* **2010**, *44*, 5005-5014, doi:org/10.1016/j.atmosenv.2010.07.059.

305. Suthawaree, J.; Kato, S.; Pochanart, P.; Kanaya, Y.; Akimoto, H.; Wang, Z.F.; Kajii, Y. Influence of Beijing Outflow on Volatile Organic Compounds (VOC) Observed at a Mountain Site in North China Plain. *Atmos. Res.* **2012**, *111*, 46-57, doi:org/10.1016/j.atmosres.2012.02.016.

306. Ling, Z.H.; Guo, H.; Cheng, H.R.; Yu, Y.F. Sources of Ambient Volatile Organic Compounds and Their Contributions to Photochemical Ozone Formation at a Site in the Pearl River Delta, Southern China. *Environ. Pollut.* **2011**, *159*, 2310-2319, doi:org/10.1016/j.envpol.2011.05.001.

307. Zhang, J.G.; Wang, Y.S.; Wu, F.K.; Lin, H.; Wang, W.D. Nonmethane Hydrocarbon Measurements at a Suburban Site in Changsha City, China. *Sci. Total Environ.* **2009**, *408*, 312-317, doi:org/10.1016/j.scitotenv.2009.07.010.

308. Xue, L.K.; Wang, T.; Simpson, I.J.; Ding, A.J.; Gao, J.; Blake, D.R.; Wang, X.Z.; Wang, W.X.; Lei, H.C.; Jing, D.Z. Vertical Distributions of Non-Methane Hydrocarbons and Halocarbons in the Lower Troposphere Over Northeast China. *Atmos. Environ.* **2011**, *45*, 6501-6509, doi:org/10.1016/j.atmosenv.2011.08.072.

309. Guo, S.J.; Tan, J.H.; Ma, Y.L.; Yang, F.M.; Yu, Y.C.; Wang, J.W. Characteristics of Atmospheric Non-Methane Hydrocarbons During High PM10 Episodes and Normal Days in Foshan, China. *Atmos. Res.* **2011**, *101*, 701-710, doi:org/10.1016/j.atmosres.2011.04.022.

310. Tan, J.H.; Guo, S.J.; Ma, Y.L.; Yang, F.M.; He, K.B.; Yu, Y.C.; Wang, J.W.; Shi, Z.B.; Chen, G.C. Non-Methane Hydrocarbons and Their Ozone Formation Potentials in Foshan, China. *Aerosol Air Qual. Res.* **2012**, *12*, 387-398, doi:10.4209/aaqr.2011.08.0127.

311. Xu, J.; Ma, J.Z.; Zhang, X.L.; Xu, X.B.; Xu, X.F.; Lin, W.L.; Wang, Y.; Meng, W.; Ma, Z.Q. Measurements of Ozone and Its Precursors in Beijing During Summertime: Impact of Urban Plumes on Ozone Pollution in Downwind Rural Areas. *Atmos. Chem. Phys.* **2011**, *11*, 12241-12252, doi:org/10.5194/acp-11-12241-2011.

312. Wang, B.; Shao, M.; Lu, S.H.; Yuan, B.; Zhao, Y.; Wang, M.; Zhang, S.Q.; Wu, D. Variation of Ambient Non-Methane Hydrocarbons in Beijing City in Summer 2008. *Atmos. Chem. Phys.* **2010**, *10*, 5911-5923, doi:10.5194/acp-10-5911-2010.

313. An, J.L.; Wang, Y.S.; Wu, F.K.; Zhu, B. Characterizations of Volatile Organic Compounds During High Ozone Episodes in Beijing, China. *Environ. Monit. Assess.* **2012**, *184*, 1879-1889, doi:10.1007/s10661-011-2086-7.

314. Lin, C.C.; Lin, C.; Hsieh, L.T.; Chen, C.Y.; Wang, J.P. Vertical and Diurnal Characterization of Volatile Organic Compounds in Ambient Air in Urban Areas. *J. Air Waste Manage. Assoc.* **2011**, *61*, 714-720, doi:org/10.3155/1047-3289.61.7.714.

315. Suthawaree, J.; Tajima, Y.; Khunchornyakong, A.; Kato, S.; Sharp, A.; Kajii, Y. Identification of Volatile Organic Compounds in Suburban Bangkok, Thailand and Their Potential for Ozone Formation. *Atmos. Res.* **2012**, *104*, 245-254, doi:org/10.1016/j.atmosres.2011.10.019.

316. Sarangi, T.; Naja, M.; Lal, S.; Venkataramani, S.; Bhardwaj, P.; Ojha, N.; Kumar, R.; Chandola, H.C. First Observations of Light Non-Methane Hydrocarbons (C2-C5) Over a High Altitude Site in the Central Himalayas. *Atmos. Environ.* **2016**, *125*, 450-460, doi:org/10.1016/j.atmosenv.2015.10.024.

317. Wang, N.; Li, N.; Liu, Z.C.; Evans, E. Investigation of Chemical Reactivity and Active Components of Ambient VOCs in Jinan, China. *Air Qual. Atmos. Health* **2016**, *9*, 785-793, doi:10.1007/s11869-015-0380-1.

318. Wang, J.L.; Chew, C.; Chang, C.Y.; Liao, W.C.; Lung, S.C.C.; Chen, W.N.; Lee, P.J.; Lin, P.H.; Chang, C.C. Biogenic Isoprene in Subtropical Urban Settings and Implications for Air Quality. *Atmos. Environ.* **2013**, *79*, 369-379, doi:org/10.1016/j.atmosenv.2013.06.055.

319. Huang, Y.; Ling, Z.H.; Lee, S.C.; Ho, S.S.H.; Cao, J.J.; Blake, D.R.; Cheng, Y.; Lai, S.C.; Ho, K.F.; Gao, Y.; Cui, L.; Louie, P.K.K. Characterization of Volatile Organic Compounds at a Roadside Environment in Hong Kong: An Investigation of Influences After Air Pollution Control Strategies. *Atmos. Environ.* **2015**, *122*, 809-818, doi:org/10.1016/j.atmosenv.2015.09.036.

320. Suthawaree, J.; Sikder, H.A.; Jones, C.E.; Kato, S.; Kunimi, H.; Kabir, A.M.H.; Kajii, Y. Influence of Extensive Compressed Natural Gas (CNG) Usage on Air Quality. *Atmos. Environ.* **2012**, *54*, 296-307, doi:org/10.1016/j.atmosenv.2012.01.066.

321. Yuan, B.; Hu, W.W.; Shao, M.; Wang, M.; Chen, W.T.; Lu, S.H.; Zeng, L.M.; Hu, M. VOC Emissions, Evolutions and Contributions to SOA Formation at a Receptor Site in Eastern China. *Atmos. Chem. Phys.* **2013**, *13*, 8815-8832, doi:10.5194/acp-13-8815-2013.

322. Sarkar, C.; Sinha, V.; Kumar, V.; Rupakheti, M.; Panday, A.; Mahata, K.S.; Rupakheti, D.; Kathayat, B.; Lawrence, M.G. Overview of VOC Emissions and Chemistry From PTR-TOF-MS Measurements During the SusKat-ABC Campaign: High Acetaldehyde, Isoprene and Isocyanic Acid in Wintertime Air of the Kathmandu Valley. *Atmos. Chem. Phys.* **2016**, *16*, 3979-4003, doi:10.5194/acp-16-3979-2016.

323. Barletta, B.; Simpson, I.J.; Blake, N.J.; Meinardi, S.; Emmons, L.K.; Aburizaiza, O.S.; Siddique, A.; Zeb, J.; Yu, L.E.; Khwaja, H.A.; Farrukh, M.A.; Blake, D.R. Characterization of Carbon Monoxide, Methane and Nonmethane Hydrocarbons in Emerging Cities of Saudi Arabia and Pakistan and in Singapore. *J. Atmos. Chem.* **2016**, 1-27, doi:10.1007/s10874-016-9343-7.

324. Li, L.Y.; Chen, Y.; Zeng, L.M.; Shao, M.; Xie, S.D.; Chen, W.T.; Lu, S.H.; Wu, Y.S.; Cao, W. Biomass Burning Contribution to Ambient Volatile Organic Compounds (VOCs) in the Chengdu-Chongqing Region (CCR), China. *Atmos. Environ.* **2014**, *99*, 403-410, doi:org/10.1016/j.atmosenv.2014.09.067.

325. Mallik, C.; Ghosh, D.; Ghosh, D.; Sarkar, U.; Lal, S.; Venkataramani, S. Variability of SO2, CO, and Light Hydrocarbons Over a Megacity in Eastern India: Effects of Emissions and Transport. *Environ. Sci. Pollut. Res.* **2014**, *21*, 8692-8706, doi:10.1007/s11356-014-2795-x.

326. Kuo, C.P.; Liao, H.T.; Chou, C.C.K.; Wu, C.F. Source Apportionment of Particulate Matter and Selected Volatile Organic Compounds With Multiple Time Resolution Data. *Sci. Total Environ.* **2014**, *472*, 880-887, doi:org/10.1016/j.scitotenv.2013.11.114.

327. Zhu, Y.H.; Yang, L.X.; Chen, J.M.; Wang, X.F.; Xue, L.K.; Sui, X.; Wen, L.; Xu, C.H.; Yao, L.; Zhang, J.M.; Shao, M.; Lu, S.H.; Wang, W.X. Characteristics of Ambient Volatile Organic Compounds and the Influence of Biomass Burning at a Rural Site in Northern China During Summer 2013. *Atmos. Environ.* **2016**, *124*, 156-165, doi:org/10.1016/j.atmosenv.2015.08.097.

328. Lyu, X.P.; Liu, M.; Guo, H.; Ling, Z.H.; Wang, Y.; Louie, P.K.K.; Luk, C.W.Y. Spatiotemporal Variation of Ozone Precursors and Ozone Formation in Hong Kong: Grid Field Measurement and Modelling Study. *Sci. Total Environ.* **2016**, *569*, 1341-1349, doi:org/10.1016/j.scitotenv.2016.06.214.

329. Shao, P.; An, J.L.; Xin, J.Y.; Wu, F.K.; Wang, J.X.; Ji, D.S.; Wang, Y.S. Source Apportionment of VOCs and the Contribution to Photochemical Ozone Formation During Summer in the Typical Industrial Area in the Yangtze River Delta, China. *Atmos. Res.* **2016**, *176*, 64-74, doi:org/10.1016/j.atmosres.2016.02.015.

330. Jia, C.H.; Mao, X.X.; Huang, T.; Liang, X.X.; Wang, Y.N.; Shen, Y.J.; Jiang, W.Y.H.; Wang, H.Q.; Bai, Z.L.; Ma, M.Q.; Yu, Z.S.; Ma, J.M.; Gao, H. Non-Methane Hydrocarbons (NMHCs) and Their Contribution to Ozone Formation Potential in a Petrochemical Industrialized City, Northwest China. *Atmos. Res.* **2016**, *169*, 225-236, doi:org/10.1016/j.atmosres.2015.10.006.

331. Li, L.Y.; Xie, S.D.; Zeng, L.M.; Wu, R.R.; Li, J. Characteristics of Volatile Organic Compounds and Their Role in Ground-Level Ozone Formation in the Beijing-Tianjin-Hebei Region, China. *Atmos. Environ.* **2015**, *113*, 247-254, doi:org/10.1016/j.atmosenv.2015.05.021.

332. Venkanna, R.; Nikhil, G.N.; Sinha, P.R.; Rao, T.S.; Swamy, Y.V. Significance of Volatile Organic Compounds and Oxides of Nitrogen on Surface Ozone Formation at Semi-Arid Tropical Urban Site, Hyderabad, India. *Air Qual. Atmos. Health* **2016**, *9*, 379-390, doi:10.1007/s11869-015-0347-2.

333. Wang, G.; Cheng, S.Y.; Wei, W.; Zhou, Y.; Yao, S.; Zhang, H.Y. Characteristics and Source Apportionment of VOCs in the Suburban Area of Beijing, China. *Atmos. Pollut. Res.* **2016**, *7*, 711-724, doi:org/10.1016/j.apr.2016.03.006.

334. Sexton, K.; Westberg, H. Nonmethane Hydrocarbon Composition of Urban and Rural Atmospheres. *Atmos. Environ.* **1984**, *18*, 1125-1132, doi:org/10.1016/0004-6981(84)90144-6.

335. Arnts, R.R.; Meeks, S.A. Biogenic Hydrocarbon Contribution to the Ambient Air of Selected Areas. *Atmos. Environ.* **1981**, *15*, 1643-1651, doi:org/10.1016/0004-6981(81)90149-9.

336. Chameides, W.L.; Fehsenfeld, F.; Rodgers, M.O.; Cardelino, C.; Martinez, J.; Parrish, D.; Lonneman, W.; Lawson, D.R.; Rasmussen, R.A.; Zimmerman, P.; Greenberg, J.; Middleton, P.; Wang, T. Ozone Precursor Relationships in the Ambient Atmosphere. *J. Geochem. Res. Atmos.* **1992**, *97*, 6037-6055, doi:10.1029/91JD03014.

337. Seila, R. L., Lonneman, W. A., and Meeks, S. A. Determination of C2 to C12 Ambient Air Hydrocarbons in 39 U.S. Cities, from 1984 through 1986. Available online: https://nepis.epa.gov/Exe/ZyPDF.cgi/2000TIPL.PDF?Dockey=2000TIPL.PDF. (accessed on 20 April 2016).

338. Aronian, P.F.; Scheff, P.A.; Wadden, R.A. Wintertime Source Reconciliation of Ambient Organics. *Atmos. Environ.* **1989**, *23*, 911-920, doi:org/10.1016/0004-6981(89)90295-3.

339. Doskey, P.V.; Gaffney, J.S. Nonmethane Hydrocarbons in the Arctic Atmosphere at Barrow, Alaska. *Geophys. Res. Lett.* **1992**, *19*, 381-384, doi:10.1029/91GL03136.

340. Spicer, C.W.; Buxton, B.E.; Holdren, M.W.; Smith, D.L.; Kelly, T.J.; Rust, S.W.; Pate, A.D.; Sverdrup, G.M.; Chuang, J.C. Variability of Hazardous Air Pollutants in an Urban Area. *Atmos. Environ.* **1996**, *30*, 3443-3456, doi:org/10.1016/1352-2310(95)00200-6.

341. Greenberg, J.P.; Helmig, D.; Zimmerman, P.R. Seasonal Measurements of Nonmethane Hydrocarbons and Carbon Monoxide at the Mauna Loa Observatory During the Mauna Loa Observatory Photochemistry Experiment 2. *J. Geochem. Res. Atmos.* **1996**, *101*, 14581-14598, doi:10.1029/95JD01543.

342. Cheng, L.; Fu, L.; Angle, R.P.; Sandhu, H.S. Seasonal Variations of Volatile Organic Compounds in Edmonton, Alberta. *Atmos. Environ.* **1997**, *31*, 239-246, doi:org/10.1016/1352-2310(96)00170-7.

343. Bottenheim, J.W.; Shepherd, M.F. C2-C6 Hydrocarbon Measurements at Four Rural Locations Across Canada. *Atmos. Environ.* **1995**, *29*, 647-664, doi:org/10.1016/1352-2310(94)00318-F.

344. Hagerman, L.M.; Aneja, V.P.; Lonneman, W.A. Characterization of Non-Methane Hydrocarbons in the Rural Southeast United States. *Atmos. Environ.* **1997**, *31*, 4017-4038, doi:org/10.1016/S1352-2310(97)00223-9.

345. Ellenson, W.D.; Mukerjee, S.; Stevens, R.K.; Willis, R.D.; Shadwick, D.S.; Somerville, M.C.; Lewis, R.G. An Environmental Scoping Study in the Lower Rio Grande Valley of Texas .2. Assessment of Transboundary Pollution Transport and Other Activities by Air Quality Monitoring. *Environ. Int.* **1997**, *23*, 643-655, doi:org/10.1016/S0160-4120(97)00067-6.

346. Gong, Q.; Demerjian, K.L. Measurement and Analysis of C2-C10 Hydrocarbons at Whiteface Mountain, New York. *J. Geochem. Res. Atmos.* **1997**, *102*, 28059-28069, doi:10.1029/97JD02703.

347. Khwaja, H.A.; Narang, A. Carbonyls and Non-Methane Hydrocarbons at a Rural Mountain Site in Northeastern United States. *Chemosphere* **2008**, *71*, 2030-2043, doi:org/10.1016/j.chemosphere.2008.01.042.

348. Kang, D.W.; Aneja, V.P.; Zika, R.G.; Farmer, C.; Ray, J.D. Nonmethane Hydrocarbons in the Rural Southeast United States National Parks. *J. Geochem. Res. Atmos.* **2001**, *106,* 3133-3155, doi:10.1029/2000JD900607.

349. Kang, D.; Aneja, V.P.; Das, M.; Seila, R. Measurements of Air-Surface Exchange Rates of Volatile Organic Compounds. *Int. J. Environ. Pollut.* **2004**, *22,* 547-562, doi:10.1504/IJEP.2004.005909.

350. Qin, Y.; Walk, T.; Gary, R.; Yao, X.; Elles, S. C2-C10 Nonmethane Hydrocarbons Measured in Dallas, USA - Seasonal Trends and Diurnal Characteristics. *Atmos. Environ.* **2007**, *41,* 6018-6032, doi:org/10.1016/j.atmosenv.2007.03.008.

351. Mohamed, M.F.; Kang, D.W.; Aneja, V.P. Volatile Organic Compounds in Some Urban Locations in United States. *Chemosphere* **2002**, *47,* 863-882, doi:org/10.1016/S0045-6535(02)00107-8.

352. Jaffe, D.; Anderson, T.; Covert, D.; Trost, B.; Danielson, J.; Simpson, W.; Blake, D.; Harris, J.; Streets, D. Observations of Ozone and Related Species in the Northeast Pacific During the PHOBEA Campaigns 1. Ground-Based Observations at Cheeka Peak. *J. Geochem. Res. Atmos.* **2001**, *106,* 7449-7461, doi:10.1029/2000JD900636.

353. Baker, A.K.; Beyersdorf, A.J.; Doezema, L.A.; Katzenstein, A.; Meinardi, S.; Simpson, I.J.; Blake, D.R.; Rowland, F.S. Measurements of Nonmethane Hydrocarbons in 28 United States Cities. *Atmos. Environ.* **2008**, *42,* 170-182, doi:org/10.1016/j.atmosenv.2007.09.007.

354. Pang, Y.B.; Fuentes, M.; Rieger, P. Trends in Selected Ambient Volatile Organic Compound (VOC) Concentrations and a Comparison to Mobile Source Emission Trends in California's South Coast Air Basin. *Atmos. Environ.* **2015**, *122,* 686-695, doi:org/10.1016/j.atmosenv.2015.10.016.

355. Porada, E.; Kousha, T. Factorization Methods Applied to Characterize the Sources of Volatile Organic Compounds in Montreal, Quebec. *Int. J. Occup. Med. Environ. Health* **2016**, *29,* 15-39, doi:org/10.13075/ijomeh.1896.00509.

356. Bottenheim, J.W.; Boudries, H.; Brickell, P.C.; Atlas, E. Alkenes in the Arctic Boundary Layer at Alert, Nunavut, Canada. *Atmos. Environ.* **2002**, *36,* 2585-2594, doi:org/10.1016/S1352-2310(02)00113-9.

357. Jobson, B.T.; Berkowitz, C.M.; Kuster, W.C.; Goldan, P.D.; Williams, E.J.; Fesenfeld, F.C.; Apel, E.C.; Karl, T.; Lonneman, W.A.; Riemer, D. Hydrocarbon Source Signatures in Houston, Texas: Influence of the Petrochemical Industry. *J. Geochem. Res. Atmos.* **2004**, *109,* doi:10.1029/2004JD004887.

358. Olson, D.A.; Norris, G.A.; Seila, R.L.; Landis, M.S.; Vette, A.F. Chemical Characterization of Volatile Organic Compounds Near the World Trade Center: Ambient Concentrations and Source Apportionment. *Atmos. Environ.* **2007**, *41,* 5673-5683, doi:org/10.1016/j.atmosenv.2007.02.047.

359. Myers, J.L.; Phillips, T.; Grant, R.L. Emissions and Ambient Air Monitoring Trends of Lower Olefins Across Texas From 2002 to 2012. *Chem. Biol. Interact.* **2015**, *241,* 2-9, doi:org/10.1016/j.cbi.2015.02.008.

360. Millet, D.B.; Goldstein, A.H.; Allan, J.D.; Bates, T.S.; Boudries, H.; Bower, K.N.; Coe, H.; Ma, Y.L.; Mckay, M.; Quinn, P.K.; Sullivan, A.; Weber, R.J.; Worsnop, D.R. Volatile Organic Compound Measurements at Trinidad Head, California, During ITCT 2K2: Analysis of Sources, Atmospheric Composition, and Aerosol Residence Times. *J. Geochem. Res. Atmos.* **2004**, *109,* doi:10.1029/2003JD004026.

361. Millet, D.B.; Donahue, N.M.; Pandis, S.N.; Polidori, A.; Stanier, C.O.; Turpin, B.J.; Goldstein, A.H. Atmospheric Volatile Organic Compound Measurements During the Pittsburgh Air Quality Study: Results, Interpretation, and Quantification of Primary and Secondary Contributions. *J. Geochem. Res. Atmos.* **2005**, *110,* doi:10.1029/2004JD004601.

362. Buzcu, B.; Fraser, M.P. Source Identification and Apportionment of Volatile Organic Compounds in Houston, TX. *Atmos. Environ.* **2006**, *40*, 2385-2400, doi:org/10.1016/j.atmosenv.2005.12.020.

363. Russo, R.S.; Zhou, Y.; White, M.L.; Mao, H.; Talbot, R.; Sive, B.C. Multi-Year (2004-2008) Record of Nonmethane Hydrocarbons and Halocarbons in New England: Seasonal Variations and Regional Sources. *Atmos. Chem. Phys.* **2010**, *10*, 4909-4929, doi:10.5194/acp-10-4909-2010.

364. Mintz, R.; McWhinney, R.D. Characterization of Volatile Organic Compound Emission Sources in Fort Saskatchewan, Alberta Using Principal Component Analysis. *J. Atmos. Chem.* **2008**, *60*, 83-101, doi:10.1007/s10874-008-9110-5.

365. Holzinger, R.; Millet, D.B.; Williams, B.; Lee, A.; Kreisberg, N.; Hering, S.V.; Jimenez, J.; Allan, J.D.; Worsnop, D.R.; Goldstein, A.H. Emission, Oxidation, and Secondary Organic Aerosol Formation of Volatile Organic Compounds As Observed at Chebogue Point, Nova Scotia. *J. Geochem. Res. Atmos.* **2007**, *112*, doi:10.1029/2006JD007599.

366. Stroud, C.A.; Morneau, G.; Makar, P.A.; Moran, M.D.; Gong, W.; Pabla, B.; Zhang, J.; Bouchet, V.S.; Fox, D.; Venkatesh, S.; Wang, D.; Dann, T. OH-Reactivity of Volatile Organic Compounds at Urban and Rural Sites Across Canada: Evaluation of Air Quality Model Predictions Using Speciated VOC Measurements. *Atmos. Environ.* **2008**, *42*, 7746-7756, doi.org/10.1016/j.atmosenv.2008.05.054.

367. Leuchner, M.; Rappengluck, B. VOC Source-Receptor Relationships in Houston During TexAQS-II. *Atmos. Environ.* **2010**, *44*, 4056-4067, doi:org/10.1016/j.atmosenv.2009.02.029.

368. Doezema, L.A.; Bigley, C.; Canzi, G.; Chang, K.; Hirning, A.J.; Lee, J.; Von der Ahe, N. The Influence of Sampling Protocol on Nonmethane Hydrocarbon Mixing Ratios. *Atmos. Environ.* **2010**, *44*, 900-908, doi:org/10.1016/j.atmosenv.2009.12.007.

369. Gorham, K.A.; Blake, N.J.; VanCuren, R.A.; Fuelberg, H.E.; Meinardi, S.; Blake, D.R. Seasonal and Diurnal Measurements of Carbon Monoxide and Nonmethane Hydrocarbons at Mt. Wilson, California: Indirect Evidence of Atomic Cl in the Los Angeles Basin. *Atmos. Environ.* **2010**, *44*, 2271-2279, doi:org/10.1016/j.atmosenv.2010.04.019.

370. Simpson, I.J.; Blake, N.J.; Barletta, B.; Diskin, G.S.; Fuelberg, H.E.; Gorham, K.; Huey, L.G.; Meinardi, S.; Rowland, F.S.; Vay, S.A.; Weinheimer, A.J.; Yang, M.; Blake, D.R. Characterization of Trace Gases Measured Over Alberta Oil Sands Mining Operations: 76 Speciated C2-C10 Volatile Organic Compounds (VOCs), CO2, CH4, CO, NO, NO2, NOy, O3 and SO2. *Atmos. Chem. Phys.* **2010**, *10*, 11931-11954, doi:10.5194/acp-10-11931-2010.

371. Warneke, C.; de Gouw, J.A.; Holloway, J.S.; Peischl, J.; Ryerson, T.B.; Atlas, E.; Blake, D.; Trainer, M.; Parrish, D.D. Multiyear Trends in Volatile Organic Compounds in Los Angeles, California: Five Decades of Decreasing Emissions. *J. Geochem. Res. Atmos.* **2012**, *117*, doi:10.1029/2012JD017899.

372. Gilman, J.B.; Lerner, B.M.; Kuster, W.C.; de Gouw, J.A. Source Signature of Volatile Organic Compounds From Oil and Natural Gas Operations in Northeastern Colorado. *Environ. Sci. Technol.* **2013**, *47*, 1297-1305, doi:10.1021/es304119a.

373. Swarthout, R.F.; Russo, R.S.; Zhou, Y.; Hart, A.H.; Sive, B.C. Volatile Organic Compound Distributions During the NACHTT Campaign at the Boulder Atmospheric Observatory: Influence of Urban and Natural Gas Sources. *J. Geochem. Res. Atmos.* **2013**, *118*, 10614-10637, doi:10.1002/jgrd.50722.

374. Thompson, C.R.; Hueber, J.; Helmig, D. Influence of Oil and Gas Emissions on Ambient Atmospheric Non-Methane Hydrocarbons in Residential Areas of Northeastern Colorado. *Elementa* **2014**, *3,* 117, doi:org/10.12952/journal.elementa.000035.

375. Donoso, L.; Romero, R.; Rondon, A.; Fernandez, E.; Oyola, P.; Sanhueza, E. Natural and Anthropogenic C2 to C6 Hydrocarbons in the Central-Eastern Venezuelan Atmosphere During the Rainy Season. *J. Atmos. Chem.* **1996**, *25,* 201-214, doi:10.1007/BF00053791.

376. Sanhueza, E.; Donoso, L.; Santana, M.; Fernandez, E.; Romero, J. Atmospheric Chemistry Over the Auyantepuy - (5 Degrees 46 ' N; 62 Degrees 32 ' W; 2100 Meters Asl). *Interciencia* **1999**, *24,* 372-380.

377. Blake, D.R.; Rowland, F.S. Urban Leakage of Liquefied Petroleum Gas and Its Impact on Mexico City Air Quality. *Science* **1995**, *269,* 953-956, doi:10.1126/science.269.5226.953.

378. Franco, J.F.; Pacheco, J.; Belalcazar, L.C.; Behrentz, E. Characterization and Source Identification of VOC Species in Bogata, Columbia. *Amosfera* **2015**, *28,* 1-11, doi:org/10.1016/S0187-6236(15)72155-7.

379. Grosjean, E.; Rasmussen, R.A.; Grosjean, D. Ambient Levels of Gas Phase Pollutants in Porto Alegre, Brazil. *Atmos. Environ.* **1998**, *32,* 3371-3379, doi:org/10.1016/S1352-2310(98)00007-7.

380. Velasco, E.; Lamb, B.; Westberg, H.; Allwine, E.; Sosa, G.; Arriaga-Colina, J.L.; Jobson, B.T.; Alexander, M.L.; Prazeller, P.; Knighton, W.B.; Rogers, T.M.; Grutter, M.; Herndon, S.C.; Kolb, C.E.; Zavala, M.; de Foy, B.; Volkamer, R.; Molina, L.T.; Molina, M.J. Distribution, Magnitudes, Reactivities, Ratios and Diurnal Patterns of Volatile Organic Compounds in the Valley of Mexico During the MCMA 2002 & 2003 Field Campaigns. *Atmos. Chem. Phys.* **2007**, *7,* 329-353, doi:10.5194/acp-7-329-2007.

381. Martins, E.M.; Arbilla, G.; Gatti, L.V. Volatile Organic Compounds in a Residential and Commercial Urban Area With a Diesel, Compressed Natural Gas and Oxygenated Gasoline Vehicular Fleet. *Bull. Environ. Contam. Toxicol.* **2010**, *84,* 175-179, doi:10.1007/s00128-009-9886-2.

382. Alvim, D.S.; Gatti, L.V.; Correa, S.M.; Chiquetto, J.B.; Rossatti, C.S.; Pretto, A.; dos Santos, M.H.; Yamazaki, A.; Orlando, J.P.; Santos, G.M. Main Ozone-Forming VOCs in the City of Sao Paulo: Observations, Modelling and Impacts. *Air Qual. Atmos. Health* **2016**, 1-15, doi:10.1007/s11869-016-0429-9.

383. Apel, E.C.; Emmons, L.K.; Karl, T.; Flocke, F.; Hills, A.J.; Madronich, S.; Lee-Taylor, J.; Fried, A.; Weibring, P.; Walega, J.; Richter, D.; Tie, X.; Mauldin, L.; Campos, T.; Weinheimer, A.; Knapp, D.; Sive, B.; Kleinman, L.; Springston, S.; Zaveri, R.; Ortega, J.; Voss, P.; Blake, D.; Baker, A.; Warneke, C.; Welsh-Bon, D.; de Gouw, J.; Zheng, J.; Zhang, R.; Rudolph, J.; Junkermann, W.; Riemer, D.D. Chemical Evolution of Volatile Organic Compounds in the Outflow of the Mexico City Metropolitan Area. *Atmos. Chem. Phys.* **2010**, *10,* 2353-2375, doi:org/10.5194/acp-10-2353-2010.

384. Zheng, J.; Garzon, J.P.; Huertas, M.E.; Zhang, R.Y.; Levy, M.; Ma, Y.; Huertas, J.I.; Jardon, R.T.; Ruiz, L.G.; Tan, H.B.; Molina, L.T. Volatile Organic Compounds in Tijuana During the Cal-Mex 2010 Campaign: Measurements and Source Apportionment. *Atmos. Environ.* **2013**, *70,* 521-531, doi:org/10.1016/j.atmosenv.2012.11.030.

385. Garzon, J.P.; Huertas, J.I.; Magana, M.; Huertas, M.E.; Cardenas, B.; Watanabe, T.; Maeda, T.; Wakamatsu, S.; Blanco, S. Volatile Organic Compounds in the Atmosphere of Mexico City. *Atmos. Environ.* **2015**, *119,* 415-429, doi:org/10.1016/j.atmosenv.2015.08.014.

386. Menchaca-Torre, H.L.; Mercado-Hernandez, R.; Mendoza-Dominguez, A. Diurnal and Seasonal Variation of Volatile Organic Compounds in the Atmosphere of Monterrey, Mexico. *Atmos. Pollut. Res.* **2015**, *6,* 1073-1081, doi:org/10.1016/j.apr.2015.06.004.

387. Jaimes-Palomera, M.; Retama, A.; Elias-Castro, G.; Neria-Hernandez, A.; Rivera-Hernandez, O.; Velasco, E. Non-Methane Hydrocarbons in the Atmosphere of Mexico City: Results of the 2012 Ozone-Season Campaign. *Atmos. Environ.* **2016**, *132*, 258-275, doi:org/10.1016/j.atmosenv.2016.02.047.

388. Dominutti, P.A.; Nogueira, T.; Borbon, A.; Andrade, M.D.; Fornaro, A. One-Year of NMHCs Hourly Observations in Sao Paulo Megacity: Meteorological and Traffic Emissions Effects in a Large Ethanol Burning Context. *Atmos. Environ.* **2016**, *142*, 371-382, doi:org/10.1016/j.atmosenv.2016.08.008.

389. Elshorbany, Y.F.; Kleffmann, J.; Kurtenbach, R.; Rubio, M.; Lissi, E.; Villena, G.; Gramsch, E.; Rickard, A.R.; Pilling, M.J.; Wiesen, P. Summertime Photochemical Ozone Formation in Santiago, Chile. *Atmos. Environ.* **2009**, *43*, 6398-6407, doi:org/10.1016/j.atmosenv.2009.08.047.

390. Alves, C.; Gomes, P.; Pio, C. Analysis and Interpretation of the Monitoring Data for C2-C11 Hydrocarbons in the Atmosphere of a Rural Portuguese Site. *Int. J. Environ. Pollut.* **2008**, *32*, 341-361, doi:org/10.1504/IJEP.2008.017916.

391. O'Donoghue, R.T.; Broderick, B.M. C2-C6 Background Hydrocarbon Concentrations Monitored at a Roof Top and Green Park Site, in Dublin City Centre. *Environ. Monit. Assess.* **2007**, *132*, 491-501, doi:10.1007/s10661-006-9551-8.

392. Roemer, M.; Builtjes, P.; Esser, P.; Guicherit, R.; Thijsse, T. C2-C5 Hydrocarbon Measurements in the Netherlands 1981-1991. *Atmos. Environ.* **1999**, *33*, 3579-3595, doi:org/10.1016/S1352-2310(97)00130-1.

393. Lightman, P.; Kallend, A.S.; Marsh, A.R.W.; Jones, B.M.R.; Penkett, S.A. Seasonal Variation of Hydrocarbons in the Free Troposphere at Midlatitudes. *Tellus B Chem. Phys. Meteorol.* **1990**, *42*, 408-422, doi:10.1034/j.1600-0889.1990.t01-4-00002.x.

394. Colbeck, I.; Harrison, R.M. The Concentrations of Specific C2-C6 Hydrocarbons in the Air of NW England. *Atmos. Environ.* **1985**, *19*, 1899-1904, doi:org/10.1016/0004-6981(85)90015-0.

395. Puxbaum, H.; Rosenberg, C.; Gregori, M.; Lanzerstorfer, C.; Ober, E.; Winiwarter, W. Atmospheric Concentrations of Formic and Acetic Acid and Related Compounds in Eastern and Northern Austria. *Atmos. Environ.* **1988**, *22*, 2841-2850, doi:org/10.1016/0004-6981(88)90450-7.

396. Bruckmann, P.; Kersten, W.; Funcke, W.; Balfanz, E.; Konig, J.; Theisen, J.; Ball, M.; Papke, O. The Occurrence of Chlorinated and Other Organic Trace Compounds in Urban Air. *Chemosphere* **1988**, *17*, 2363-2380, doi:org/10.1016/0045-6535(88)90147-6.

397. Kanakidou, M.; Bonsang, B.; Lambert, G. Light Hydrocarbons Vertical Profiles and Fluxes in a French Rural Area. *Atmos. Environ.* **1989**, *23*, 921-927, doi:org/10.1016/0004-6981(89)90296-5.

398. Haszpra, L.; Szilagyi, I.; Demeter, A.; Turanyi, T.; Berces, T. Nonmethane Hydrocarbon and Aldehyde Measurements in Budapest, Hungary. *Atmos. Environ. Part A* **1991**, *25*, 2103-2110, doi:org/10.1016/0960-1686(91)90087-N.

399. Solberg, S.; Dye, C.; Schmidbauer, N.; Herzog, A.; Gehrig, R. Carbonyls and Nonmethane Hydrocarbons at Rural European Sites From the Mediterranean to the Arctic. *J. Atmos. Chem.* **1996**, *25*, 33-66, doi:10.1007/BF00053285.

400. Klemp, D.; Kley, D.; Kramp, F.; Buers, H.J.; Pilwat, G.; Flocke, F.; Patz, H.W.; Volz-Thomas, A. Long-Term Measurements of Light Hydrocarbons (C2-C5) at Schauinsland (Black Forest). *J. Atmos. Chem.* **1997**, *28*, 135-171, doi:10.1023/A:1005878018619.

401. Lindskog, A.; Moldanova, J. The Influence of the Origin, Season and Time of the Day on the Distribution of Individual NMHC Measured at Rorvik, Sweden. *Atmos. Environ.* **1994**, *28*, 2383-2398, doi:org/10.1016/1352-2310(94)90390-5.

402. Lofgren, L.; Petersson, G. Photoionization Assessment of C3-C5 Alkadienes and Alkenes in Urban Air. *J. Chromatogr.* **1992**, *591*, 358-361, doi:org/10.1016/0021-9673(92)80253-Q.

403. Boudries, H.; Toupance, G.; Dutot, A.L. Seasonal Variation of Atmospheric Nonmethane Hydrocarbons on the Western Coast of Brittany, France. *Atmos. Environ.* **1994**, *28*, 1095-1112, doi:org/10.1016/1352-2310(94)90287-9.

404. Field, R.A.; Phillips, J.L.; Goldstone, M.E.; Lester, J.N.; Perry, R. Indoor Outdoor Interactions During an Air Pollution Event in Central London. *Environ. Technol.* **1992**, *13*, 391-408, doi:org/10.1080/09593339209385167.

405. Gnauk, T.; Rolle, W. A Three-Year Study of Nonmethane Hydrocarbons in Surface Air Over Saxony (Germany). *J. Atmos. Chem.* **1998**, *30*, 371-395, doi:10.1023/A:1006001020538.

406. Lanz, V.; Hueglin, C.; Buchmann, B.; Hill, M.; Locher, R.; Staehelin, J.; Reimann, S. Receptor Modeling of C2-C7 Hydrocarbon Sources at an Urban Background Site in Zurich, Switzerland: Changes Between 1993-1994 and 2005-2006. *Atmos. Chem. Phys.* **2008**, *8*, 2313-2332, doi:org/10.5194/acp-8-2313-2008.

407. Laurila, T.; Hakola, H. Seasonal Cycle of C2-C5 Hydrocarbons Over the Baltic Sea and Northern Finland. *Atmos. Environ.* **1996**, *30*, 1597-1607, doi:org/10.1016/1352-2310(95)00482-3.

408. Brocco, D.; Fratarcangeli, R.; Lepore, L.; Petricca, M.; Ventrone, I. Determination of Aromatic Hydrocarbons in Urban Air of Rome. *Atmos. Environ.* **1997**, *31*, 557-566, doi:org/10.1016/S1352-2310(96)00226-9.

409. Hansen, A.B.; Palmgren, F. VOC Air Pollutants in Copenhagen. *Sci. Total Environ.* **1996**, *189*, 451-457, doi:org/10.1016/0048-9697(96)05245-X.

410. Moschonas, N.; Glavas, S. C3-C10 Hydrocarbons in the Atmosphere of Athens, Greece. *Atmos. Environ.* **1996**, *30*, 2769-2772, doi:org/10.1016/1352-2310(95)00488-2.

411. Kalabokas, P.; Bartzis, J.G.; Bomboi, T.; Ciccioli, P.; Cieslik, S.; Dlugi, R.; Foster, P.; Kotzias, D.; Steinbrecher, R. Ambient Atmospheric Trace Gas Concentrations and Meteorological Parameters During the First BEMA Measuring Campaign on May 1994 at Castelporziano, Italy. *Atmos. Environ.* **1997**, *31*, 67-77, doi:org/10.1016/S1352-2310(97)00075-7.

412. Veillerot, M.; Locoge, N.; Galloo, J.C.; Guillermo, R. Multidimensional Capillary Gas Chromatography for the Monitoring of Individual Non-Methane Hydrocarbons in Air. *Analusis* **1998**, *26*, M38-M43, doi:org/10.1051/analusis:199826090038.

413. Ramacher, B.; Rudolph, J.; Koppmann, R. Hydrocarbon Measurements in the Spring Arctic Troposphere During the ARCTOC 95 Campaign. *Tellus B Chem. Phys. Meteorol.* **1997**, *49*, 466-485, doi:org/10.3402/tellusb.v49i4.15986.

414. Moschonas, N.; Glavas, S. Non-Methane Hydrocarbons at a High-Altitude Rural Site in the Mediterranean (Greece). *Atmos. Environ.* **2000**, *34*, 973-984, doi:org/10.1016/S1352-2310(99)00205-8.

415. Derwent, R.G.; Davies, T.J.; Delaney, M.; Dollard, G.J.; Field, R.A.; Dumitrean, P.; Nason, P.D.; Jones, B.M.R.; Pepler, S.A. Analysis and Interpretation of the Continuous Hourly Monitoring Data for 26 C2-C8 Hydrocarbons at 12 United Kingdom Sites During 1996. *Atmos. Environ.* **2000**, *34*, 297-312, doi:org/10.1016/S1352-2310(99)00203-4.

416. Sauvage, S.; Plaisance, H.; Locoge, N.; Wroblewski, A.; Coddeville, P.; Galloo, J. Long Term Measurement and Source Apportionment of Non-Methane Hydrocarbons in Three French Rural Areas. *Atmos. Environ.* **2009**, *43,* 2430-2441, doi:org/10.1016/j.atmosenv.2009.02.001.

417. Borbon, A.; Locoge, N.; Veillerot, M.; Galloo, J.C.; Guillermo, R. Characterisation of NMHCs in a French Urban Atmosphere: Overview of the Main Sources. *Sci. Total Environ.* **2002**, *292,* 177-191, doi:org/10.1016/S0048-9697(01)01106-8.

418. Badol, C.; Borbon, A.; Locoge, N.; Leonardis, T.; Galloo, J.C. An Automated Monitoring System for VOC Ozone Precursors in Ambient Air: Development, Implementation and Data Analysis. *Anal. Bioanal. Chem.* **2004**, *378,* 1815-1827, doi:10.1007/s00216-003-2474-0.

419. Borbon, A.; Fontaine, H.; Locoge, N.; Veillerot, M.; Galloo, J.C. Developing Receptor-Oriented Methods for Non-Methane Hydrocarbon Characterisation in Urban Air - Part I: Source Identification. *Atmos. Environ.* **2003**, *37,* 4051-4064, doi:org/10.1016/S1352-2310(03)00525-9.

420. Hopkins, J.R.; Lewis, A.C.; Seakins, P.W. Analysis and Applications of Measurements of Source Dominated Hydrocarbon Concentrations From the PUMA Campaigns in June/July 1999 and January/February 2000 at an Urban Background Site in Birmingham, UK. *Atmos. Environ.* **2005**, *39,* 535-548, doi:org/10.1016/j.atmosenv.2004.09.034.

421. Hakola, H.; Hellen, H.; Laurila, T. Ten Years of Light Hydrocarbons (C2-C6) Concentration Measurements in Background Air in Finland. *Atmos. Environ.* **2006**, *40,* 3621-3630, doi:org/10.1016/j.atmosenv.2005.08.019.

422. Plass-Dulmer, C.; Michl, K.; Ruf, R.; Berresheim, H. C2-C8 Hydrocarbon Measurement and Quality Control Procedures at the Global Atmosphere Watch Observatory Hohenpeissenberg. *J. Chromatogr. A* **2002**, *953,* 175-197, doi:org/10.1016/S0021-9673(02)00128-0.

423. Dollard, G.; Dumitrean, P.; Telling, S.; Dixon, J.; Derwent, R. Observed Trends in Ambient Concentrations of C2-C8 Hydrocarbons in the United Kingdom Over the Period From 1993 to 2004. *Atmos. Environ.* **2007**, *41,* 2559-2569, doi:org/10.1016/j.atmosenv.2006.11.020.

424. Plass-Dulmer, C.; Schmidbauer, N.; Slemr, J.; Slemr, F.; D'Souza, H. European Hydrocarbon Intercomparison Experiment AMOHA Part 4: Canister Sampling of Ambient Air. *J. Geochem. Res. Atmos.* **2006**, *111,* doi:10.1029/2005JD006351.

425. Baroja, O.; Goicolea, M.A.; Sampedro, M.C.; Rodriguez, E.; De Balugera, Z.G.; Alonso, A.; Barrio, R.J. Multisorbent Tubes Sampling Used in Thermal Desorption Cold Trap Injection With Gas Chromatography-Mass Spectrometry for C2-C6 Hydrocarbon Measurements in an Urban Atmosphere. *Int. J. Environ. Anal. Chem.* **2004**, *84,* 341-353, doi:org/10.1080/03067310410001680000.

426. Waked, A.; Sauvage, S.; Borbon, A.; Gauduin, J.; Pallares, C.; Vagnot, M.P.; Leonardis, T.; Locoge, N. Multi-Year Levels and Trends of Non-Methane Hydrocarbon Concentrations Observed in Ambient Air in France. *Atmos. Environ.* **2016**, *141,* 263-275, doi:org/10.1016/j.atmosenv.2016.06.059.

427. Badol, C.; Locoge, N.; Leonardis, T.; Galloo, J.C. Using a Source-Receptor Approach to Characterise VOC Behaviour in a French Urban Area Influenced by Industrial Emissions - Part 1: Study Area Description, Data Set Acquisition and Qualitative Data Analysis of the Data Set. *Sci. Total Environ.* **2008**, *389,* 441-452, doi:org/10.1016/j.scitotenv.2007.09.003.

428. Purvis, R.M.; McQuaid, J.B.; Lewis, A.C.; Hopkins, J.R.; Simmonds, P. Horizontal and Vertical Profiles of Ozone, Carbon Monoxide, Non-Methane Hydrocarbons and Dimethyl Sulphide Near the Mace Head Observatory, Ireland. *Atmos. Chem. Phys. Discuss.* **2005**, *5,* 12505-12530, doi:org/10.5194/acpd-5-12505-2005.

429. Redeker, K.R.; Davis, S.; Kalin, R.M. Isotope Values of Atmospheric Halocarbons and Hydrocarbons From Irish Urban, Rural, and Marine Locations. *J. Geochem. Res. Atmos.* **2007**, *112,* doi:10.1029/2006JD007784.

430. Navazo, M.; Durana, N.; Alonso, L.; Gomez, M.C.; Garcia, J.A.; Ilardia, J.L.; Gangoiti, G.; Iza, J. High Temporal Resolution Measurements of Ozone Precursors in a Rural Background Station. A Two-Year Study. *Environ. Monit. Assess.* **2008**, *136,* 53-68, doi:10.1007/s10661-007-9720-4.

431. Liakakou, E.; Bonsang, B.; Williams, J.; Kalivitis, N.; Kanakidou, M.; Mihalopoulos, N. C2-C8 NMHCs Over the Eastern Mediterranean: Seasonal Variation and Impact on Regional Oxidation Chemistry. *Atmos. Environ.* **2009**, *43,* 5611-5621, doi:org/10.1016/S1352-2310(02)00956-1.

432. Mabilia, R.; Di Palo, V.; Cassardo, C.; Ciuchini, C.; Pasini, A.; Possanzini, M. Measurements of Lower Carbonyls and Hydrocarbons at Ny-Alesund, Svalbard. *Annali di Chimica* **2007**, *97,* 1027-1037.

433. Hopkins, J.R.; Boddy, R.K.; Hamilton, J.F.; Lee, J.D.; Lewis, A.C.; Purvis, R.M.; Watson, N.J. An Observational Case Study of Ozone and Precursors Inflow to South East England During an Anticyclone. *J. Environ. Monit.* **2006**, *8,* 1195-1202, doi:10.1039/B608062F.

434. Gaeggeler, K.; Prevot, A.S.H.; Dommen, J.; Legreid, G.; Reimann, S.; Baltensperger, U. Residential Wood Burning in an Alpine Valley As a Source for Oxygenated Volatile Organic Compounds, Hydrocarbons and Organic Acids. *Atmos. Environ.* **2008**, *42,* 8278-8287, doi:org/10.1016/j.atmosenv.2008.07.038.

435. Evtyugina, M.G.; Nunes, T.; Alves, C.; Marques, M.C. Photochemical Pollution in a Rural Mountainous Area in the Northeast of Portugal. *Atmos. Res.* **2009**, *92,* 151-158, doi:org/10.1016/j.atmosres.2008.09.006.

436. Hellen, H.; Hakola, H.; Haaparanta, S.; Pietarila, H.; Kauhaniemi, M. Influence of Residential Wood Combustion on Local Air Quality. *Sci. Total Environ.* **2008**, *393,* 283-290, doi:org/10.1016/j.scitotenv.2008.01.019.

437. von Schneidemesser, E.; Monks, P.S.; Plass-Duelmer, C. Global Comparison of VOC and CO Observations in Urban Areas. *Atmos. Environ.* **2010**, *44,* 5053-5064, doi:org/10.1016/j.atmosenv.2010.09.010.

438. Fanizza, C.; Incoronato, F.; Baiguera, S.; Schiro, R.; Brocco, D. Volatile Organic Compound Levels at One Site in Rome Urban Air. *Atmos. Pollut. Res.* **2014**, *5,* 303-314, doi:org/10.5094/APR.2014.036.

439. Rudolph, J.; Ehhalt, D.H. Measurements of C2-C5 Hydrocarbons Over the North Atlantic. *J. Geochem. Res. Oceans Atmos.* **1981**, *86,* 1959-1964, doi:10.1029/JC086iC12p11959.

440. van Valin, C.C.; Luria, M. O3, CO, Hydrocarbons and Dimethyl Sulfide Over the Western Atlantic Ocean. *Atmos. Environ.* **1988**, *22,* 2401-2409, doi:org/10.1016/0004-6981(88)90472-6.

441. Saito, T.; Yokouchi, Y.; Kawamura, K. Distributions of C2-C6 Hydrocarbons Over the Western North Pacific and Eastern Indian Ocean. *Atmos. Environ.* **2000**, *34,* 4373-4381, doi:org/10.1016/S1352-2310(00)00249-1.

442. Hopkins, J.R.; Jones, I.D.; Lewis, A.C.; McQuaid, J.B.; Seakins, P.W. Non-Methane Hydrocarbons in the Arctic Boundary Layer. *Atmos. Environ.* **2002**, *36,* 3217-3229, doi:org/10.1016/S1352-2310(02)00324-2.

443. Warneke, C.; de Gouw, J.A. Organic Trace Gas Composition of the Marine Boundary Layer Over the Northwest Indian Ocean in April 2000. *Atmos. Environ.* **2001**, *35,* 5923-5933, doi:org/10.1016/S1352-2310(01)00384-3.

444. Sahu, L.K.; Lal, S.; Venkataramani, S. Seasonality in the Latitudinal Distributions of NMHCs Over Bay of Bengal. *Atmos. Environ.* **2011**, *45,* 2356-2366, doi:org/10.1016/j.atmosenv.2011.02.021.

445. Bonsang, B.; Al Aarbaoui, A.; Sciare, J. Diurnal Variation of Non-Methane Hydrocarbons in the Subantarctic Atmosphere. *Environ. Chem.* **2008**, *5,* 16-23, doi:org/10.1071/EN07018.

446. Sahu, L.K.; Lal, S.; Venkataramani, S. Impact of Monsoon Circulations on Oceanic Emissions of Light Alkenes Over Bay of Bengal. *Global Biogeochem. Cycles* **2010**, *24,* doi:10.1029/2009GB003766.

447. Gilman, J.B.; Kuster, W.C.; Goldan, P.D.; Herndon, S.C.; Zahniser, M.S.; Tucker, S.C.; Brewer, W.A.; Lerner, B.M.; Williams, E.J.; Harley, R.A.; Fehsenfeld, F.C.; Warneke, C.; de Gouw, J.A. Measurements of Volatile Organic Compounds During the 2006 TexAQS/GoMACCS Campaign: Industrial Influences, Regional Characteristics, and Diurnal Dependencies of the OH Reactivity. *J. Geochem. Res. Atmos.* **2009**, *114,* doi:10.1029/2008JD011525.

448. Srivastava, S.; Lal, S.; Venkataramani, S.; Gupta, S.; Sheel, V. Surface Distributions of O3, CO and Hydrocarbons Over the Bay of Bengal and the Arabian Sea During Pre-Monsoon Season. *Atmos. Environ.* **2012**, *47,* 459-467, doi:org/10.1016/j.atmosenv.2011.10.023.

449. Mallik, C.; Lal, S.; Venkataramani, S.; Naja, M.; Ojha, N. Variability in Ozone and Its Precursors Over the Bay of Bengal During Post Monsoon: Transport and Emission Effects. *J. Geochem. Res. Atmos.* **2013**, *118,* 10190-10209, doi:10.1002/jgrd.50764.

450. Nelson, P.F.; Quigley, S.M. Non-Methane Hydrocarbons in the Atmosphere of Sydney, Australia. *Environ. Sci. Technol.* **1982**, *16*, 650-655, doi:10.1021/es00104a005.

451. Schmitt, R.; Volz-Thomas, A. Climatology of Ozone, PAN, CO, and NMHC in the Free Troposphere Over the Southern North Atlantic. *J. Atmos. Chem.* **1997**, *28*, 245-262, doi:10.1023/A:1005801515531.

452. Brunke, E.G.; Labuschagne, C.; Scheel, H.E. Trace Gas Variations at Cape Point, South Africa, During May 1997 Following a Regional Biomass Burning Episode. *Atmos. Environ.* **2001**, *35,* 777-786, doi:org/10.1016/S1352-2310(00)00260-0.

453. Lewis, A.C.; Carpenter, L.J.; Pilling, M.J. Nonmethane Hydrocarbons in Southern Ocean Boundary Layer Air. *J. Geochem. Res. Atmos.* **2001**, *106*, 4987-4994, doi:10.1029/2000JD900634.

454. Read, K.A.; Lewis, A.C.; Salmon, R.A.; Jones, A.E.; Bauguitte, S. OH and Halogen Atom Influence on the Variability of Non-Methane Hydrocarbons in the Antarctic Boundary Layer. *Tellus B Chem. Phys. Meteorol.* **2007**, *59,* 22-38, doi:10.1111/j.1600-0889.2006.00227.x.
